# Inflammatory cytokines and stroke and its subtypes: a genetic correlation and two-sample Mendelian randomization study

**DOI:** 10.3389/fnmol.2023.1294450

**Published:** 2023-11-28

**Authors:** Yu Guo, Huaiyu Sun, Shuai Hou, Wuqiong Zhang, Huiqing Liu, Lixia Zhu, Hongmei Meng

**Affiliations:** Department of Neurology and Neuroscience Center, The First Hospital of Jilin University, Changchun, China

**Keywords:** inflammation, Mendelian randomization, stroke, cytokines, ischemic stroke, intracerebral hemorrhage

## Abstract

**Introduction:**

The causal relationship between inflammatory factors and stroke subtypes remains unclear. This study aimed to analyze the causal relationship between 41 inflammatory factors and these two factors using Mendelian randomization (MR).

**Methods:**

We performed a two-sample MR analysis to assess the causal effects of 41 inflammatory cytokines on stroke and its subtypes and conducted a genome-wide association study (GWAS) data. The inverse-variance weighted (IVW) method was adopted as the main MR method, and we performed a series of two-sample Mendelian randomizations and related sensitivity analyses.

**Results:**

The study indicated some suggestive evidences: using the IVW approach, we found that lower possible levels of IL-4 were positively associated with the occurrence of stroke (odds ratio [OR] = 0.93, 95% confidence interval [CI]: 0.88–0.99, *p* = 0.014), higher interleukin (IL)-1β, IL-12p70 levels may be positively correlated with the occurrence of stroke (OR = 1.09, 95% CI: 1.01–1.18, *p* = 0.027; OR = 1.08, 95% CI: 1.02–1.15, *p* = 0.015). For IS, results showed that lower levels of IL-4, tumor necrosis factor-related apoptosis-inducing ligand were positively associated with the occurrence of possible ischemic stroke (IS) (OR = 0.92, 95% CI: 0.87–0.98, *p* = 0.006; OR = 0.95, 95% CI: 0.91–1.00, *p* = 0.031), higher levels of IL-1β, IL-12p70 and vascular endothelial growth factor (VEGF) may be positively correlated with the occurrence of IS (OR = 1.09, 95% CI: 1.00–1.19, *p* = 0.042; OR = 1.07, 95% CI: 1.01–1.15, *p* = 0.035; OR = 1.06, 95% CI: 1.00–1.12, *p* = 0.034). Our findings suggest that decreased IL-17 levels could potentially be linked to a higher likelihood of intracerebral hemorrhage (ICH) (OR = 0.51, 95% CI: 0.28–0.93, *p* = 0.028). For subtypes of stroke, IS and ICH, higher levels of growth regulated oncogene-α, beta nerve growth factor, IL-18, macrophage colony-stimulating factor, and induced protein 10 upregulated the risk factors while lower levels of IL-2ra and IL-17 upregulated the risk factors.

**Conclusion:**

In summary, our research validated that inflammatory markers have a pivotal impact on the development of stroke and could potentially offer a fresh approach to treating this condition.

## 1 Introduction

Shi et al. (2019) studied stroke including ischemic stroke (IS) and intracerebral hemorrhage (ICH). These are the two most common nervous system emergencies. They are characterized by high incidence, disability, and mortality. Clinical symptoms include aphasia, hemiplegia, hemianopsia, sensory disorders, and cognitive disorders, which can seriously endanger life (Boehme et al., 2017). With the increasing incidence of hypertension and diabetes, the incidence of IS and ICH is increasing yearly (Sun et al., 2019). The etiology of IS is multifactorial, such as embolism of heart origin, cerebral artery stenosis, cerebral atherosclerosis, and immune factors (Campbell et al., 2019). The incidence of ICH is closely related to cerebrovascular malformations, cerebral arteriosclerosis, hypertension, and other factors (Montaño et al., 2021). Immune and inflammatory responses are regulated by cytokines, which are key inflammatory mediators. Cytokines, interferons, growth factors, and interleukins (IL) are among more than 300 that have been identified, including chemokines, interferons, and growth factors (Kelly et al., 2021). A complex network of cytokines influences different functions and activities of the central nervous system by impacting inflammatory cytokines (Zhu et al., 2022). In recent times, numerous researchers have discovered a robust connection between inflammatory cytokines and the IS and ICH. Moreover, the occurrence and recurrence of these conditions are significantly influenced by inflammatory cytokines (Lambertsen et al., 2012).

Inflammatory cytokines are important mediators of the immune inflammatory response induced by ischemic brain injury. They are involved in disease progression and influence the severity and outcome of the disease (Bonaventura et al., 2016). According to previous studies, some inflammatory cytokines are closely related to cerebrovascular disease, such as IL-6 and IL-4 (Zhu et al., 2022). However, whether inflammatory cytokines are the cause of cerebrovascular disease, disease progression, subsequent stress responses, and drug use remains controversial. Although there is an association between inflammatory factors, IS, and ICH in some observational studies, it is possible that the conclusion drawn by these studies were confounded by confounding factors or reverse causality; therefore, definitive causation cannot be obtained.

Mendelian randomization (MR) research is based on the random allocation of alleles during meiosis and can reduce traditional confounders and reverse causality, thus providing better evidence for causal reasoning and overcoming the limitations of observational studies (Burgess et al., 2015; Xiang et al., 2022). We examined whether inflammatory cytokines and stroke subtypes are causally related using a two-sample MR analysis.

## 2 Materials and methods

### 2.1 Study design

**FIGURE 1:**
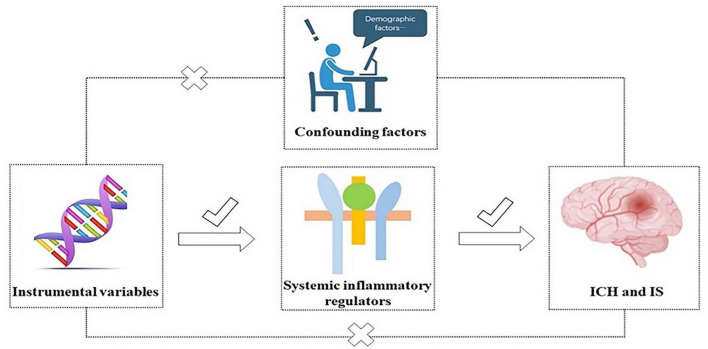
Three core assumptions of inflammatory cytokines with ICH and IS in this two-sample MR study.

To clarify the relationship between systemic inflammatory cytokines and the IS and ICH subtypes, we designed an MR Survey. Figure 1 illustrates the complete study layout. The data we used were obtained from published studies, and this study was approved by the relevant institutional ethics committee. MR should satisfy three core assumptions. Instrumental variables and exposure have a significant correlation (Smith and Ebrahim, 2003). Furthermore, there is no correlation between exposure and outcome in relation to confounding factors (Smith and Ebrahim, 2003). Third, the outcome can only be affected by exposure and not by other means (Emdin et al., 2017).

### 2.2 The selection of data sources and instruments

Inflammatory cytokines were derived from a large-scale genome-wide association study (GWAS) meta-analysis of the circulating concentrations of 41 cytokines in 8,293 randomly selected participants aged 25–74 years in Finland, including the Finnish Youth Cardiovascular Risk Study (YFS), FINRISK 1997, and FINRISK, 2002 (Ahola-Olli et al., 2017). MEGASTROKE Consortium’s meta-analysis of stroke GWAS data provided stroke, IS, and ICH data, which included 40,585 patients and 406,111 controls (Malik et al., 2018). IS subtypes accounted for 34,217 cases, including 4,373 cases of large artery strokes (LAS), 7,193 cases of cardiac embolic strokes (CES), 5,386 cases of small vessel strokes (SVS), and 6,030 cases of lacunar ischemic strokes (LIS). For the ICH, the International Federation of Stroke Genetics provided GWAS data, including 1,545 cases and 1,481 healthy controls (Zou et al., 2023). These cases were divided into two groups: lobal intracerebral hemorrhage (LICH) and non-lobal intracerebral hemorrhage (NLICH) (Table 1; Zou et al., 2023). Instrumental variables, namely single-nucleotide polymorphisms (SNPs), were extracted from the GWAS data of the exposure variables (Chen et al., 2022). We set the whole-genome *p*-value threshold as *p* < 5 × 10^–6^, the linkage disequilibrium threshold as *r*^2^ < 0.001, and distances were 10,000 kb away from each other.

**TABLE 1:** Data for outcome information in GWAS.

Phenotype	Number of SNP	Cases	Controls	Sample size	Population
Outcome stroke	8,211,693	40,585	406,111	446,696	European
ICH	NA	1,545	1,481	3,026	European
LICH	NA	686	1,481	2,167	European
NLICH	NA	909	1,481	2,390	European
IS	7,537,579	34,217	406,111	440,328	European
SVS	6,150,261	5,386	192,662	198,048	European
CES	7,954,834	7,193	406,111	211,763	European
LAS	7,992,739	4,373	406,111	150,765	European
LIS	6,909,434	6,030	248,929	225,419	European

The choices for the IV were unrelated to one another. The F-value indicates the extent of the correlation between IVs and inflammatory regulators; A value of F > 10 is commonly regarded as lacking weak instrumental variables (Wang et al., 2023).

### 2.3 Statistical analysis

In order to investigate the cause-and-effect connection between inflammatory cytokines and stroke and cerebral hemorrhage, we primarily utilized the IVW method as our MR approach for SNP ≥ 2 exposure. This method helps establish if there exists a consistent and dependable causal relationship between exposure and outcome, while also providing supplementary evidence for further understanding. In addition, four MR methods, namely MR-Egger regression, weighted median, weighted mode, and simple mode, were used. The Wald (coefficient ratio) method was used when a single SNP was exposed (Chen et al., 2022). We used a series of sensitivity analyses to assess whether the causal relationships determined by the above methods were heterogeneous and pleiotropic, including MR-Egger, weighted median estimator, MR PRESSO (Burgess et al., 2017). An assessment of heterogeneity among the causal effects of different genetic variants can be made using Cochran’s Q test (Verbanck et al., 2018). Furthermore, we performed a leave-one-out sensitivity test to evaluate the reliability of the MR findings. The results of the pleiotropy and heterogeneity tests were considered statistically significant at *p* < 0.05. The *p*-value was set according to the Bonferroni multiple correction standard. A value of 0.0012 (0.05/41) was considered statistically significant. Suggestive evidence of potential causation was deemed when the *p*-value was less than 0.05, yet exceeded the threshold set by the Bonferroni correction (Georgakis et al., 2019). For this analysis, the TwoSample MR package (version 0.5.7) was used, along with MR-PRESSO in R (version 4.3.0).

## 3 Results

### 3.1 Causal effects of inflammatory cytokines on stroke

**FIGURE 2:**
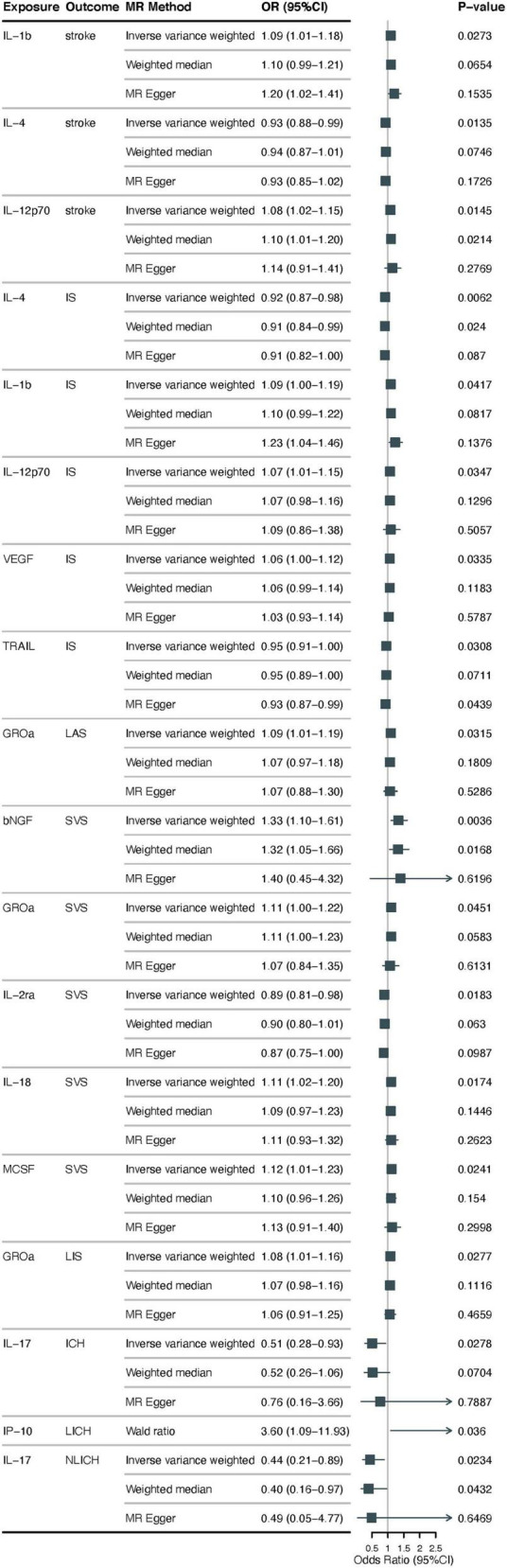
Examining the causal associations between 41 cytokines associated with inflammation and Stroke, including its various subtypes. The alteration in standard deviation of inflammatory cytokines per logarithmic odds increase in stroke and its subtypes is represented by beta and the 95% confidence interval (CI). The *p*-value of 0.05/41 = 0.0012 was deemed statistically significant following adjustment for multiple comparisons. All cytokines, except for IP10, were shown to have results from the inverse-variance weighted method, with the Wald (coefficient ratio) method being considered as the recommended method. IS, ischemic stroke; CES, cardioembolic stroke; SVS, small vessel stroke; LIS, lacunar ischemic stroke; LAS, large artery stroke; ICH, intracerebral hemorrhage; LICH, lobar intracerebral hemorrhage; NLICH, non-lobar intracerebral hemorrhage.

Using the IVW approach, we found that lower possible levels of IL-4 were positively associated with the occurrence of stroke (odds ration [OR] = 0.93, 95% CI: 0.88–0.99, *p* = 0.014), higher IL-1β, IL-12p70 levels may be positively correlated with the occurrence of stroke (OR = 1.09, 95% CI: 1.01–1.18, *p* = 0.027; OR = 1.08, 95% CI: 1.02–1.15, *p* = 0.015). According to MR-Egger Intercept, there was no evidence of horizontal pleiotropy for IL-4, IL-1β, and IL-12p70 (*p* = 0.968; *p* = 0.313; *p* = 0.644, respectively) (Figure 2 and Supplementary Table 1). In addition, the Q values from the MR-Egger and IVW tests indicated the absence of significant heterogeneity for IL-4, IL-1β, and IL-12p70 (all *p*-values > 0.05) (Supplementary Tables 1, 2). The forest, funnel, leave-one-out sensitivity analyses, and scatter plots of Mendelian randomization analyses for IL-4, IL-1β, and IL-12p70 in stroke are shown in Supplementary Figures 1–4.

### 3.2 Causal effects of inflammatory cytokines on IS

Regarding ischemic stroke, using the IVW method, results showed that lower levels of IL-4, TNF-related apoptosis-inducing ligand (TRAIL) were positively associated with the occurrence of possible IS (OR = 0.92, 95% CI: 0.87–0.98, *p* = 0.006; OR = 0.95, 95% CI: 0.91–1.00, *p* = 0.031), higher levels of IL-1β, IL-12p70 and vascular endothelial growth factor (VEGF) may be positively correlated with the occurrence of IS (OR = 1.09, 95% CI: 1.00–1.19, *p* = 0.042; OR = 1.07, 95% CI: 1.01–1.15, *p* = 0.035; and OR = 1.06, 95% CI: 1.00–1.12, *p* = 0.034, respectively). For the subtype LAS, higher growth regulated oncogene-α (CXCL1) (GROα) levels were positively associated with its probable occurrence (OR = 1.09, 95% CI: 1.00–1.19, *p* = 0.034), a subtype of LIS, higher GROα levels were found to be positively correlated with its occurrence (OR = 1.08, 95% CI: 1.01–1.16, *p* = 0.028). In addition, SVS subtype, we found that higher levels of beta nerve growth factor (β-NGF), GROα, IL-18, and macrophage colony-stimulating factor (MCSF) are correlated with the occurrence of SVS (OR = 1.33, 95% CI: 1.10–1.61, *p* = 0.045; OR = 1.11, 95% CI: 1.00–1.22, *p* = 0.017, OR = 1.11, 95% CI: 1.02–1.20, *p* = 0.024; OR = 1.12, 95% CI: 1.01–1.23), low level of IL-2 receptor, alpha submit (IL-2ra) was positively correlated with its occurrence (OR = 0.89, 95% CI: 0.81–0.98, *p* = 0.018). CES, another subtype of ischemic stroke does not have a clear causal relationship with inflammatory cytokine levels. In the above study results, we performed a sensitivity analysis, and MR-Egger showed no evidence of horizontal pleiotropy in the two data sources (*p* > 0.05) (Figure 2 and Supplementary Table 1). The MR-Steiger filter did not detect invalid genetic tools in these analyses. Cochran’s Q test did not detect evidence of heterogeneity (*p* > 0.05) (Supplementary Tables 1, 3–7). Forest, funnel, leave-one-out sensitivity analysis, and scatter plots of Mendelian randomization analyses for suggestive evidence of IS and its subtypes are shown in Supplementary Figures 5–14.

### 3.3 Causal effects of inflammatory cytokines on ICH

Using the IVW method, we found that lower levels of IL-17 may be positively associated with increased risk of ICH (OR = 0.51, 95% CI: 0.28–0.93, *p* = 0.028), for its subtypes LICH and NLICH, higher levels of interferon gamma-induced protein 10 (CXXL10) (IP-10) may be positively correlated with the occurrence of LICH (OR = 3.60, 95% CI: 1.09–11.93, *p* = 0.036), lower levels of IL-17 were likely positively associated with the occurrence of NLICH (OR = 0.44, 95% CI: 0.21–0.89, *p* = 0.023). No evidence of potential horizontal pleiotropy for IL-17 was found by the MR-Egger intercept, with a *p*-value of 0.682 (Figure 2 and Supplementary Table 1). Q values based on the MR-Egger and IVW tests showed no obvious heterogeneity for IL-17 (all *p* > 0.05) (Supplementary Table 8). Supplementary Figure 15 displays the forest, funnel, leave-one-out sensitivity analysis, and scatter plots of the Mendelian randomization analyses conducted on IL-17 levels in ICH. For IP-10, MR-Egger intercept and heterogeneity tests could not be performed because there was only one SNP (Figure 2 and Supplementary Tables 1, 9). The MR-Egger intercept did not identify any evidence of potential horizontal pleiotropy for IL-17 (*p* = 0.935) (Figure 2 and Supplementary Table 1). Cochran’s Q test did not detect any evidence of heterogeneity (*p* > 0.05) (see Supplementary Tables 1, 10). Supplementary Figure 16 displays the sensitivity analysis of IL-17 levels in NLICH using forest plots, funnel plots, leave-one-out analysis, and scatter plots of Mendelian randomization analyses.

## 4 Discussion

Using a GWAS database, we conducted a two-sample Mendelian randomized analysis to investigate the causal association between inflammatory cytokines and stroke, including its subtypes, in a cohort of 41 patients. In this study, some suggestive evidence was obtained: for example, low level of TRAIL, high level of IL-4, IL-1β, IL-12p70, and VEGF may be related to the occurrence of IS, high level of IP-10 may be related to the occurrence of LICH and low level of IL-17 may be related to NLICH. These findings were robust in sensitivity analysis, and our MR results showed a causal relationship between inflammatory factors and stroke risk.

Acute IS and ICH affect millions of people worldwide. Previous research has indicated that stroke leads to acute focal brain injury. As the ischemic time prolongs, the damaged nerve cells produce a large amount of reactive oxygen species, and glial cells secrete a large amount of cytokines, leading to nerve cell death and the destruction of the blood-brain barrier. Immune cells activated in the periphery accumulate in the ischemic brain tissue through the damaged blood-brain barrier (Zhang et al., 2017; Toman et al., 2019; Maida et al., 2020; Xu et al., 2020). Although the pathogenesis of the primary injury is different, the activation of the immune response occurs similarly in IS and ICH. Damaged cells release damage-associated molecular patterns that trigger the immune response, which mediate the activation of intracellular signaling pathways through a series of complex processes (Xu et al., 2020). ILs play a bidirectional role in the occurrence and development of IS (Zhang et al., 2021). On the one hand, it can mediate the activation, reproduction, and differentiation of T and B cells, and on the other hand, it can activate and regulate the inflammatory response of inflammatory cells through signal transduction (Bonaventura et al., 2016; Tschoe et al., 2020; Zhu et al., 2022). Among them, IL-4 increases significantly during the acute phase of stroke, promoting the polarization of M2 microglia, inhibiting pro-inflammatory cytokines, and affecting neuronal excitability (Jiang et al., 2020). It has been reported that infarct size after an ischemic stroke can be reduced by IL-4 secreted by M2 microglia and can contribute to recovery postmortem (Liu et al., 2016). After activation of microglia by IL-4, it can accelerate angiogenesis by secreting extraneous bodies containing miRNA-26a, thereby alleviating IS damage (Bonaventura et al., 2016; Zhao et al., 2022).

IL-1β is a pro-inflammatory cytokine with neurotoxic effects although other inflammatory factors may cause stroke. Research has indicated that elevated release of IL-1β has the ability to trigger phospholipase A2, causing the breakdown of arachidonic acid and disruption of the phospholipid bilayer, ultimately resulting in dysfunction of the blood-brain barrier. The interaction between IL-1β and vascular endothelium can enhance the adherence of leukocytes. At the same time, IL-1β can enhance ischemic injury by activating the molecular mechanism of apoptosis, leading to the apoptosis of damaged cells (Barthels and Das, 2020). Most cell types, such as neurons, astrocytes, and endothelial cells, secrete VEGF, which is believed to play a role in angiogenesis and the permeability of blood vessels. Research has indicated that VEGF is significantly stimulated in the hyperacute stage of cerebral ischemia, contributing not only to enhancing vascular permeability and facilitating angiogenesis but also to playing a crucial part in neuroprotection (Wang et al., 2022). Microglia release TRAIL, a member of the TNF superfamily. Experimental evidence suggests that TRAIL exerts its neuroprotective effect by increasing the expression of TRAIL bait receptors and decreasing the expression of TRAIL itself and its death receptors (Cantarella et al., 2014). GROα is a pro-inflammatory chemokine that interacts with other cytokines and expresses adhesion molecules after stroke, thus promoting white blood cell migration to ischemic sites (Ormstad et al., 2011). The secretion of IL-17 by Th17 cells stimulates T-cell activation by causing the production of different inflammatory cytokines, leading to a strong inflammatory reaction. This reaction could potentially be a significant factor in the development of ICH (Zhang et al., 2021). The interaction between cytokines and inflammatory cells, as well as the interaction among cytokines, can impact the onset and progression of stroke, and even influence the outcome of stroke patients (Lambertsen et al., 2019). Inflammatory factors play important roles in the immune mechanisms underlying stroke.

Our study had some limitations. First, our GWAS data were from a European population, and it remains to be proven whether the results of this study are consistent across populations in different regions. Second, we used relatively high thresholds (*p* < 5 × 10^–6^) when extracting IVs, and there may be a weak IV bias. However, given the F-statistics, we can ignore the effect of weak IVs on the results. In addition, we investigated only the association between systemic inflammation and neuroinflammation, which may have different effects on stroke and its subtypes. Thus, obtaining high-quality neuroinflammatory GWAS will be valuable in the future. Finally, in our MR Analysis, we were unable to analyze all inflammatory cytokines due to the exclusion criteria and previous GWAS, which had a limited number of these cytokines.

## 5 Conclusion

In conclusion, our findings further confirm that inflammatory factors play a crucial role in the occurrence of stroke and may provide a new direction for stroke treatment.

## Data availability statement

The original contributions presented in this study are included in this article/Supplementary material, further inquiries can be directed to the corresponding author.

## Ethics statement

Ethical review and approval was not required for the study on human participants in accordance with the local legislation and institutional requirements. Written informed consent from the patients/participants or patients/participants’ legal guardian/next of kin was not required to participate in this study in accordance with the national legislation and the institutional requirements.

## Author contributions

YG: Writing – original draft, Writing – review and editing. HS: Investigation, Methodology, Writing – review and editing. SH: Formal analysis, Writing – original draft. WZ: Formal analysis, Writing – review and editing. HL: Data curation, Writing – review and editing. LZ: Data curation, Writing – review and editing. HM: Project administration, Supervision, Writing – review and editing.
