# Supplementary material for: Inflammatory cytokines and stroke and its subtypes: a genetic correlation and two-sample Mendelian randomization study
Source: Front Mol Neurosci. 2023 Nov 28;16:1294450. doi: 10.3389/fnmol.2023.1294450 (PMC10713998; doi:10.3389/fnmol.2023.1294450)
Supplement: Supplementary file 11 [file Data_Sheet_1.docx]

Supplementary Material

Inflammatory cytokines and stroke and its subtypes: A Genetic Correlation and Two-Sample Mendelian Randomization Study

Yu Guo^1^, Huaiyu Sun^1^, Shuai Hou^1^, Wuqiong Zhang^1^, Huiqing Liu^1^, Lixia Zhu^1^, Hongmei Meng^1^*

*** Correspondence:** Hongmei Meng: [menghm@jlu.edu.cn](mailto:menghm@jlu.edu.cn)

# Supplementary Tables

**Supplementary Table S1. Results of the MR study testing causal association between inflammatory cytokines and stroke and** **its subtypes.**

**Supplementary Table S2. The Association Between Inflammatory Cytokines and Stroke. Sheet1:** Characteristics of the genetic instrument variables for the cytokines in the Mendelian randomization study at level P < 5 × 10^–6^. **Sheet 2:** MR analysis of 41 cytokines and Stroke risk. **Sheet 3:** Heterogeneity analysis of 41 cytokines and Stroke risk. **Sheet 4:** MR Egger intercept analysis of the association between 41 cytokines and Stroke risk.

**Supplementary Table S3. The Association Between Inflammatory Cytokines and IS. Sheet1:** Characteristics of the genetic instrument variables for the cytokines in the Mendelian randomization study at level P < 5 × 10^–6^. **Sheet 2:** MR analysis of 41 cytokines and IS risk. **Sheet 3:** Heterogeneity analysis of 41 cytokines and IS risk. **Sheet 4:** MR Egger intercept analysis of the association between 41 cytokines and IS risk.

**Supplementary Table S4. The Association Between Inflammatory Cytokines and LAS. Sheet1:** Characteristics of the genetic instrument variables for the cytokines in the Mendelian randomization study at level P < 5 × 10^–6^. **Sheet 2:** MR analysis of 41 cytokines and LAS risk. **Sheet 3:** Heterogeneity analysis of 41 cytokines and LAS risk. **Sheet 4:** MR Egger intercept analysis of the association between 41 cytokines and LAS risk.

**Supplementary Table S5. The Association Between Inflammatory Cytokines and SVS. Sheet1:** Characteristics of the genetic instrument variables for the cytokines in the Mendelian randomization study at level P < 5 × 10^–6^. **Sheet 2:** MR analysis of 41 cytokines and SVS risk. **Sheet 3:** Heterogeneity analysis of 41 cytokines and SVS risk. **Sheet 4:** MR Egger intercept analysis of the association between 41 cytokines and SVS risk.

**Supplementary Table S6. The Association Between Inflammatory Cytokines and LIS. Sheet1:** Characteristics of the genetic instrument variables for the cytokines in the Mendelian randomization study at level P < 5 × 10^–6^. **Sheet 2:** MR analysis of 41 cytokines and LIS risk. **Sheet 3:** Heterogeneity analysis of 41 cytokines and LIS risk. **Sheet 4:** MR Egger intercept analysis of the association between 41 cytokines and LIS risk.

**Supplementary Table S7. The Association Between Inflammatory Cytokines and CES. Sheet1:** Characteristics of the genetic instrument variables for the cytokines in the Mendelian randomization study at level P < 5 × 10^–6^. **Sheet 2:** MR analysis of 41 cytokines and CES risk. **Sheet 3:** Heterogeneity analysis of 41 cytokines and CES risk. **Sheet 4:** MR Egger intercept analysis of the association between 41 cytokines and CES risk.

**Supplementary Table S8. The Association Between Inflammatory Cytokines and ICH. Sheet1:** Characteristics of the genetic instrument variables for the cytokines in the Mendelian randomization study at level P < 5 × 10^–6^. **Sheet 2:** MR analysis of 41 cytokines and ICH risk. **Sheet 3:** Heterogeneity analysis of 41 cytokines and ICH risk. **Sheet 4:** MR Egger intercept analysis of the association between 41 cytokines and ICH risk.

**Supplementary Table S9. The Association Between Inflammatory Cytokines and LICH. Sheet1:** Characteristics of the genetic instrument variables for the cytokines in the Mendelian randomization study at level P < 5 × 10^–6^. **Sheet 2:** MR analysis of 41 cytokines and LICH risk. **Sheet 3:** Heterogeneity analysis of 41 cytokines and LICH risk. **Sheet 4:** MR Egger intercept analysis of the association between 41 cytokines and LICH risk.

**Supplementary Table S10. The Association Between Inflammatory Cytokines and NLICH. Sheet1:** Characteristics of the genetic instrument variables for the cytokines in the Mendelian randomization study at level P < 5 × 10^–6^. **Sheet 2:** MR analysis of 41 cytokines and NLICH risk. **Sheet 3:** Heterogeneity analysis of 41 cytokines and NLICH risk. **Sheet 4:** MR Egger intercept analysis of the association between 41 cytokines and NLICH risk.

# Supplementary Figures


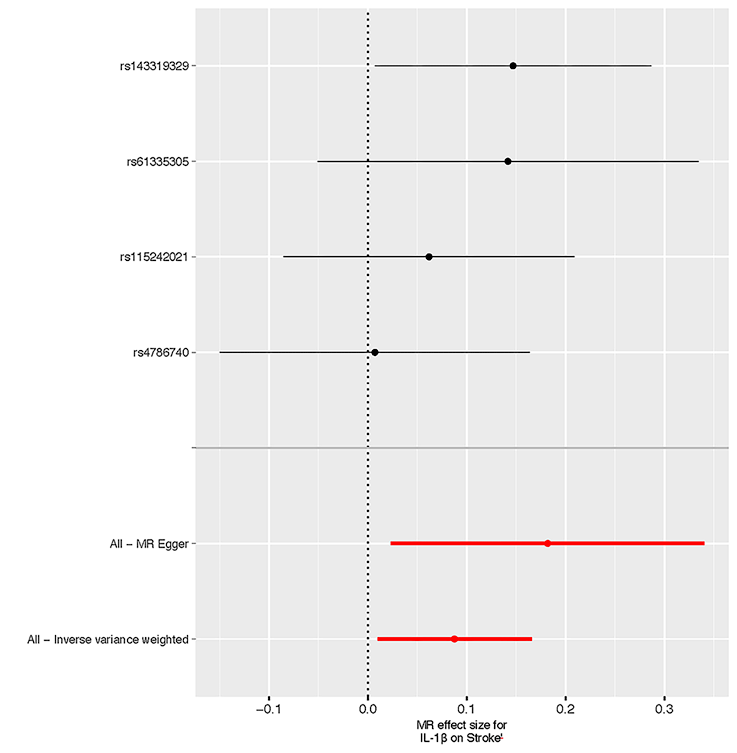
A**
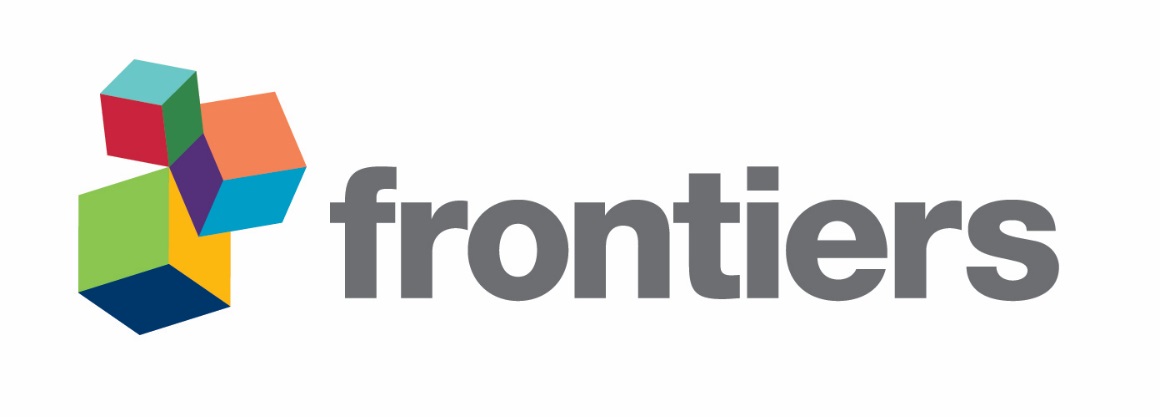
**B
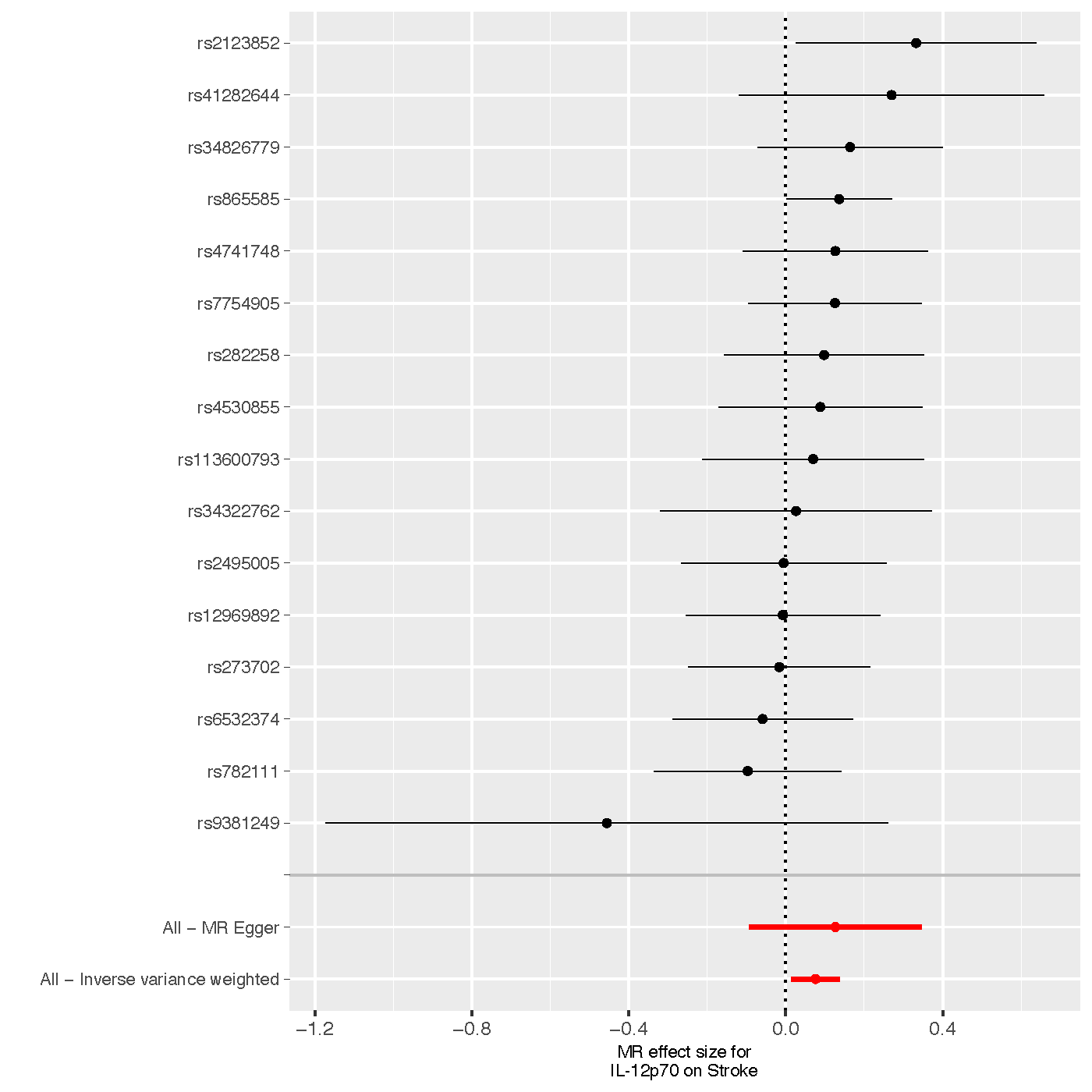


C

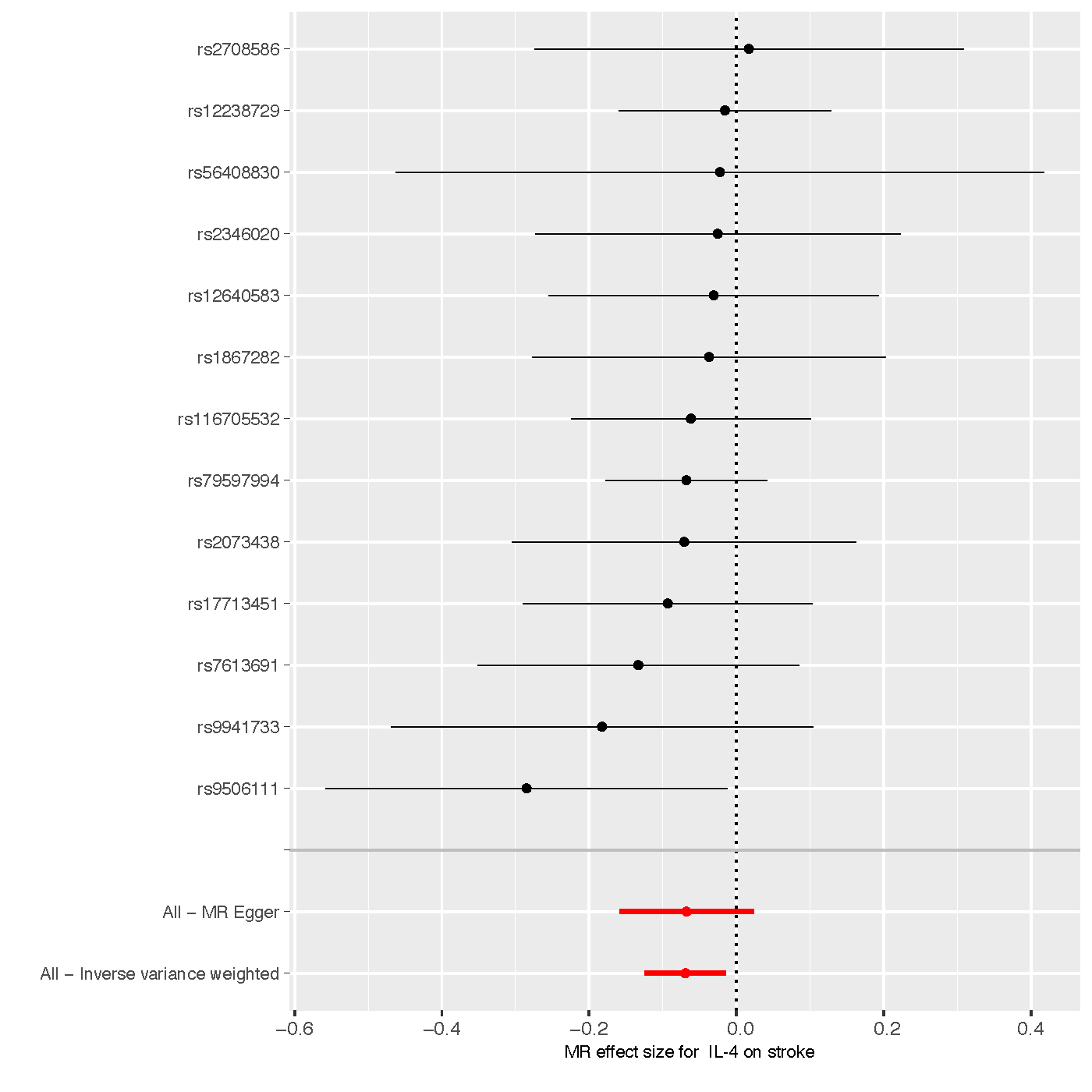


**Supplementary Figure 1.** (A)Forest plots for the exposure of IL-1β (B) Forest plots for the exposure of IL-12p70 (C) Forest plots for the exposure of IL-4

A B


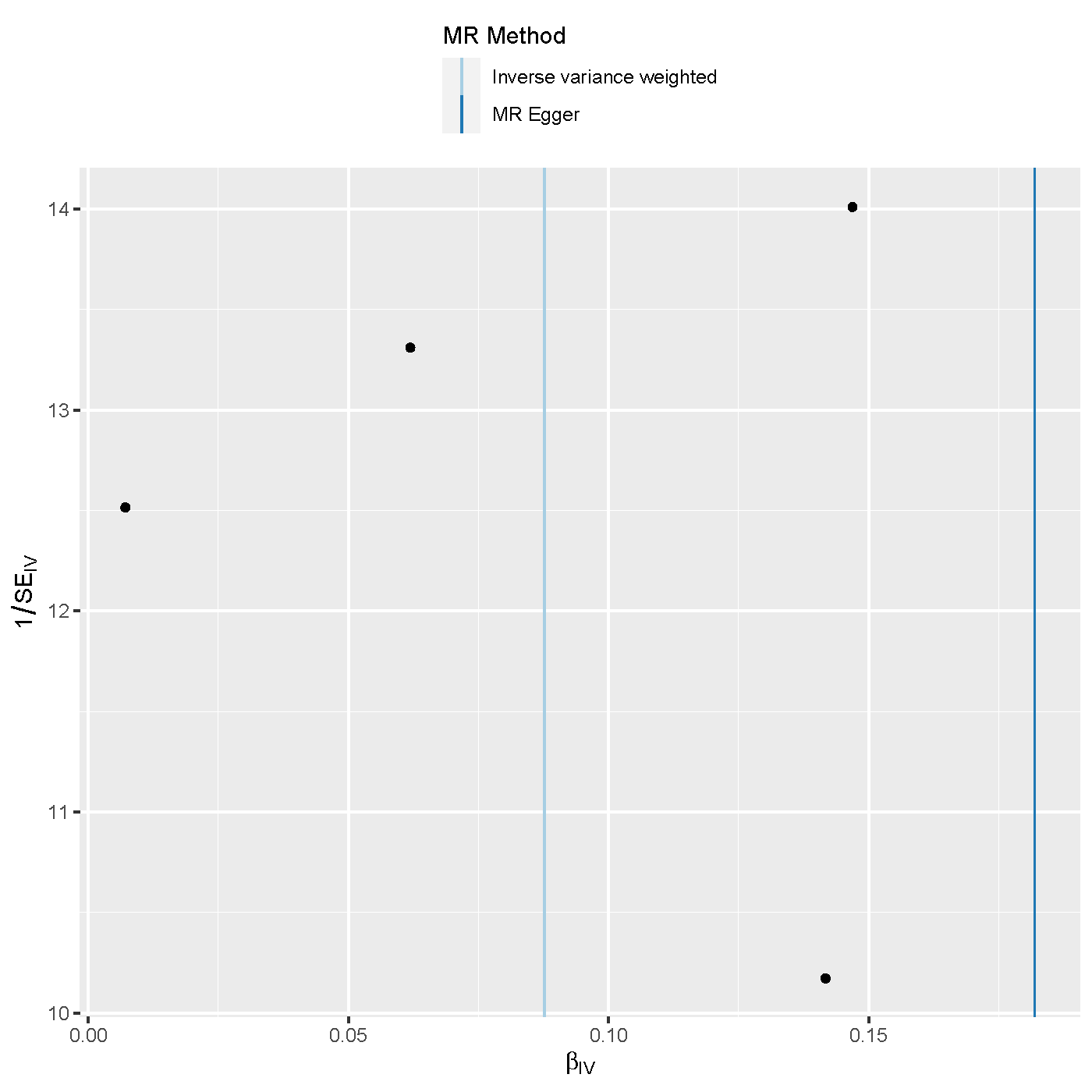

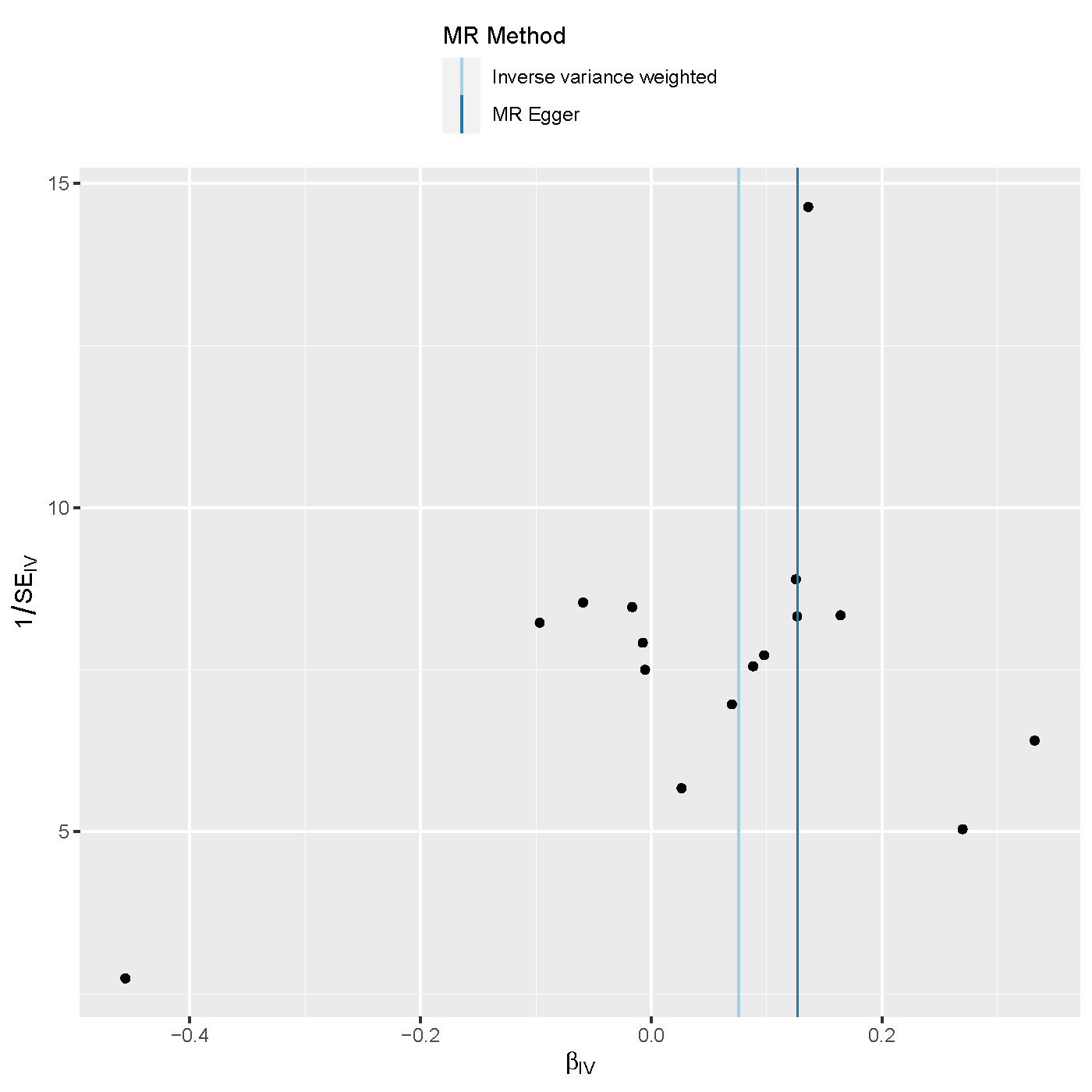


C


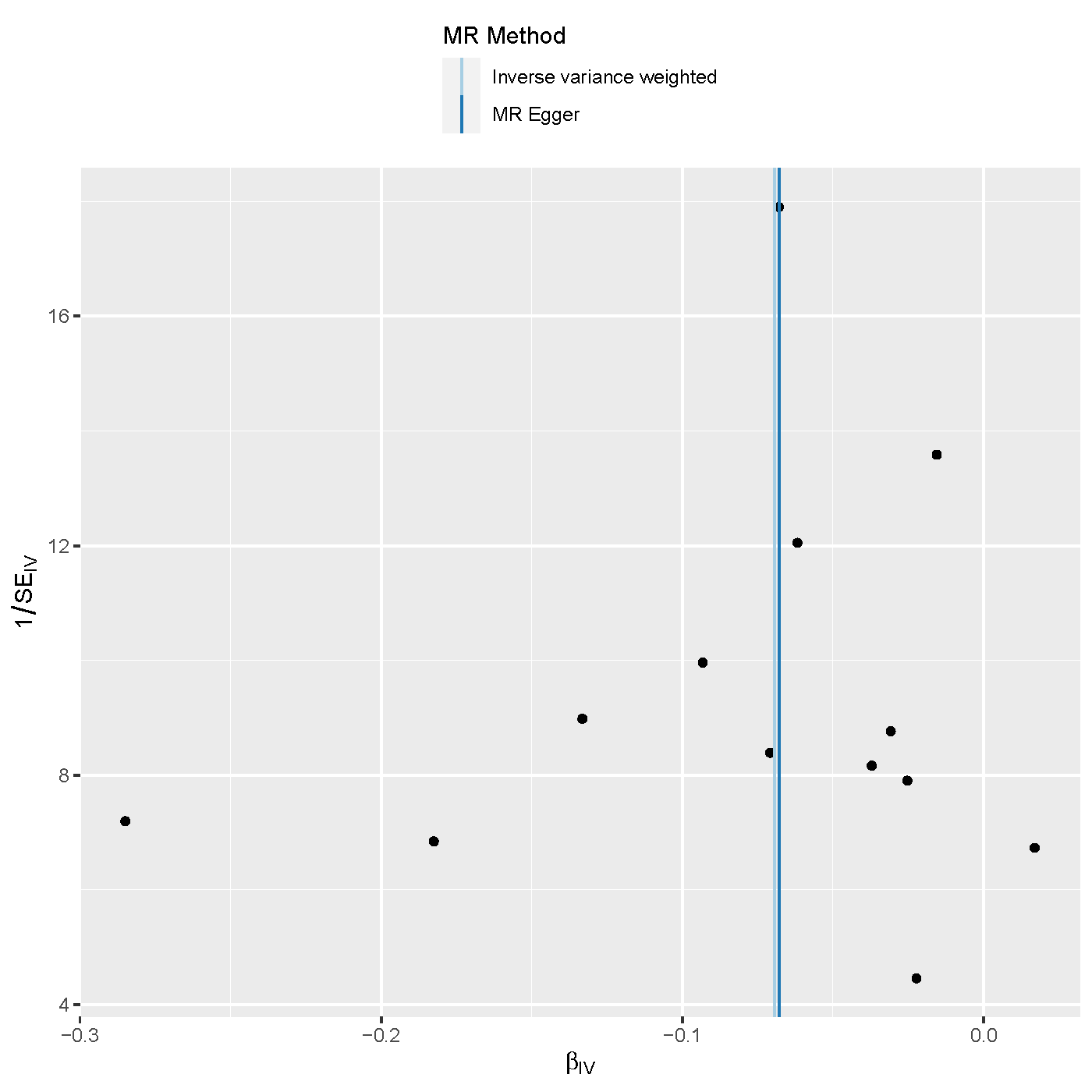


**Supplementary Figure 2.** (A) Funnel plots for the exposure of IL-1β (B) Funnel plots for the exposure of IL-12p70 (C) Funnel plots for the exposure of IL-4

A B


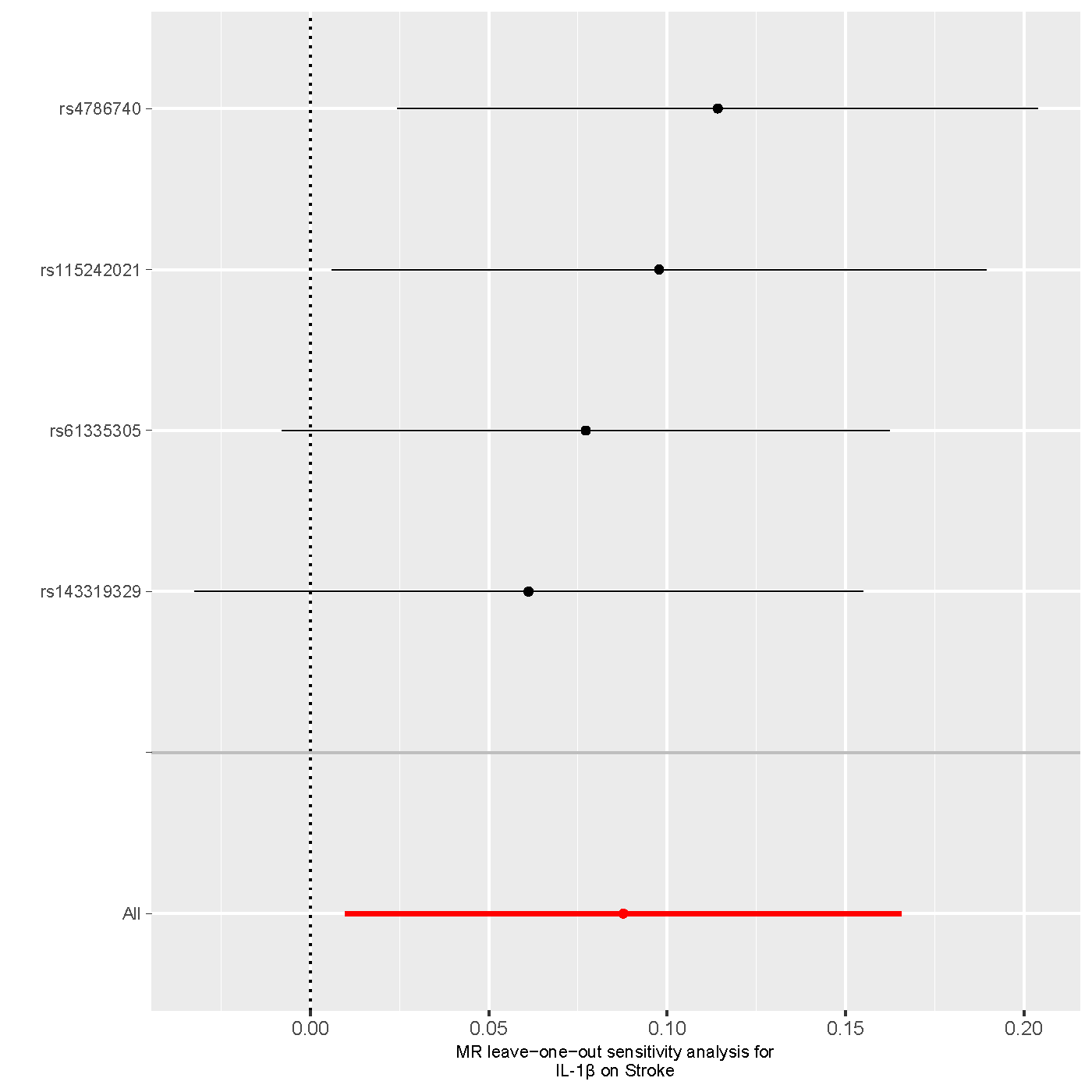

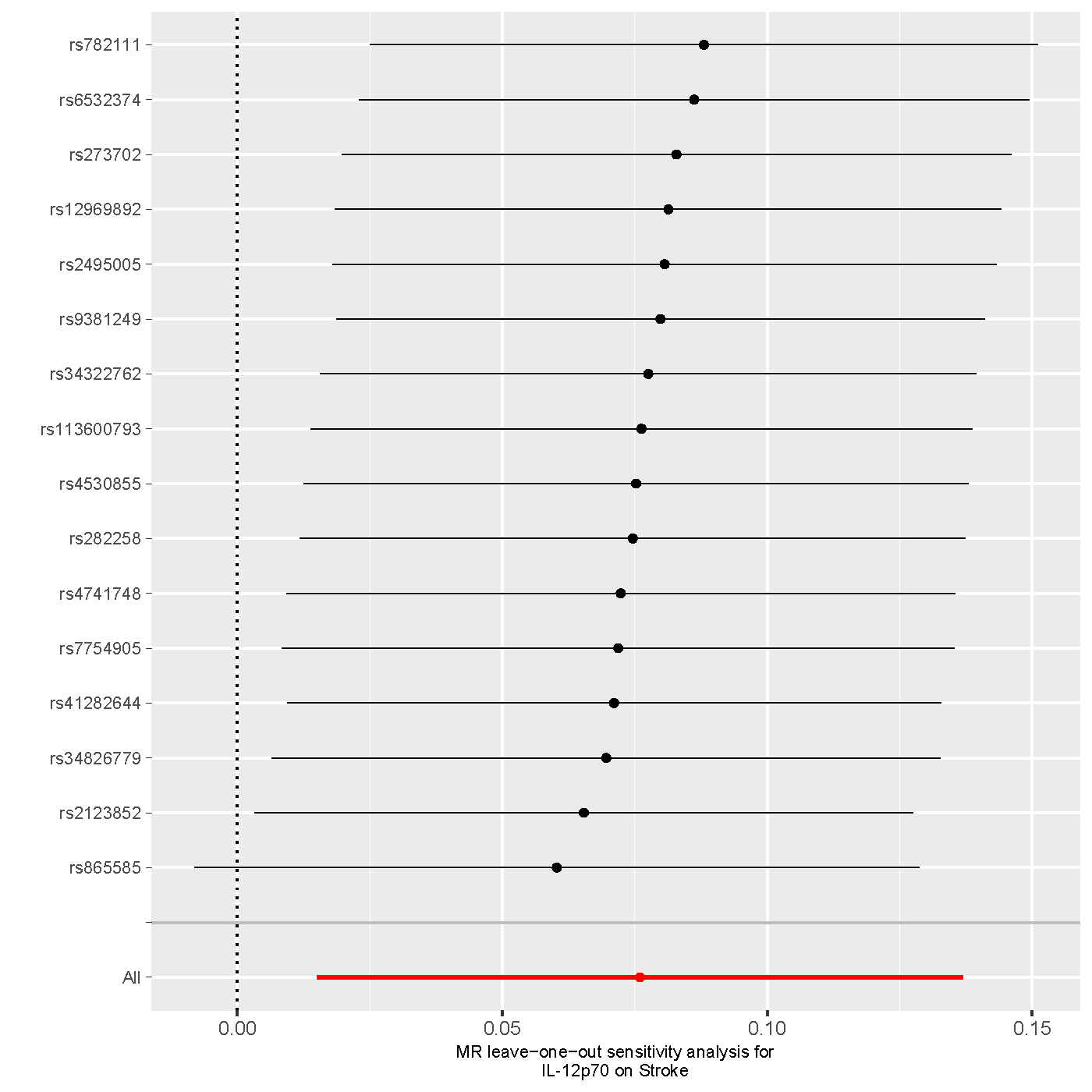


C


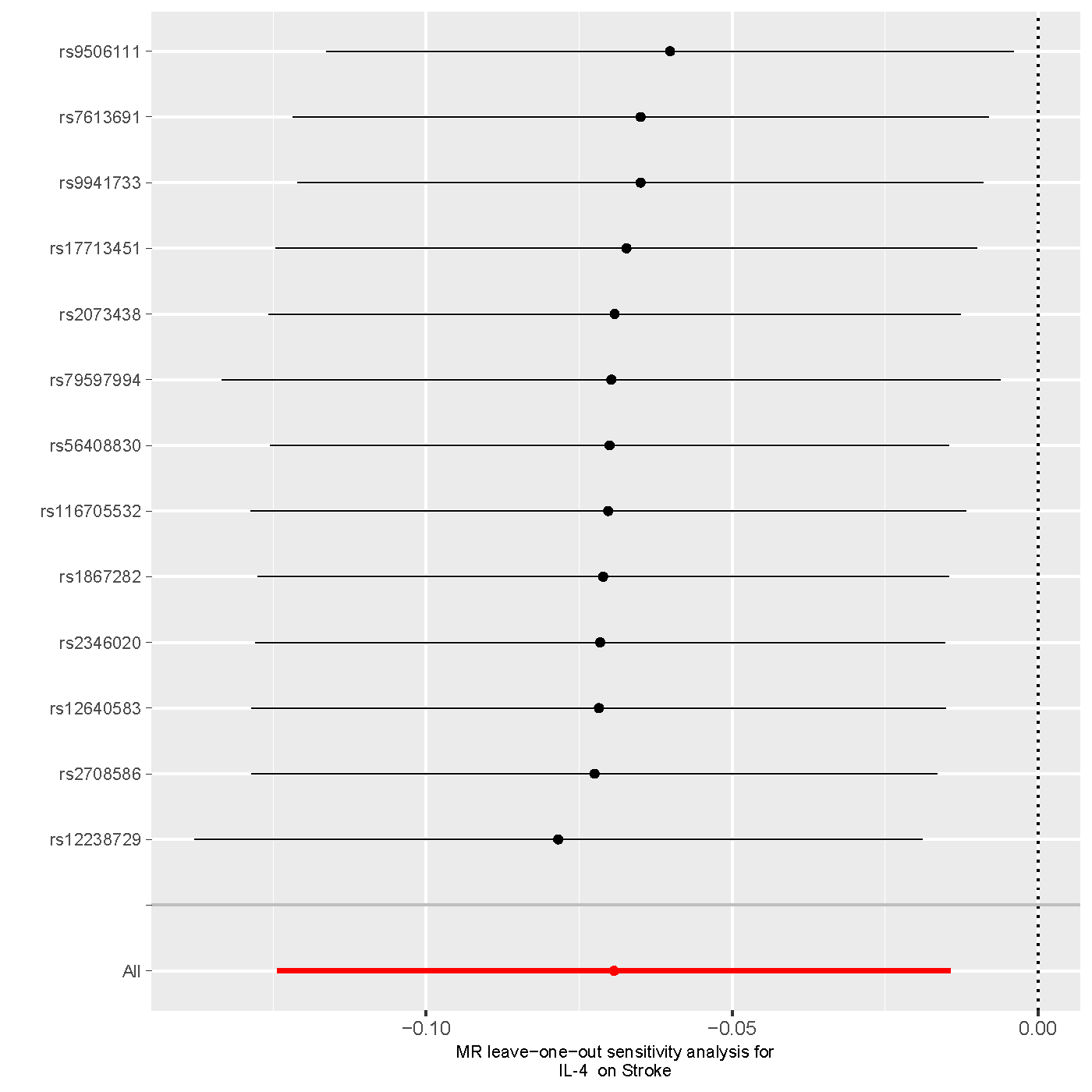


**Supplementary Figure 3.** (A) Leave-one-out plots for the exposure of IL-1β (B) Leave-one-out plots for the exposure of IL-12p70 (C) Leave-one-out plots for the exposure of IL-4

**A B**


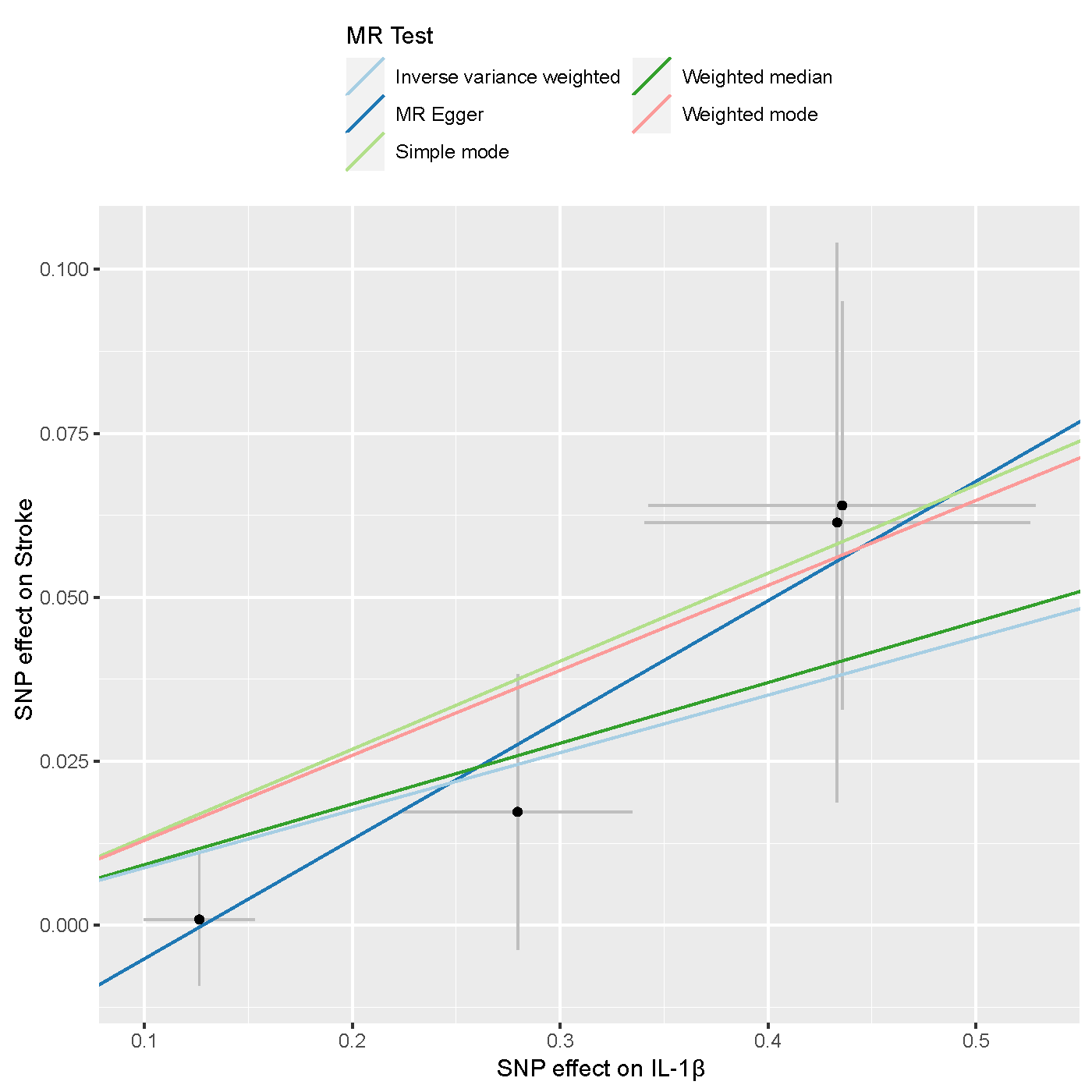


**C**


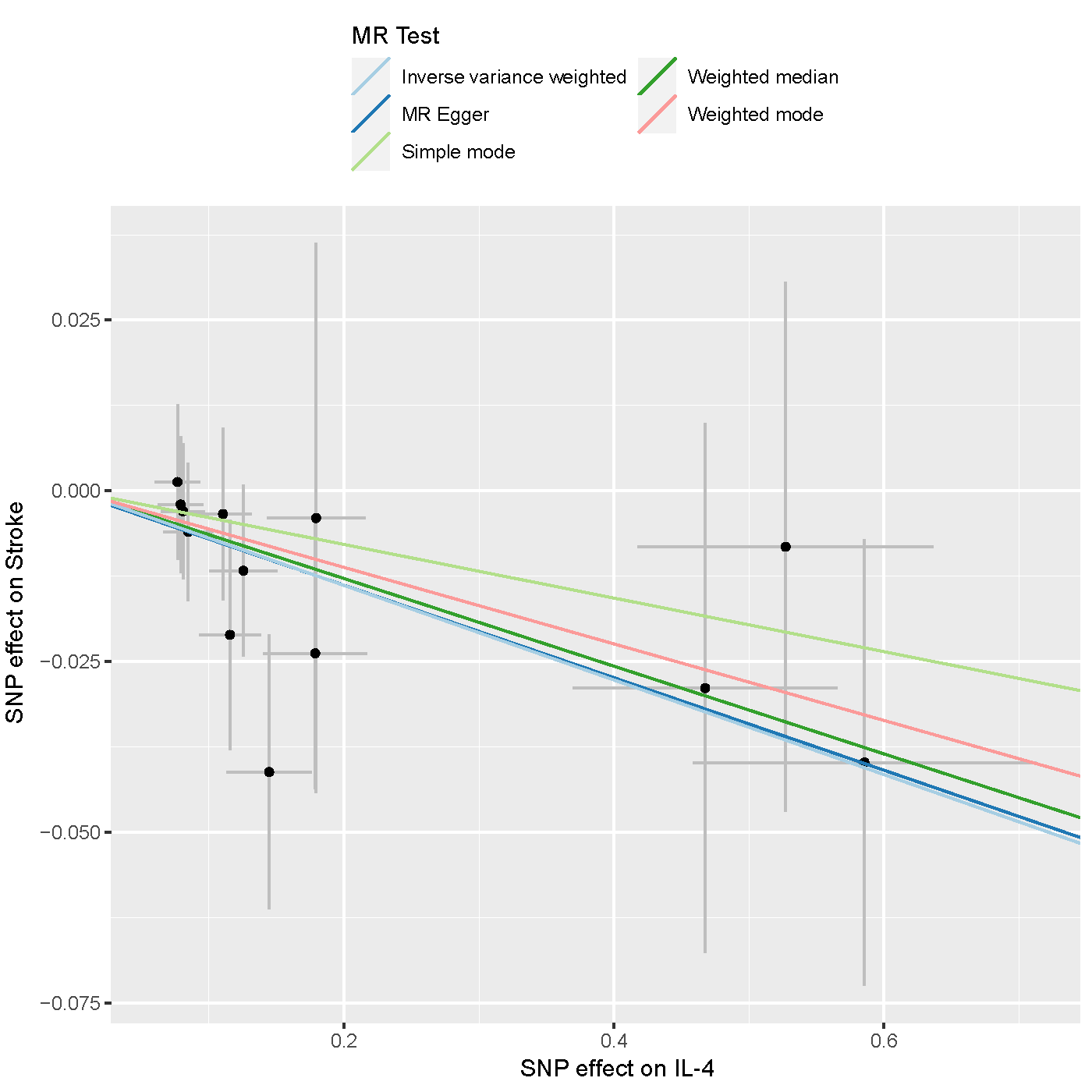


**Supplementary Figure 4.** (A) Scatter plots for the exposure of IL-1β (B) Scatter plots for the exposure of IL-12p70 (C) Scatter plots for the exposure of IL-4

**A B**


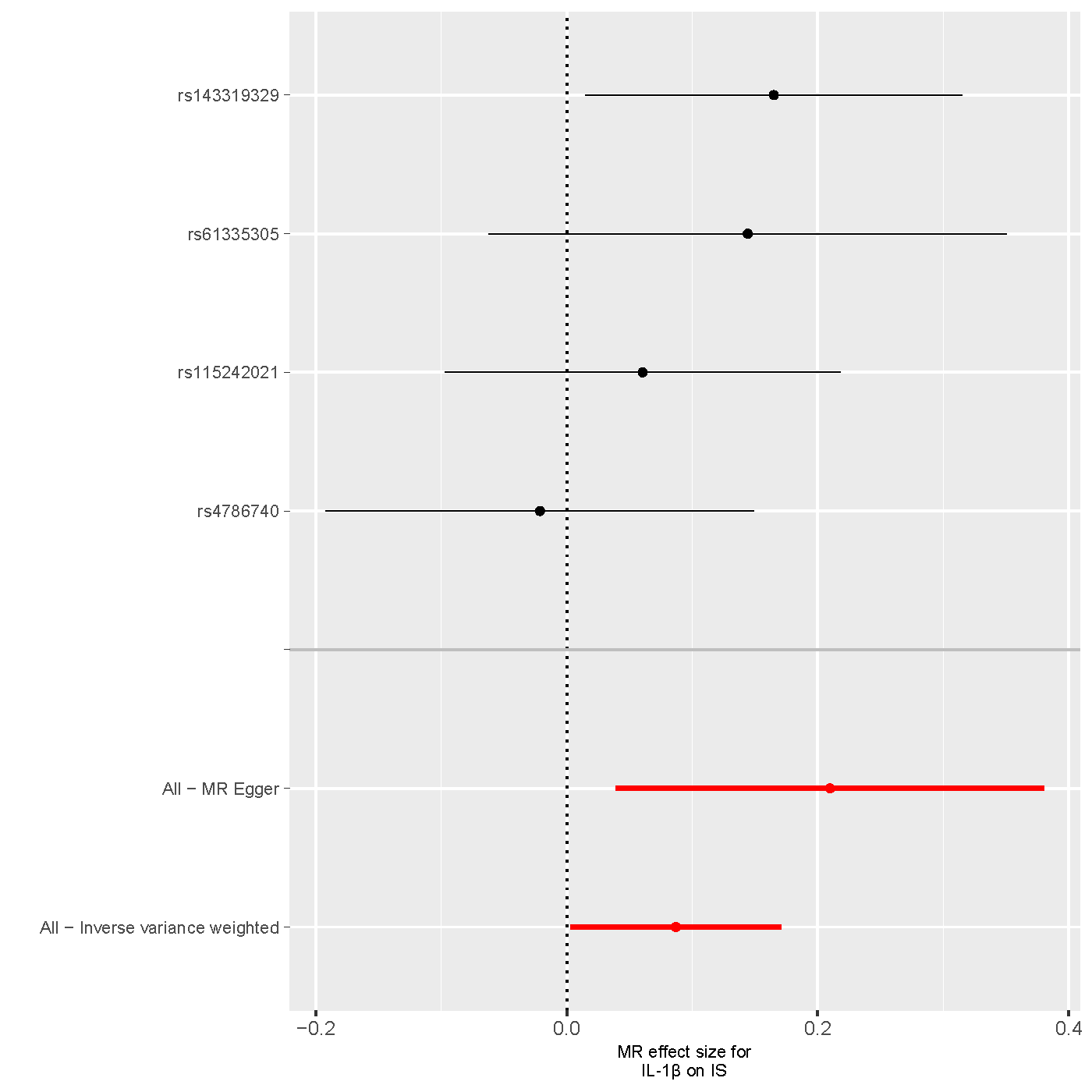


**C D**


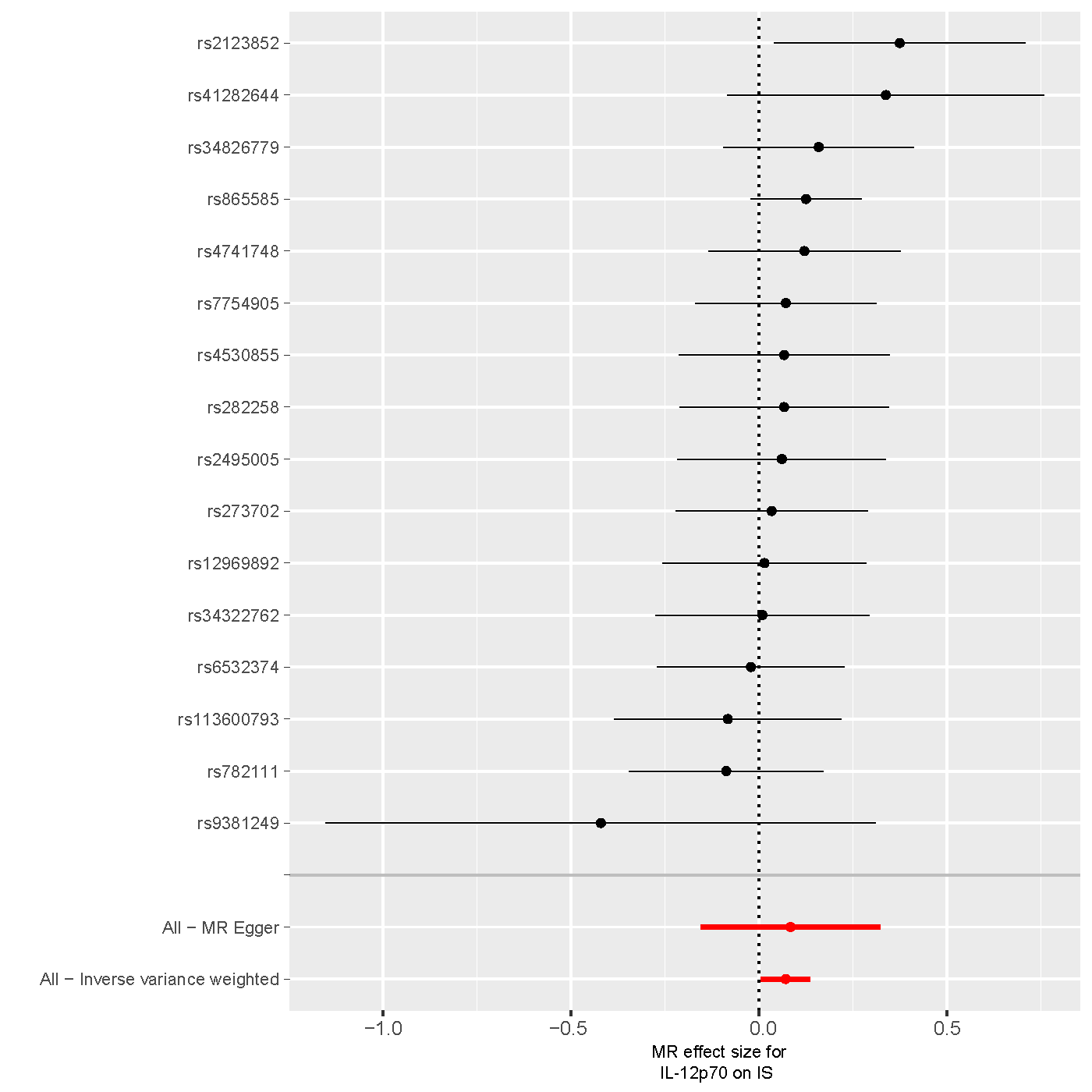

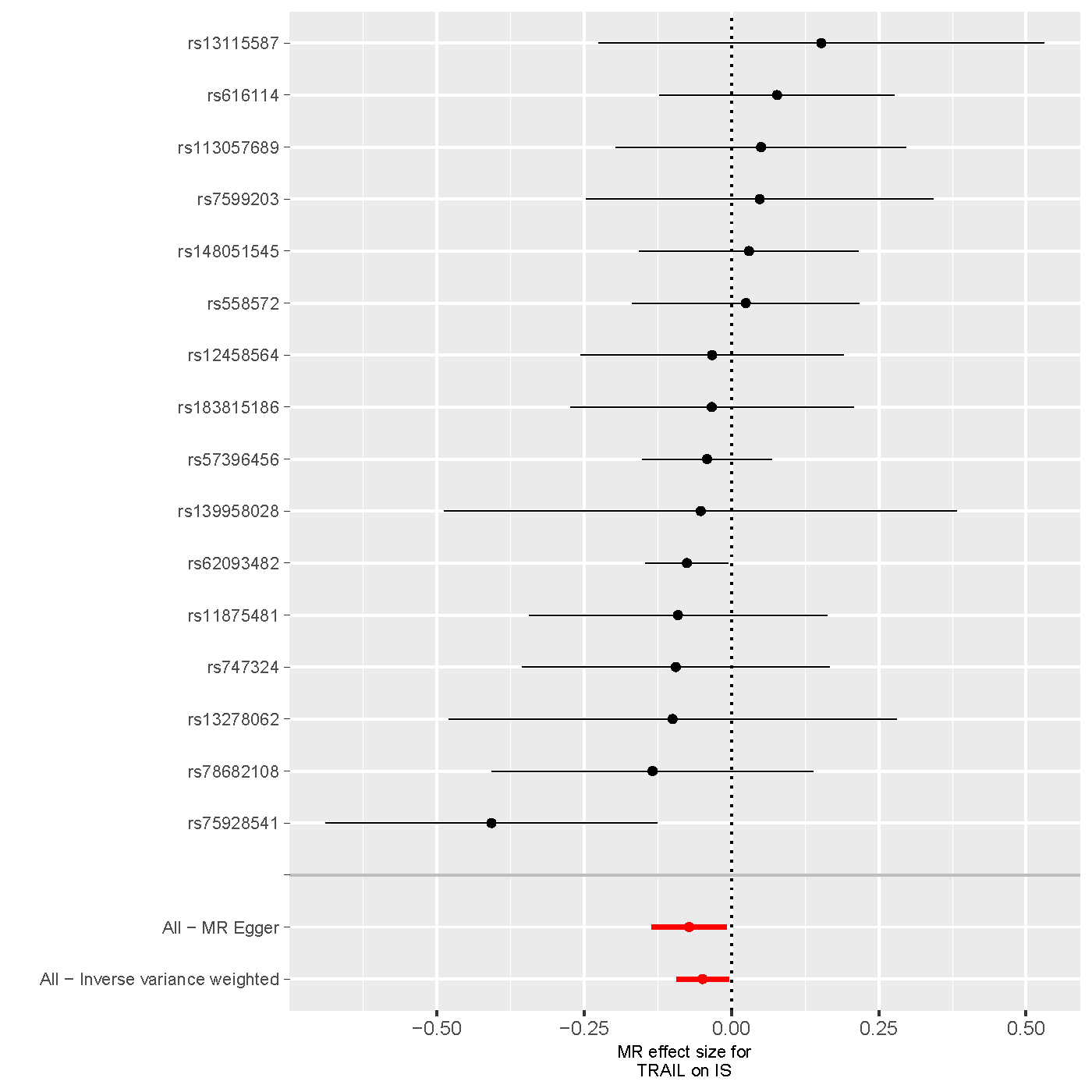


**E**


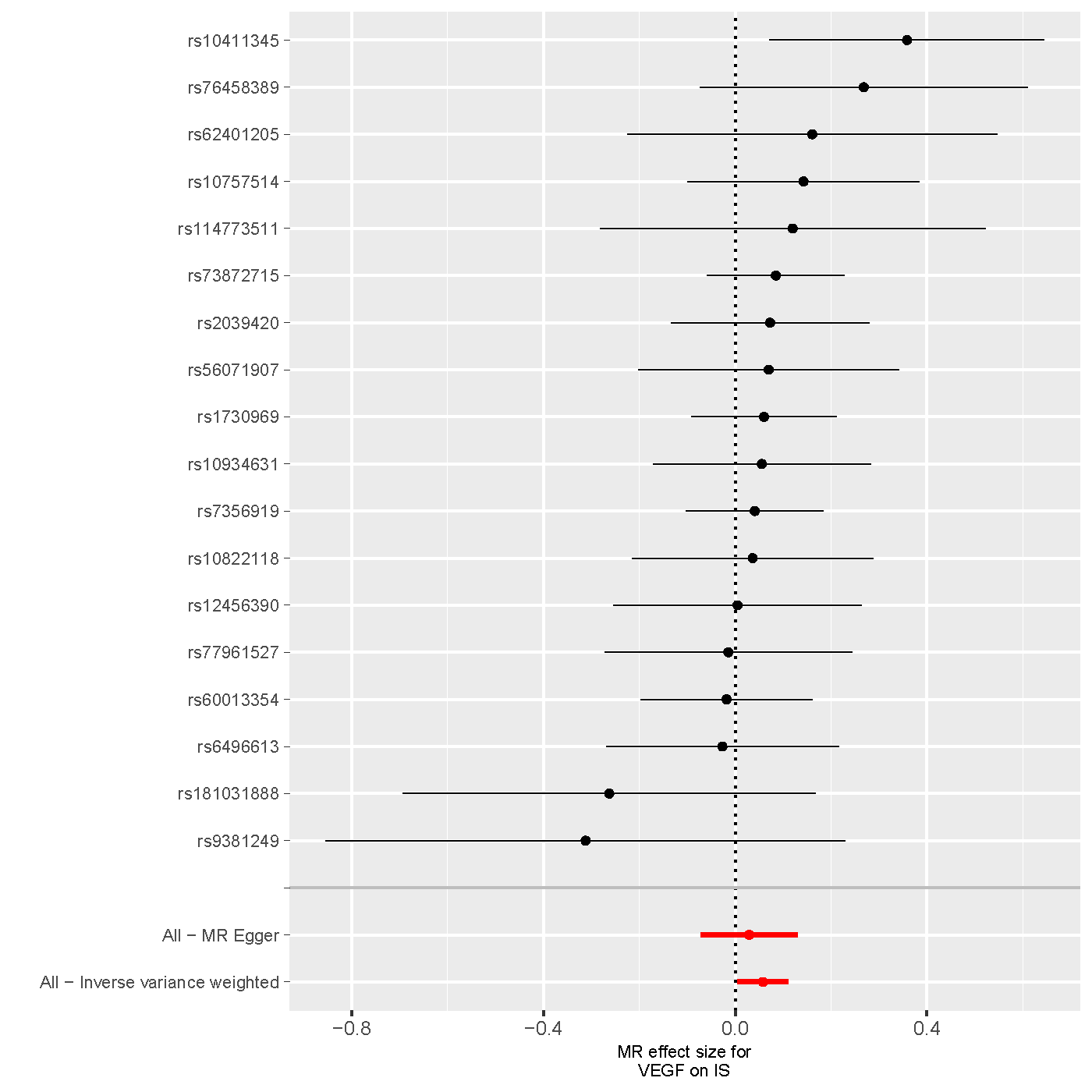


**Supplementary Figure 5.** (A)Forest plots for the exposure of IL-1β (B) Forest plots for the exposure of IL-4 (C) Forest plots for the exposure of IL-12p70(D) Forest plots for the exposure of TRAIL(E) Forest plots for the exposure of VEGF

**A B**


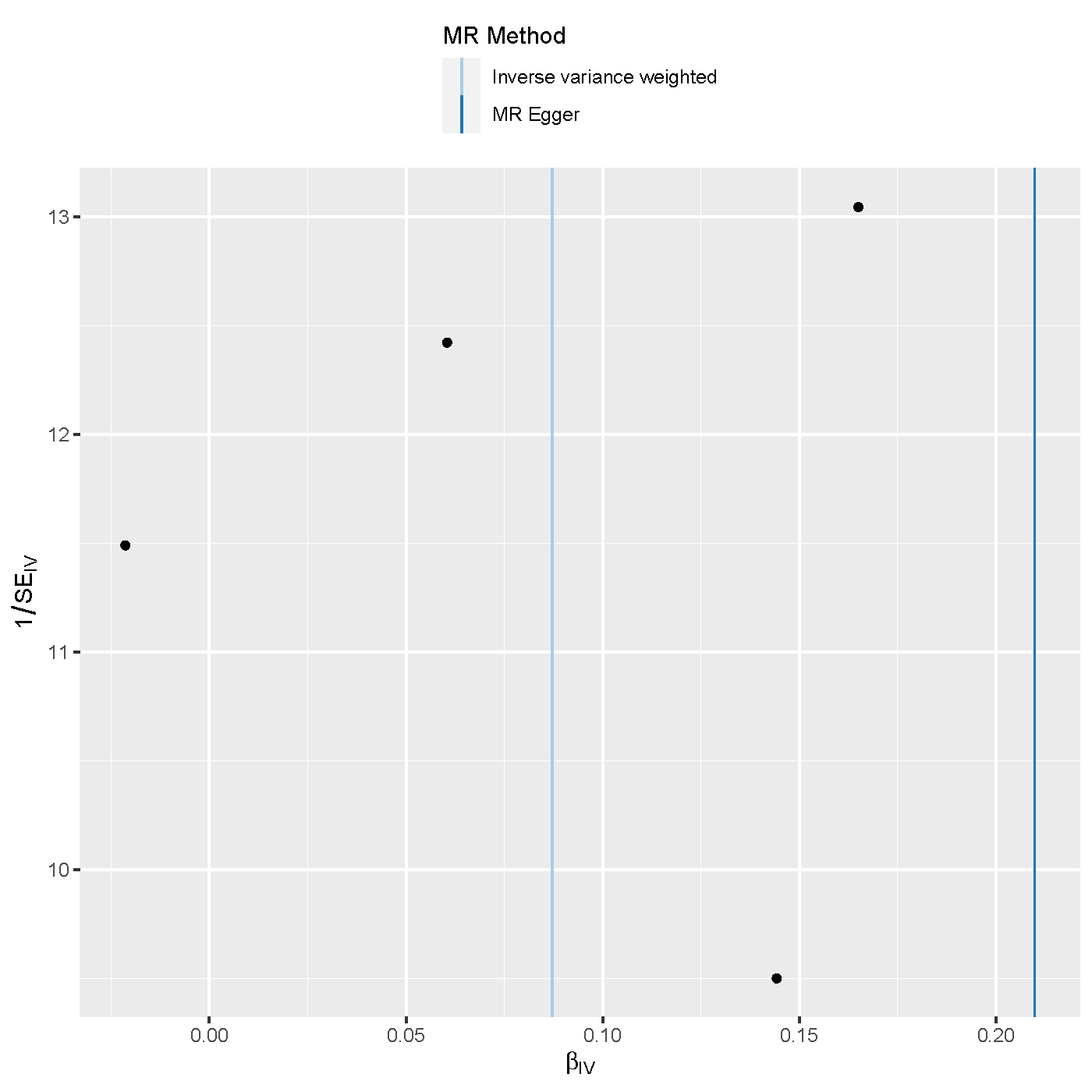


**C D**


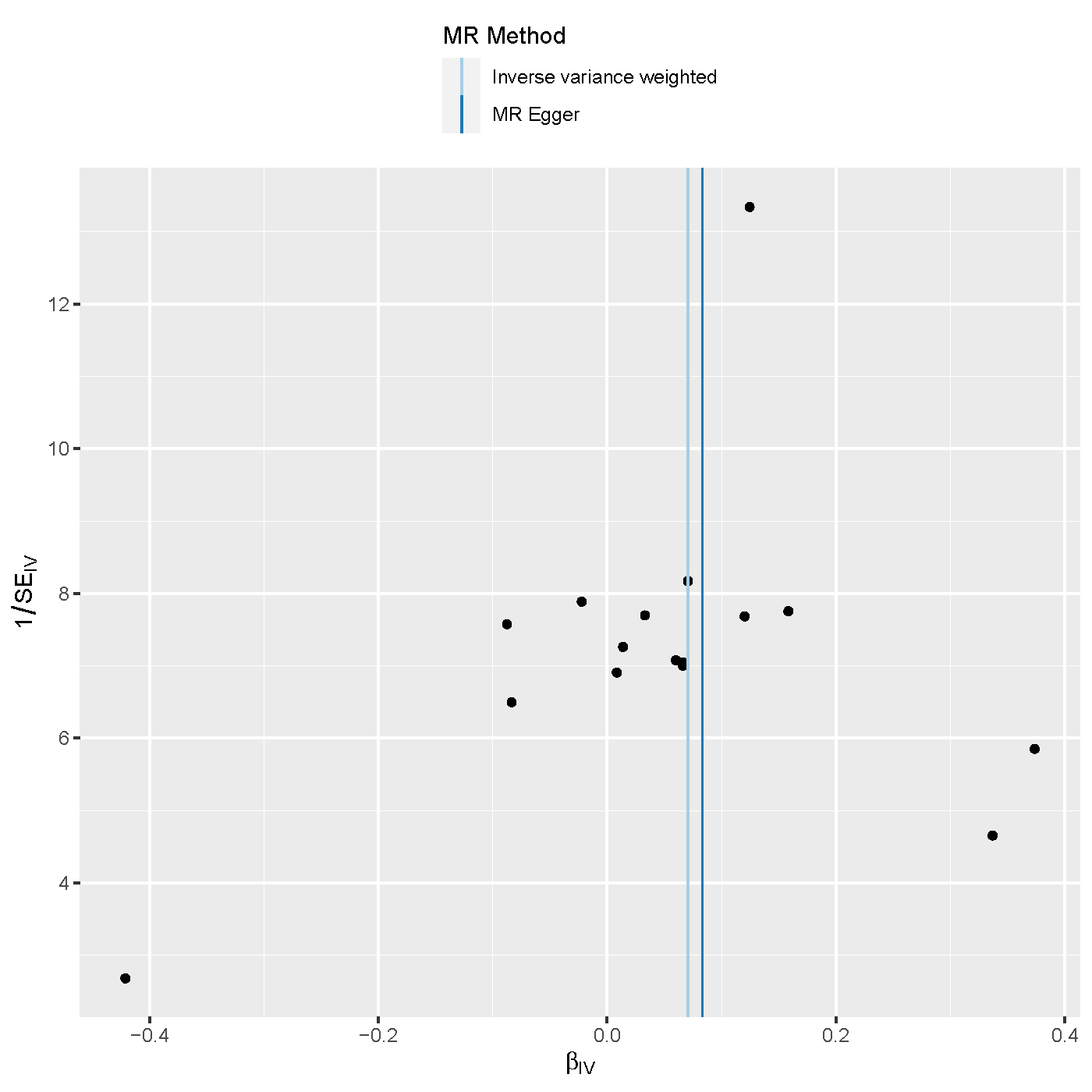

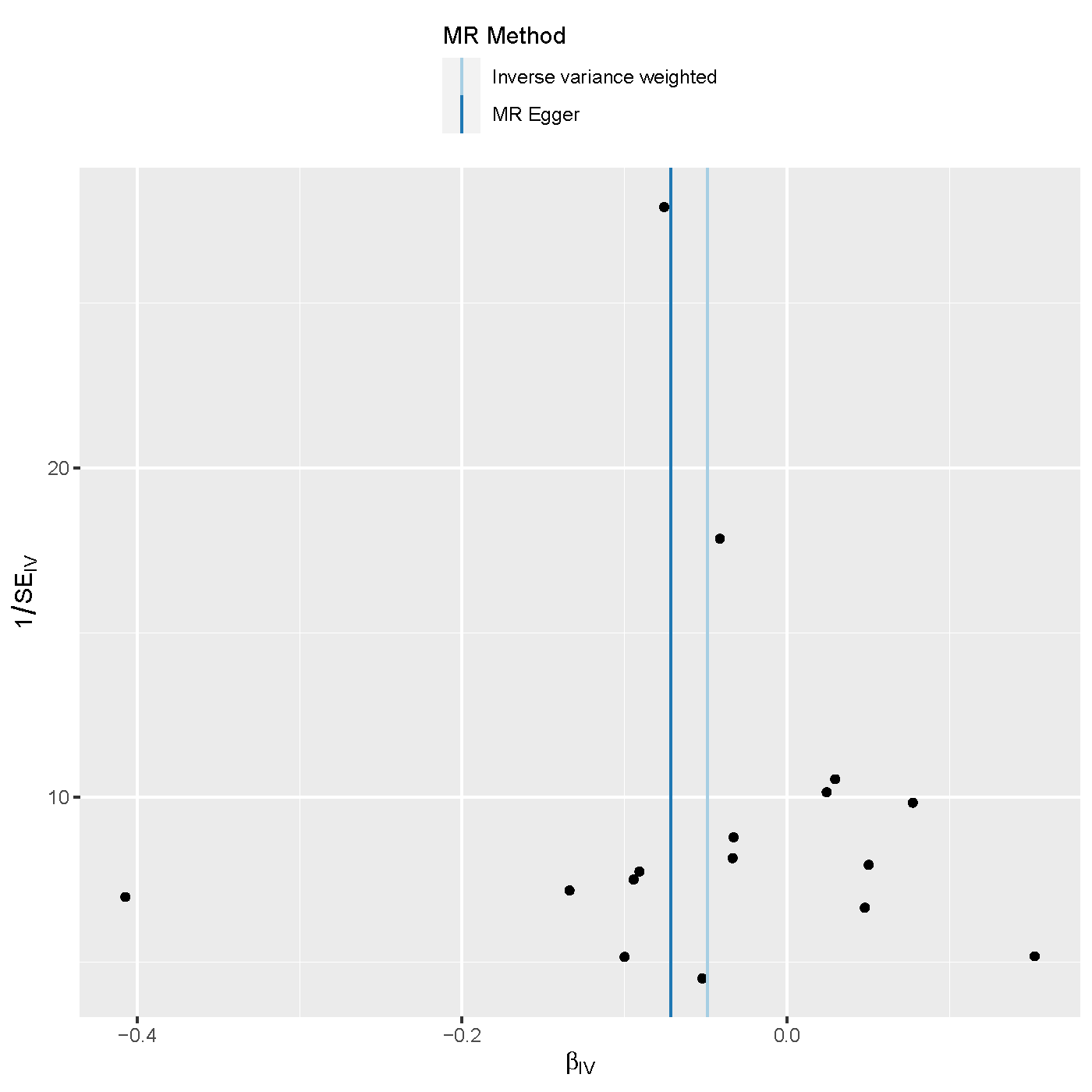


**E**


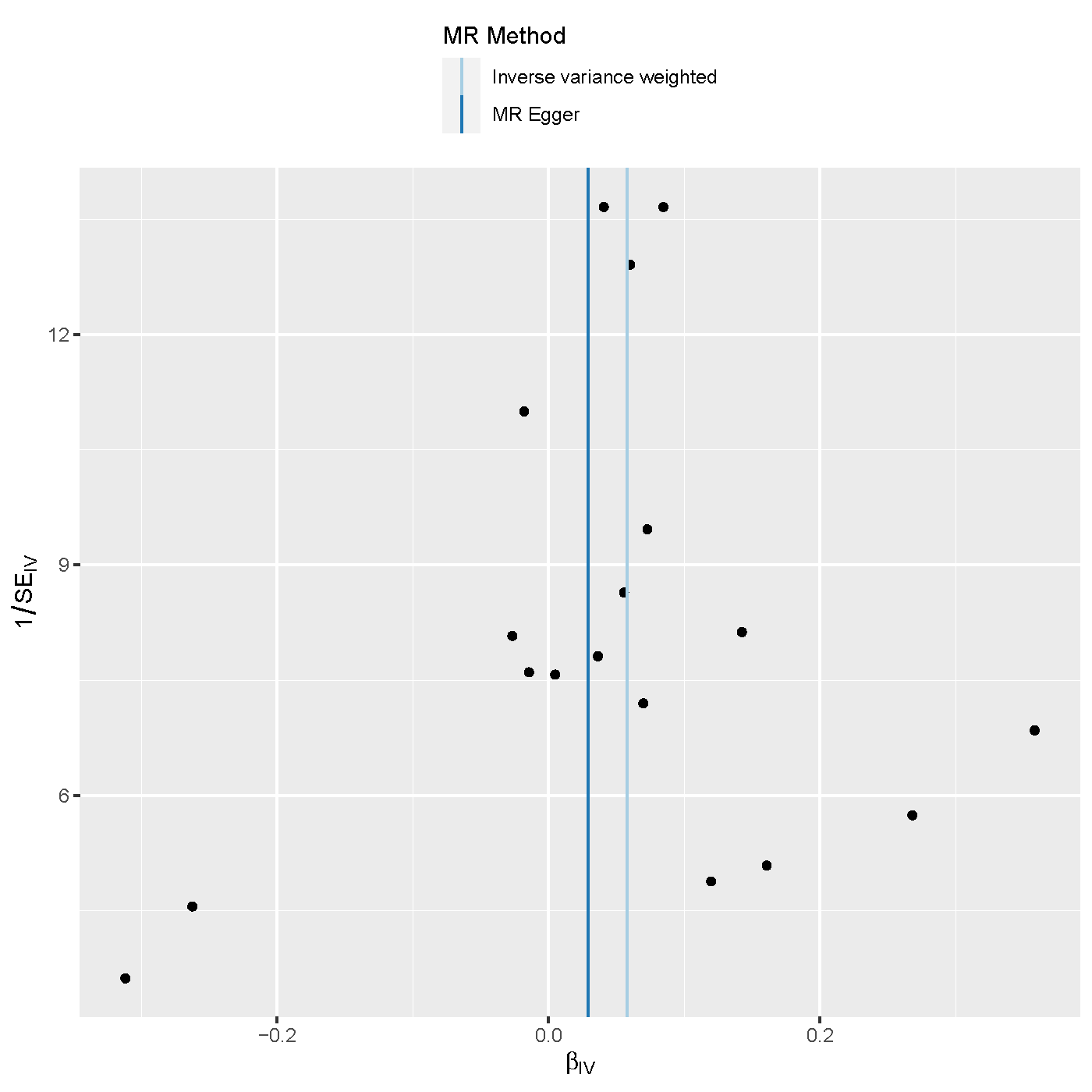


**Supplementary Figure 6.** (A) Funnel plots for the exposure of IL-1β (B) Funnel plots for the exposure of IL-4 (C) Funnel plots for the exposure of IL-12p70(D) Funnel plots for the exposure of TRAIL(E) Funnel plots for the exposure of VEGF

**A B**


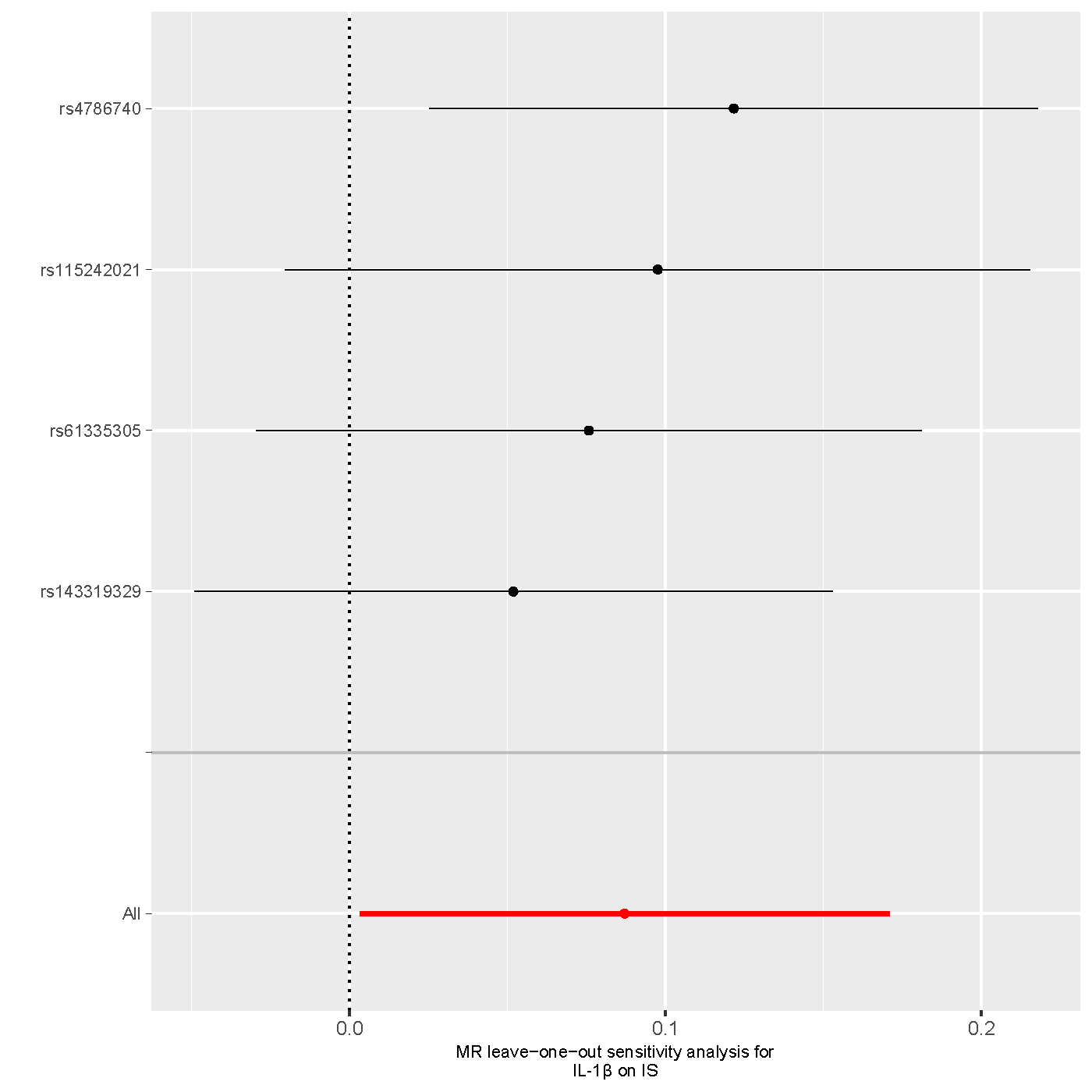


**C D**


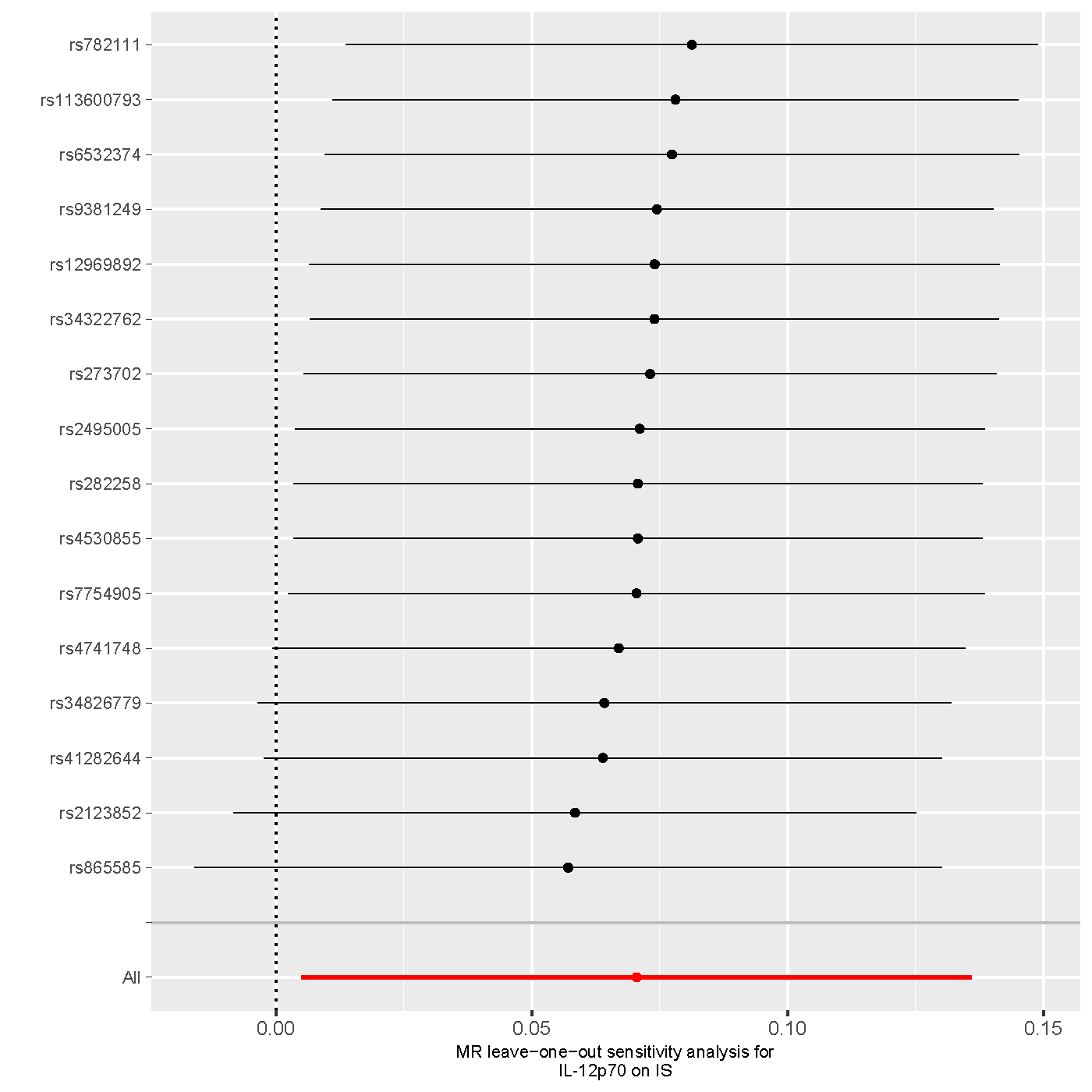

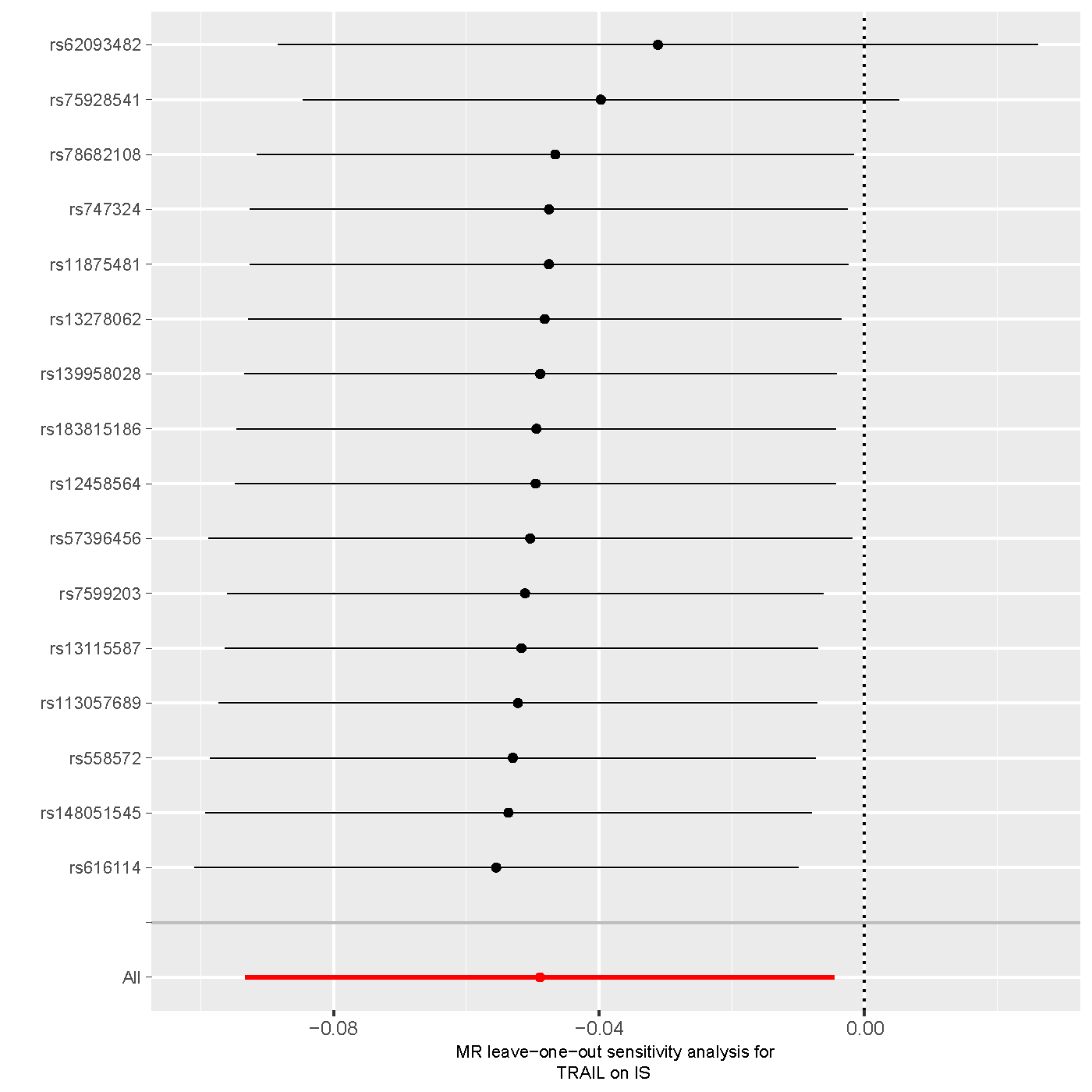


**E**


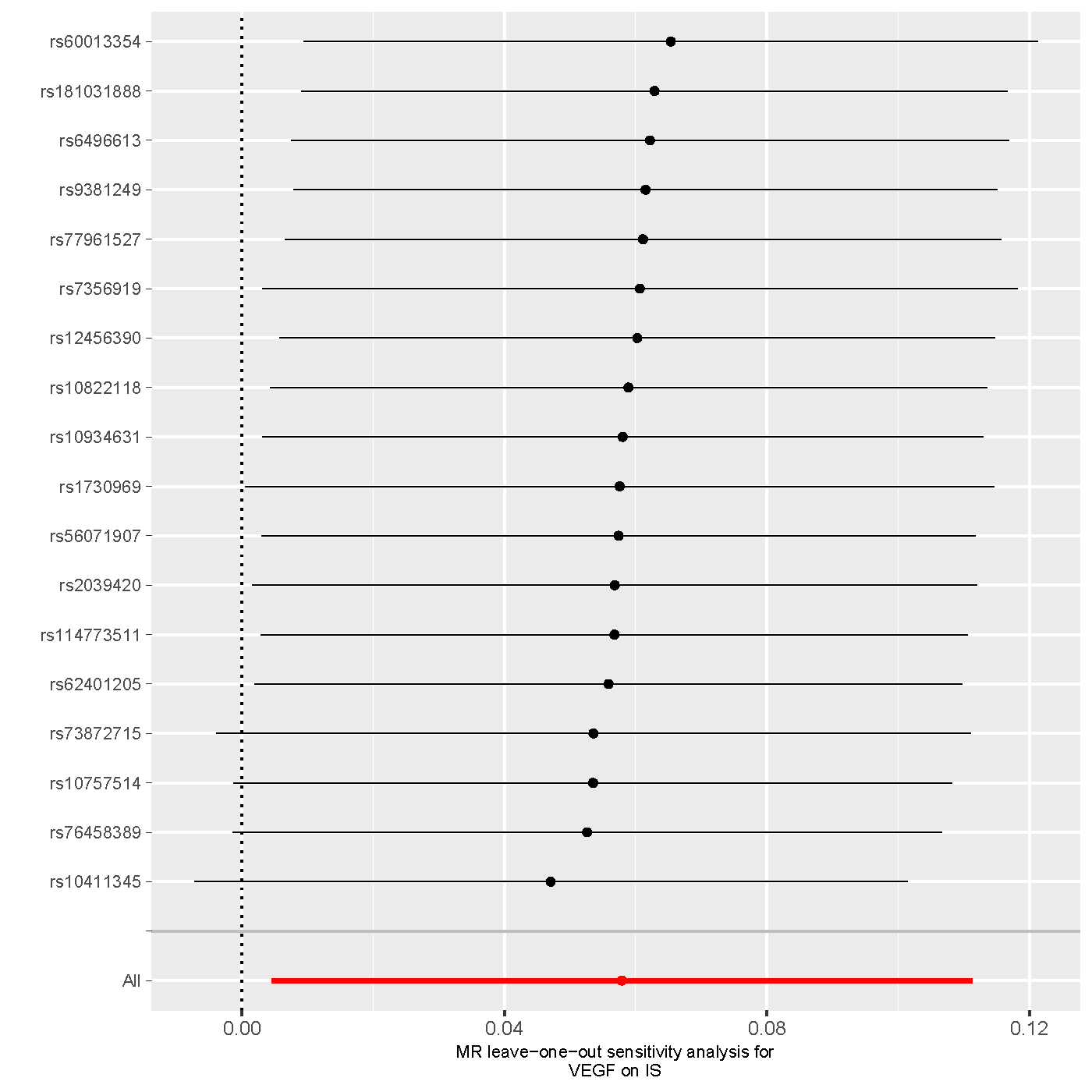


**Supplementary Figure 7.** (A) Leave-one-out plots for the exposure of IL-1β (B) Leave-one-out plots for the exposure of IL-4 (C) Leave-one-out plots for the exposure of IL-12p70(D) Leave-one-out plots for the exposure of TRAIL(E) Leave-one-out plots for the exposure of VEGF

**A B**


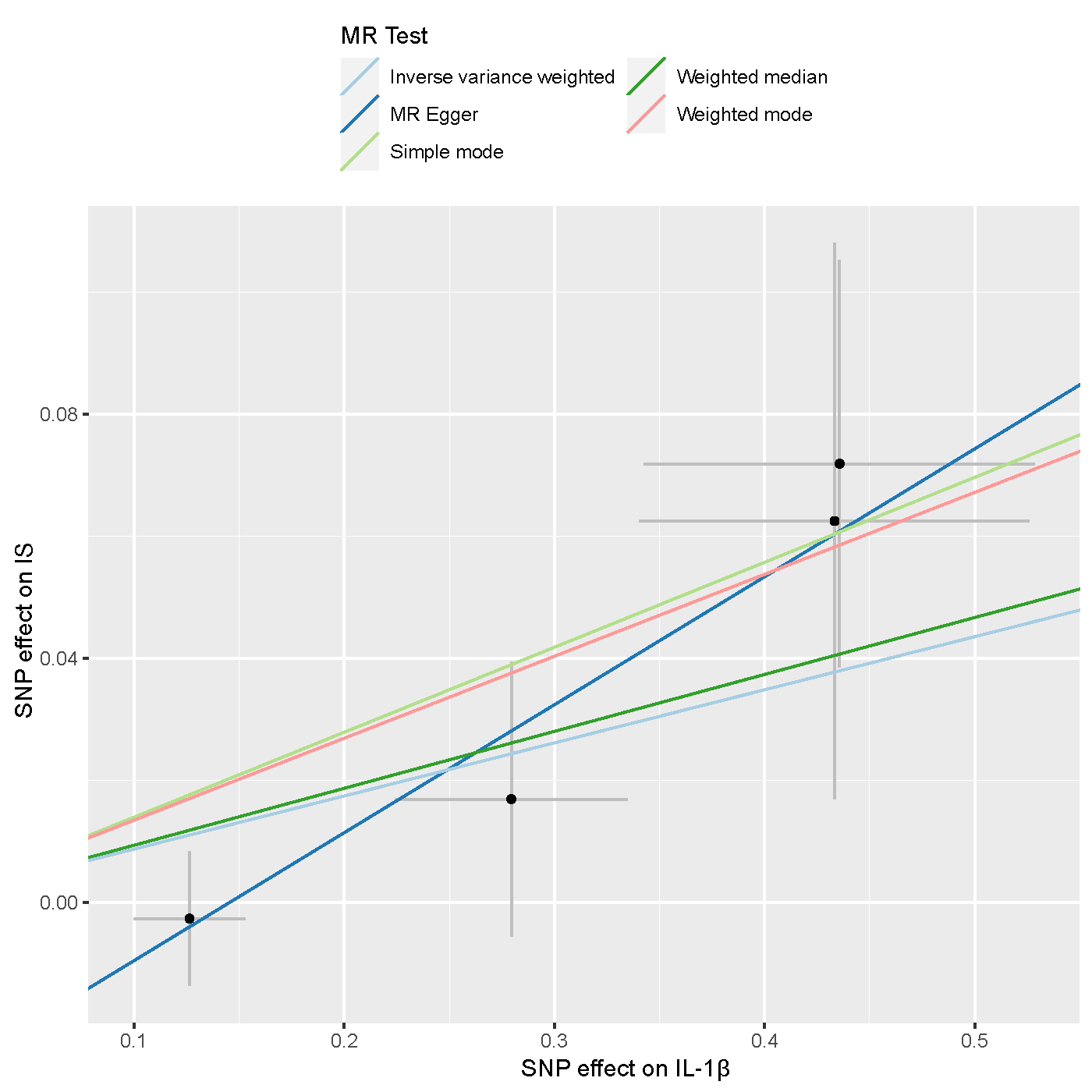


**C D**


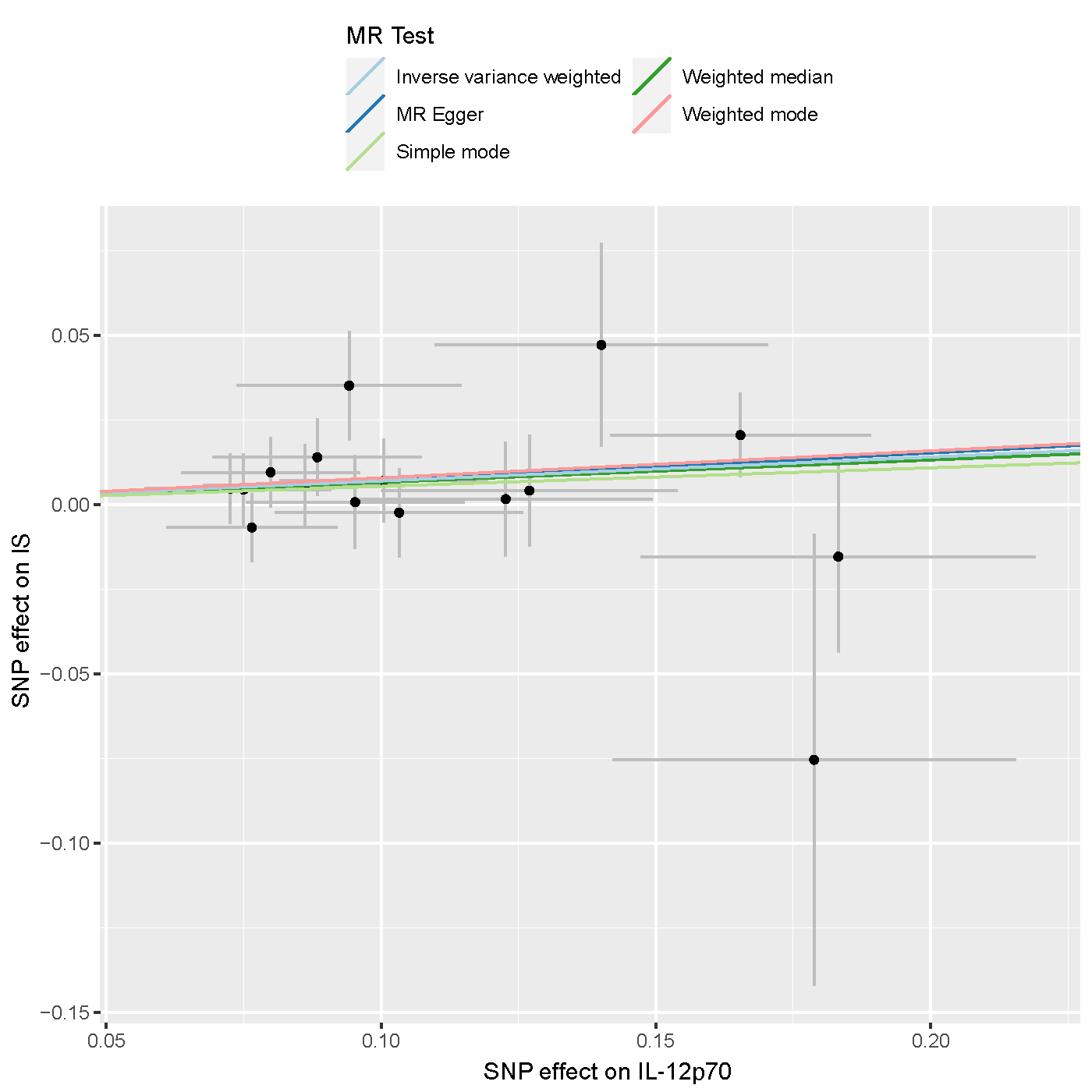

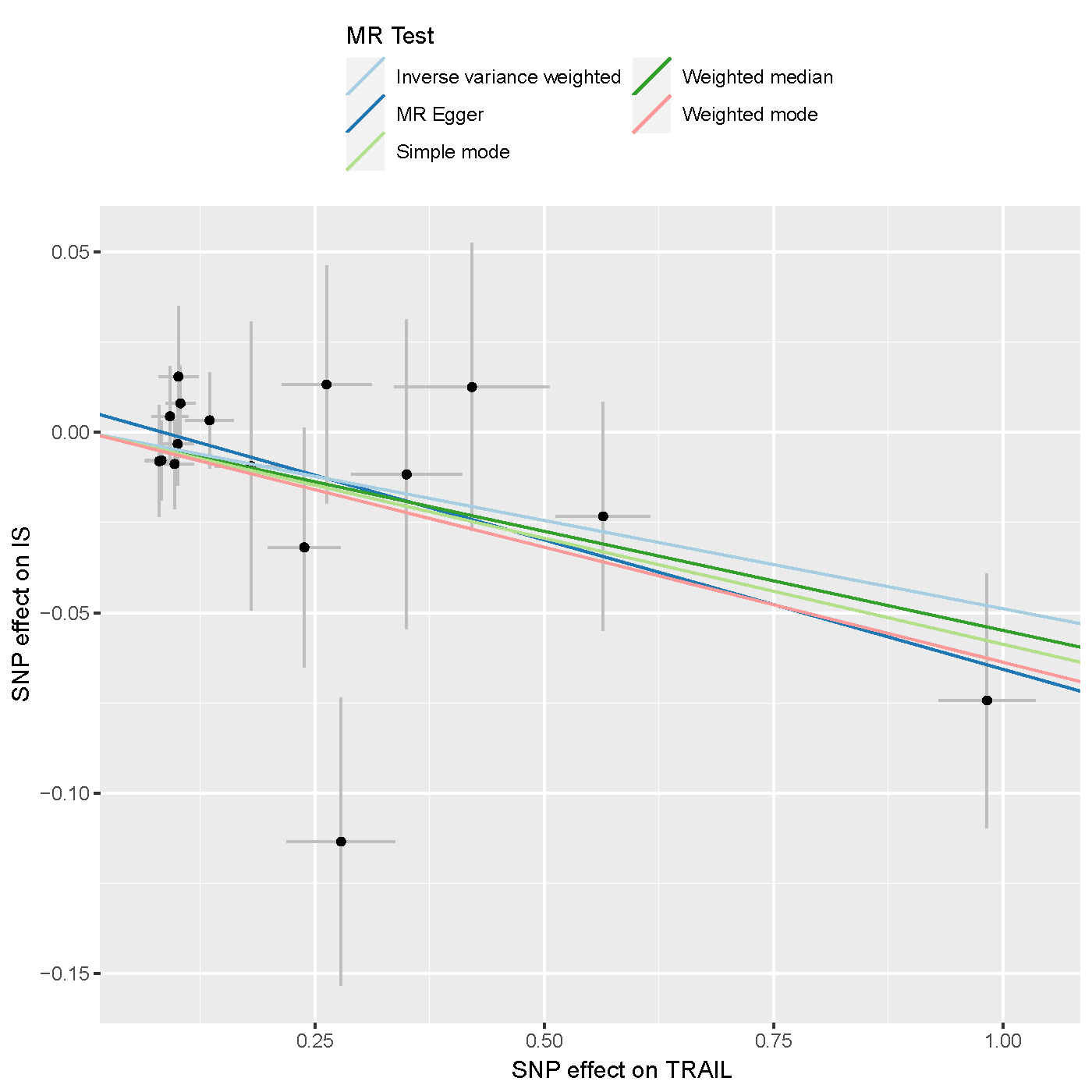


**E**


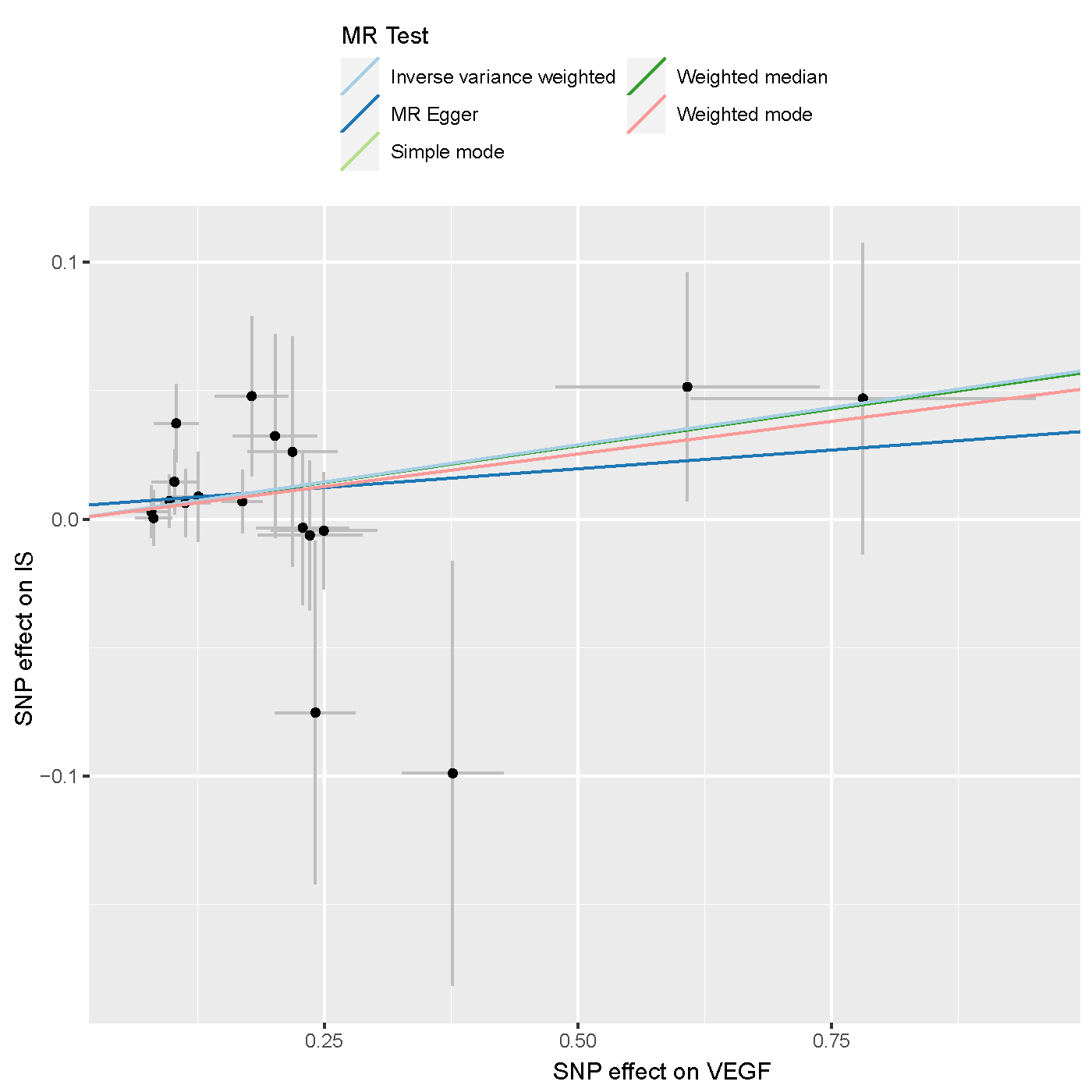


**Supplementary Figure 13.** (A) Scatter plots for the exposure of IL-1β (B) Scatter plots for the exposure of IL-4 (C) Scatter plots for the exposure of IL-12p70(D) Scatter plots for the exposure of TRAIL(E) Scatter plots for the exposure of VEGF

**A B**


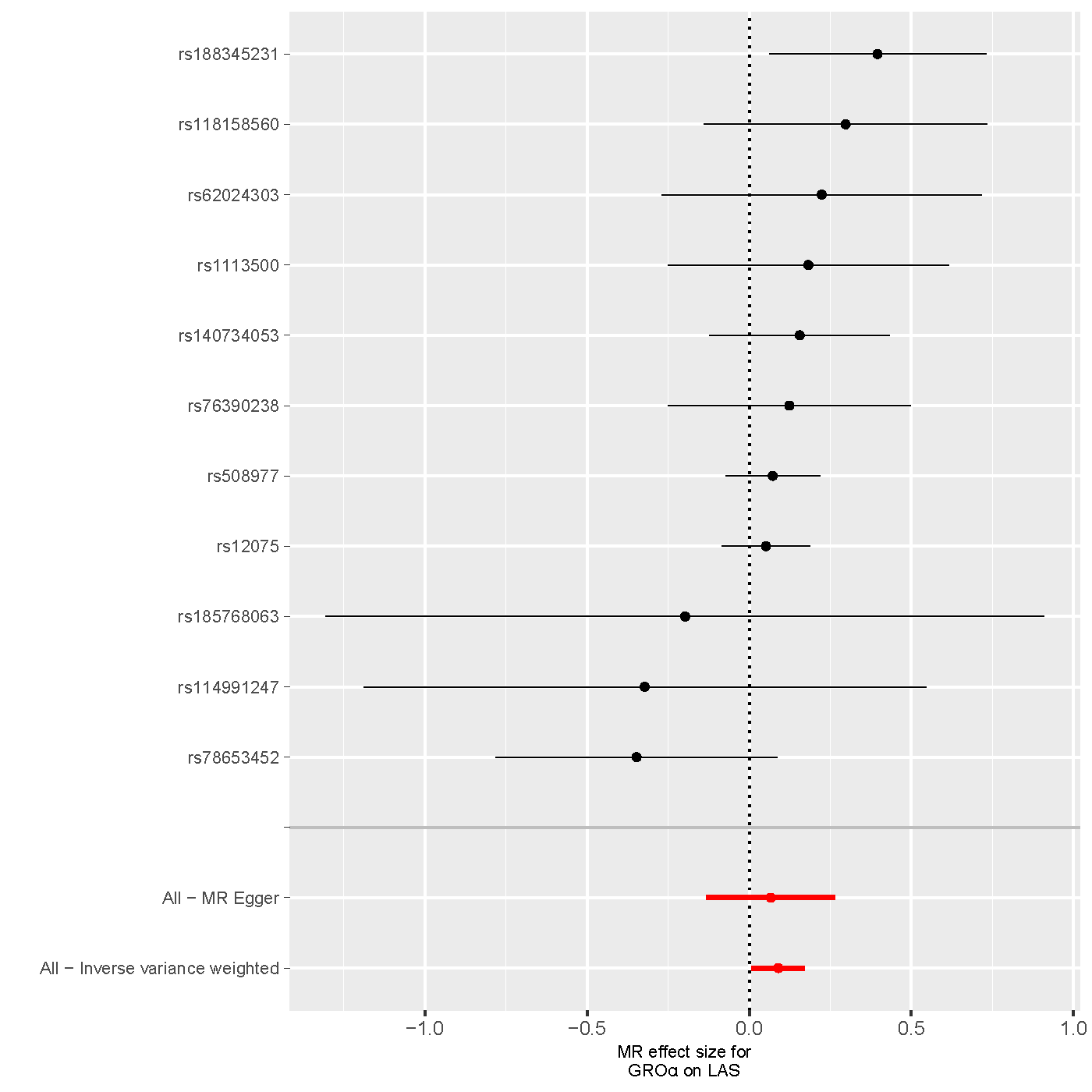

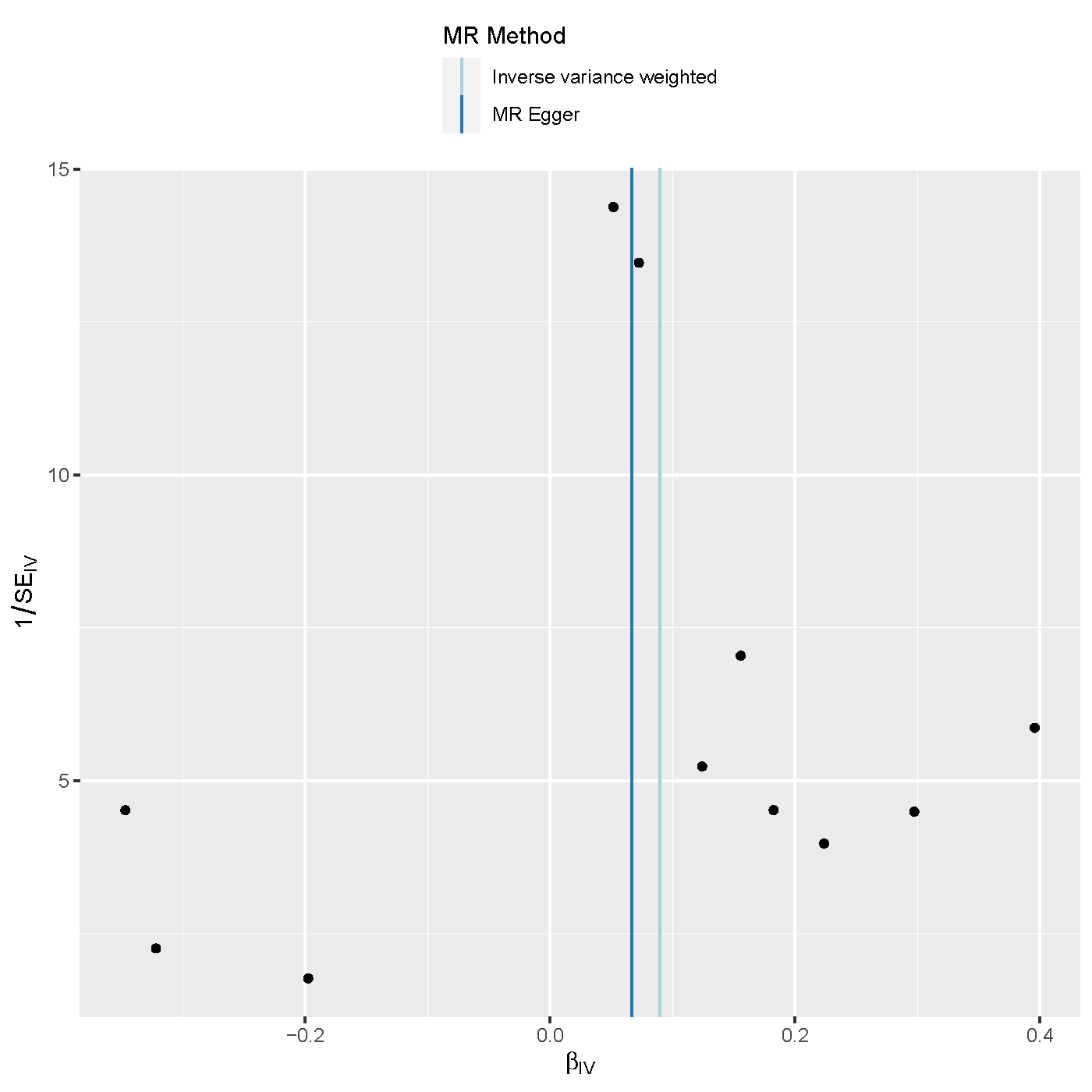


**C D**

**Supplementary Figure 9.** (A) Forest plots for the exposure of GROα (B) Funnel plots for the exposure of GROα (C) Leave-one-out plots for the exposure of GROα (D)Scatter plots for the exposure of GROα

**A B**


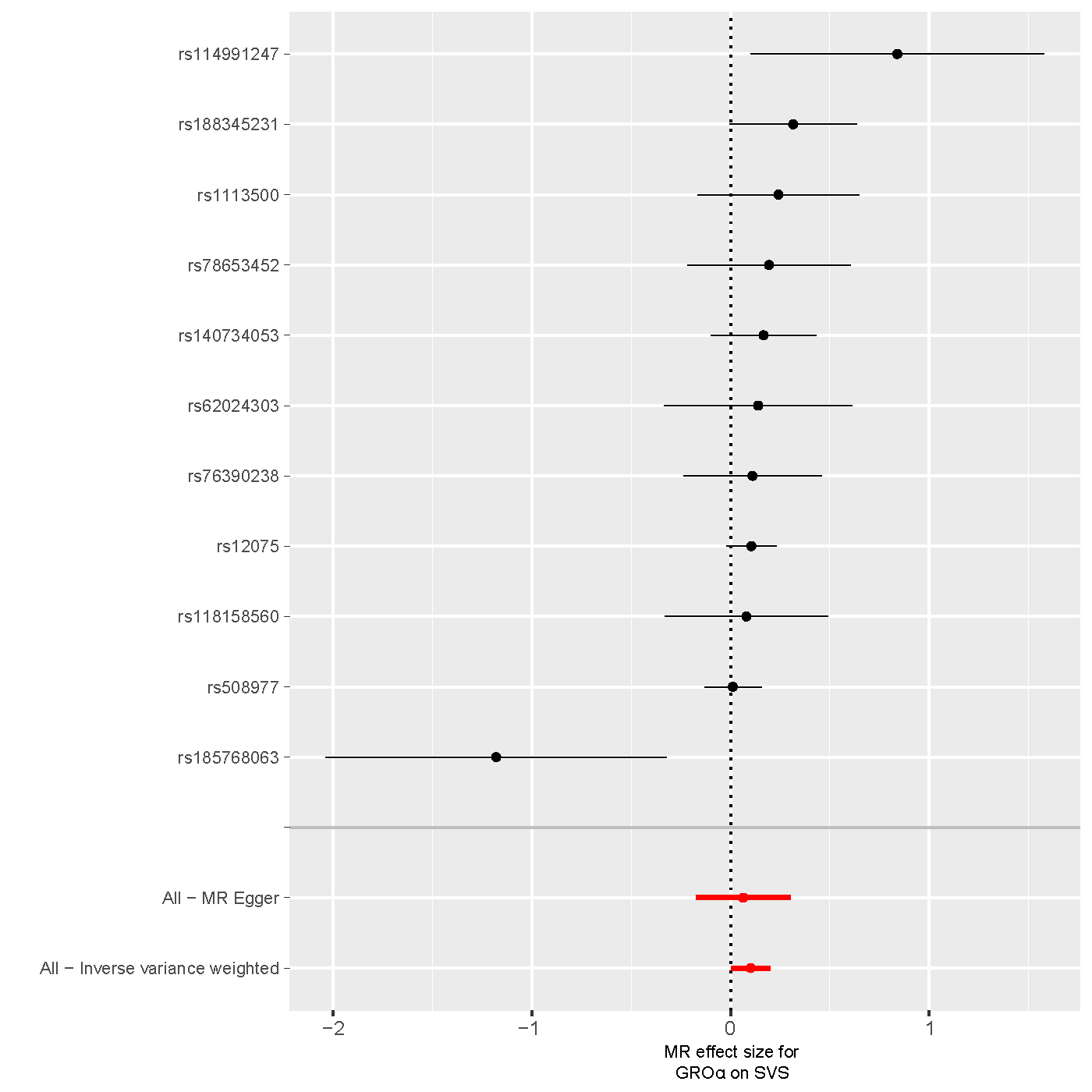


**C D**


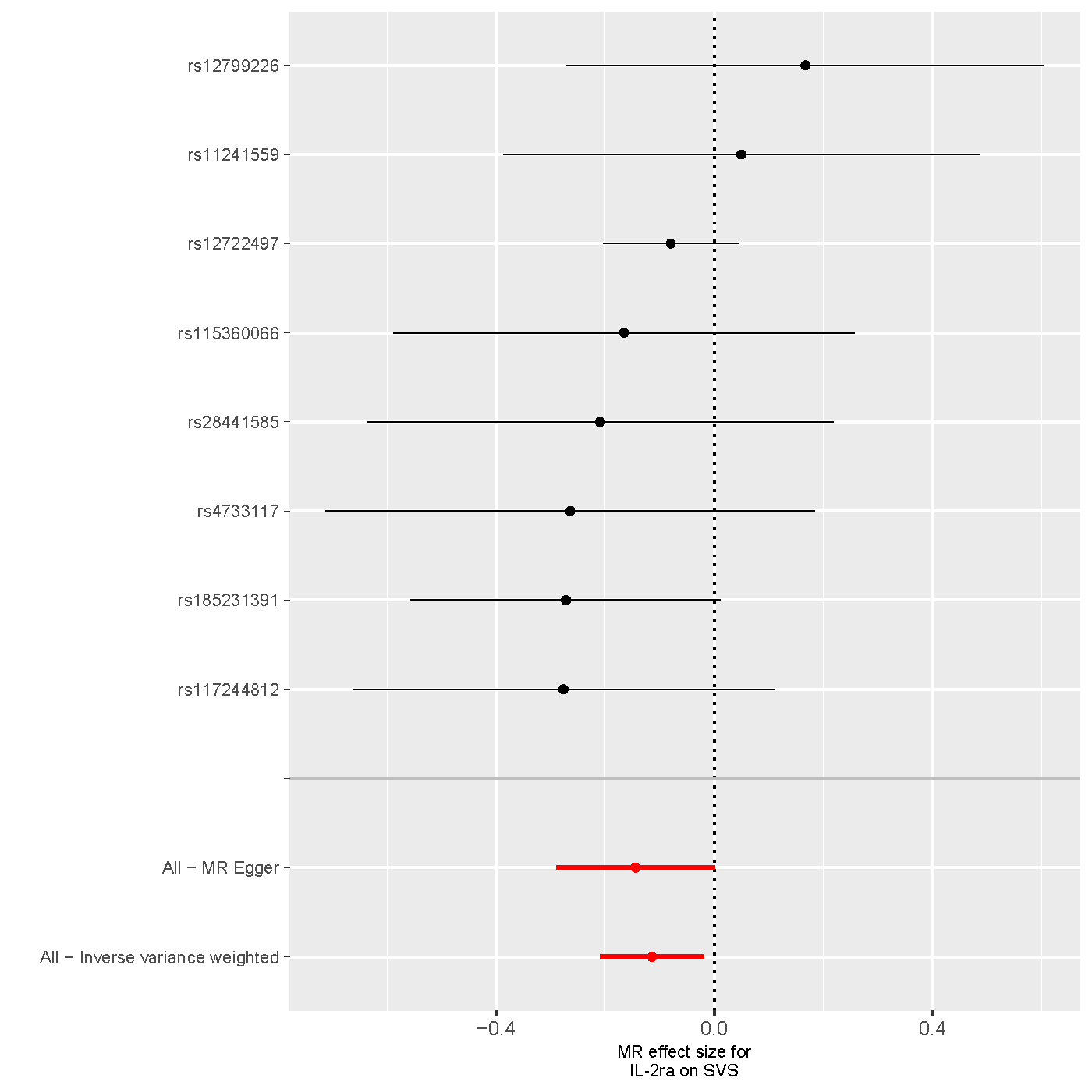

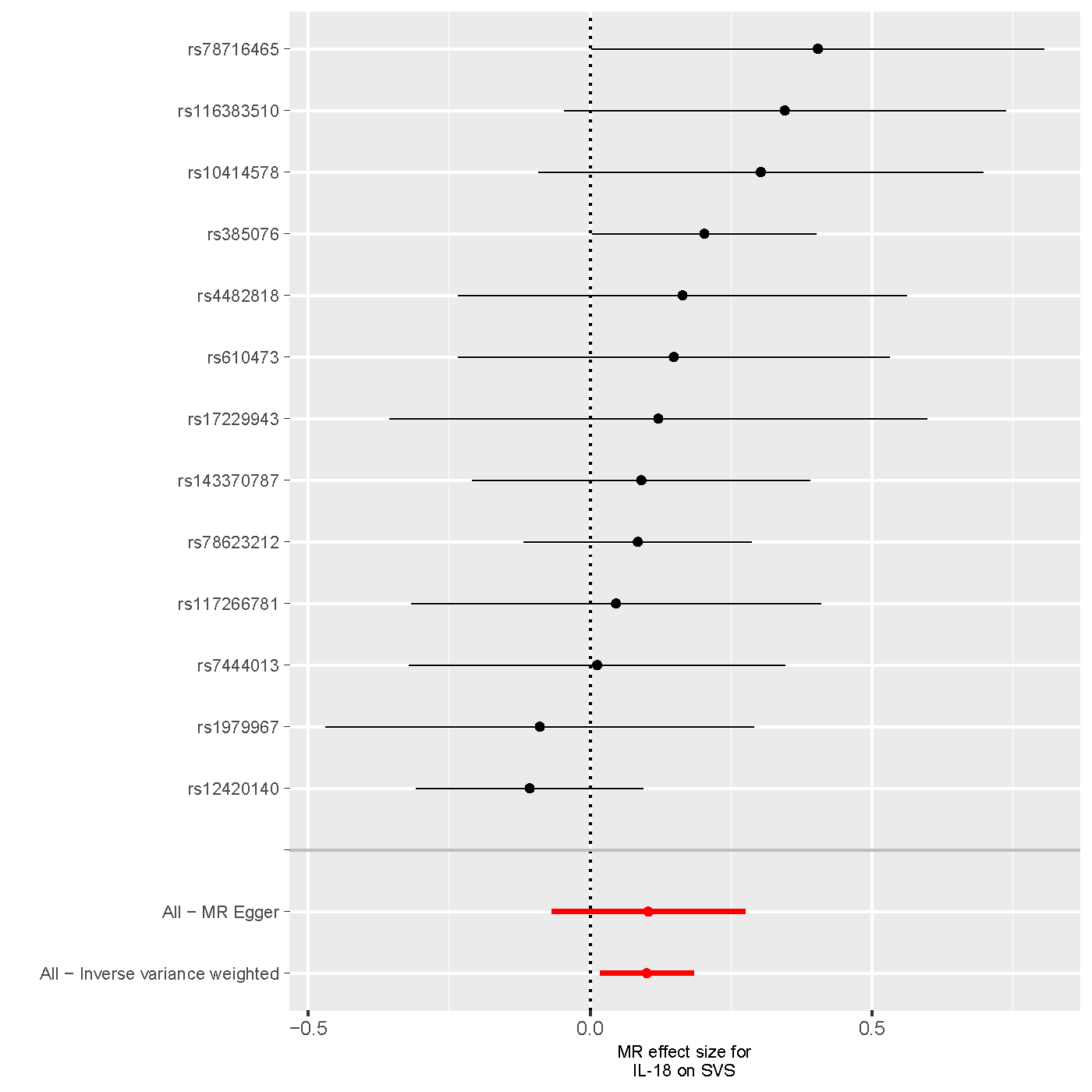


**E**


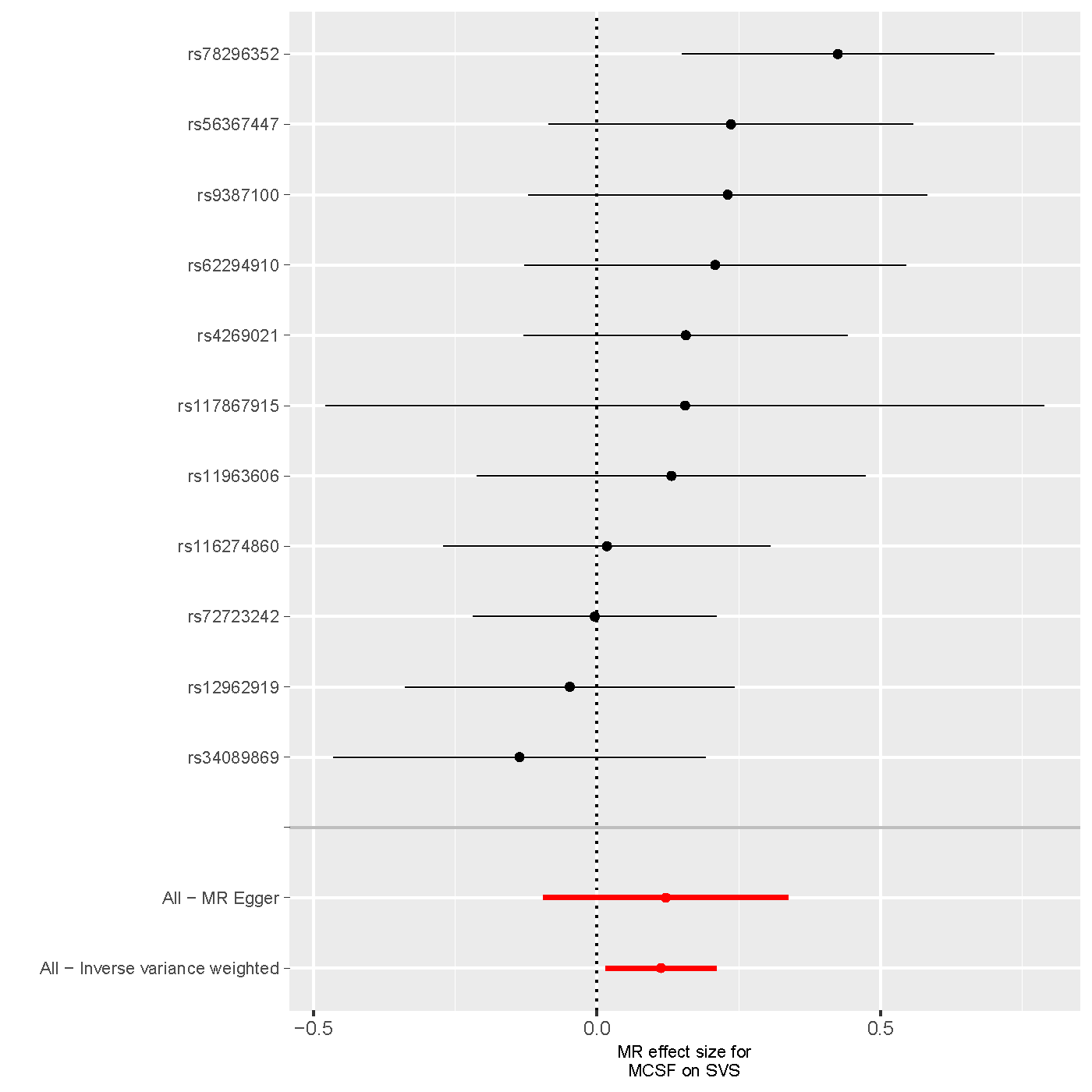


**Supplementary Figure 10.** (A)Forest plots for the exposure of β-NGF (B) Forest plots for the exposure of GROα (C) Forest plots for the exposure of IL-2RA(D) Forest plots for the exposure of IL-18(E) Forest plots for the exposure of MCSF

**A B**


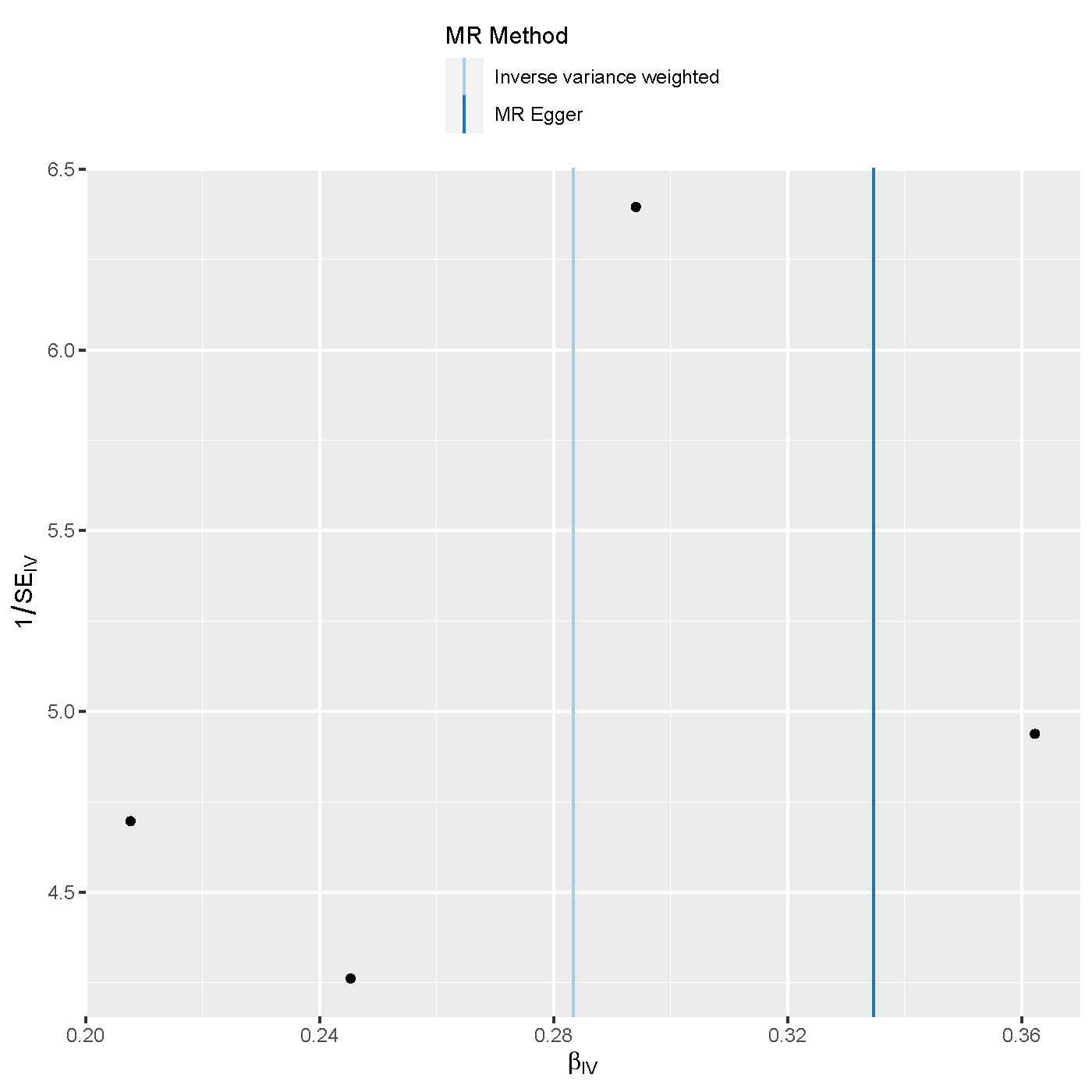

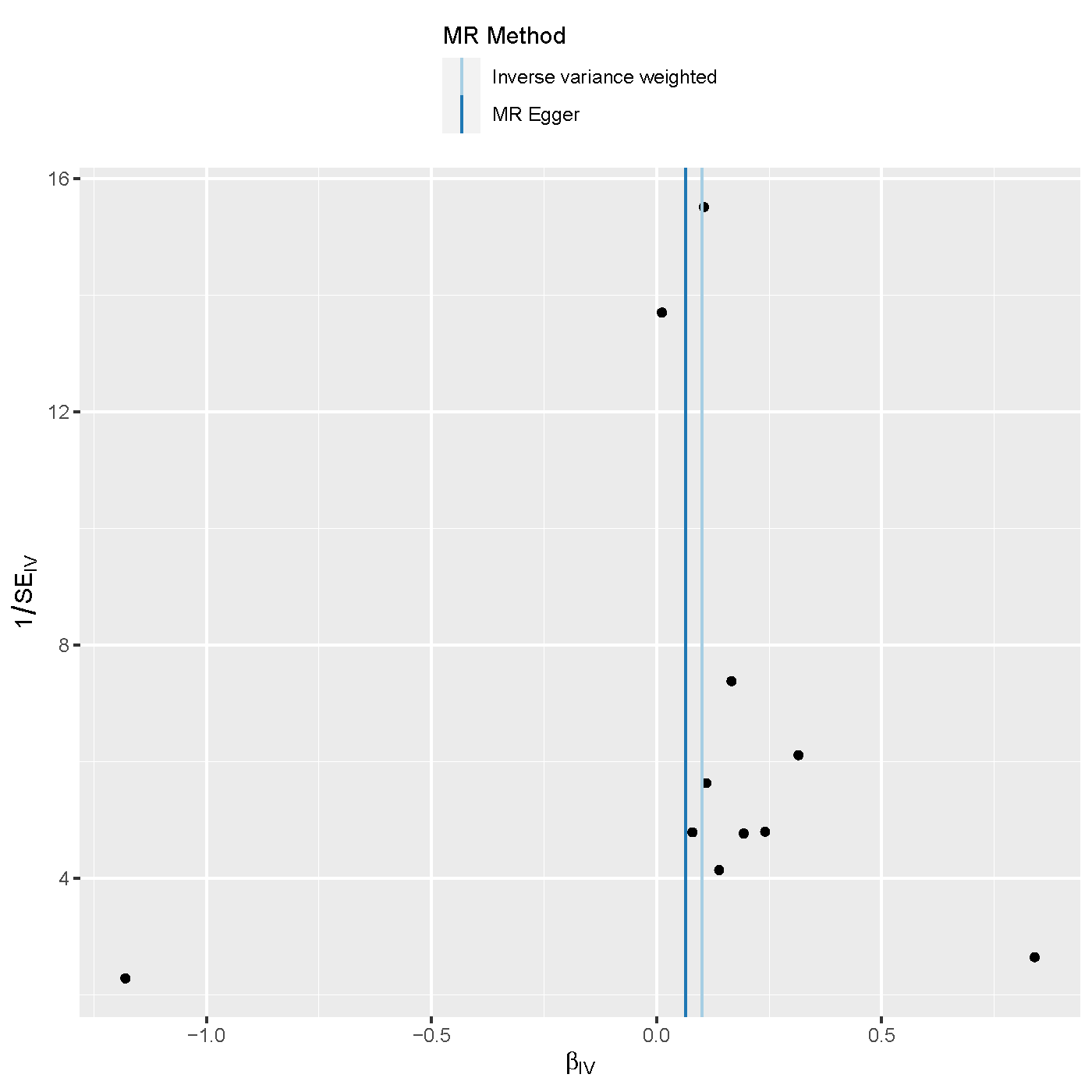


**C D**


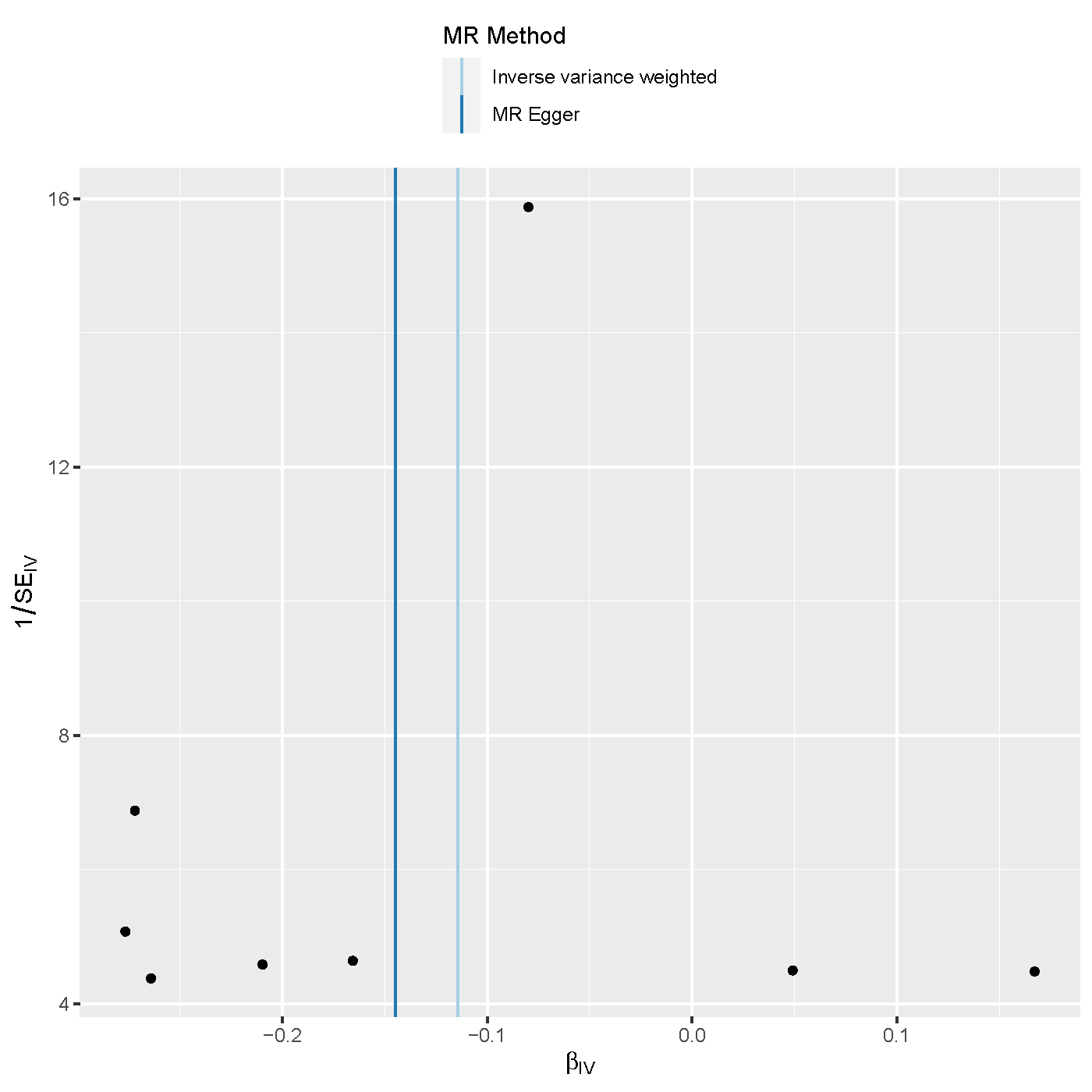

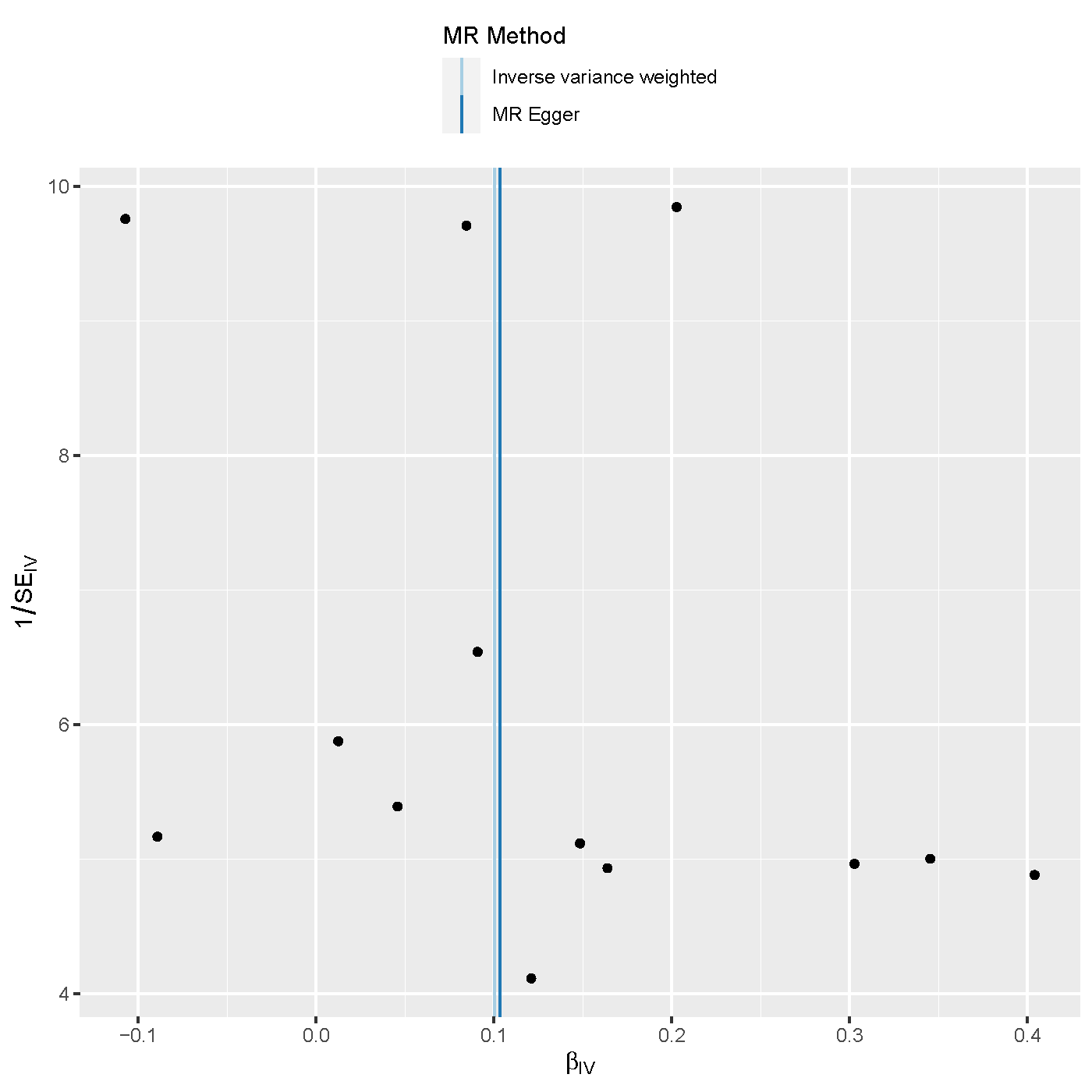
**E**


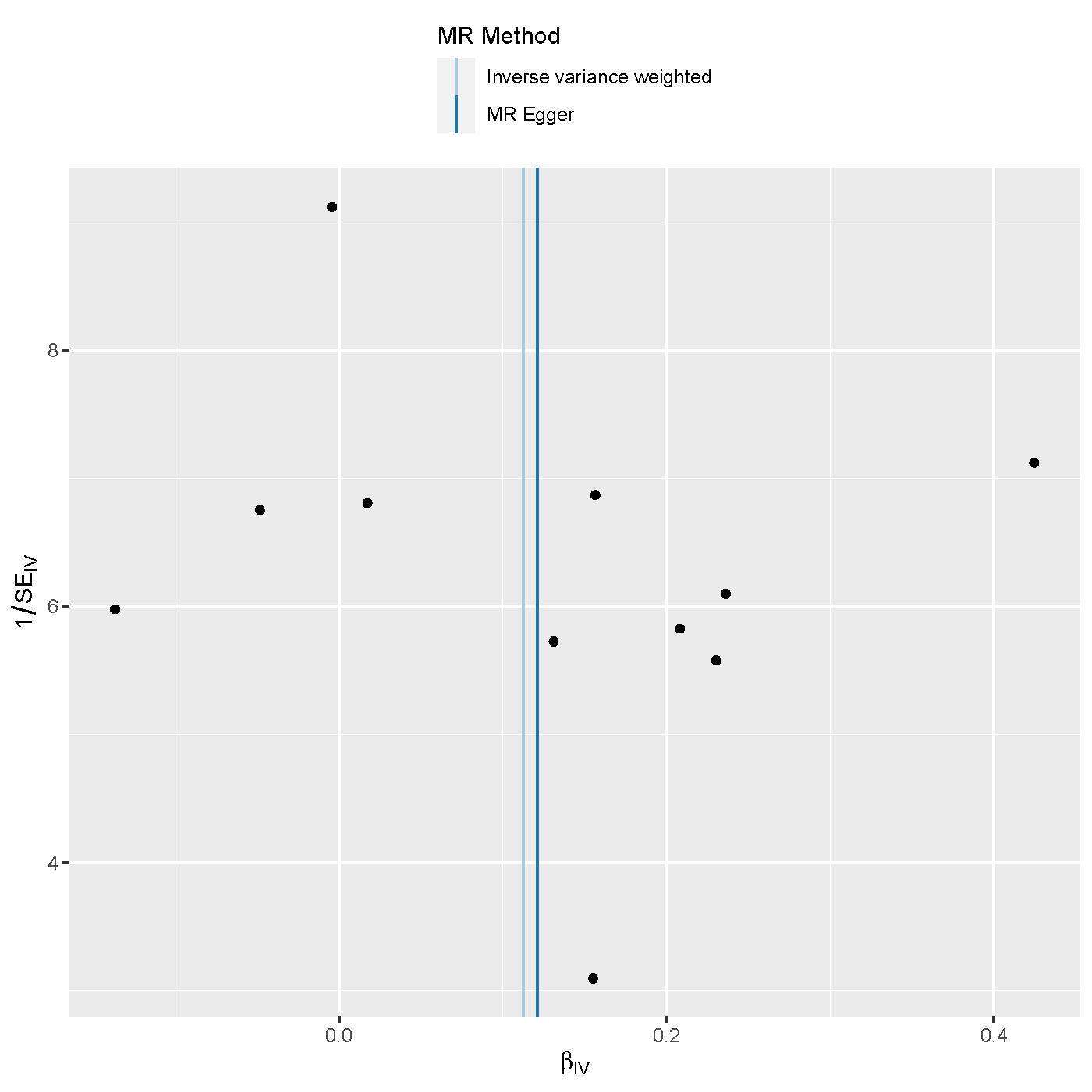


**Supplementary Figure 11.** (A) Funnel plots for the exposure of β-NGF (B) Funnel plots for the exposure of GROα (C) Funnel plots for the exposure of IL-2RA(D) Funnel plots for the exposure of IL-18(E) Funnel plots for the exposure of MCSF

**A B**


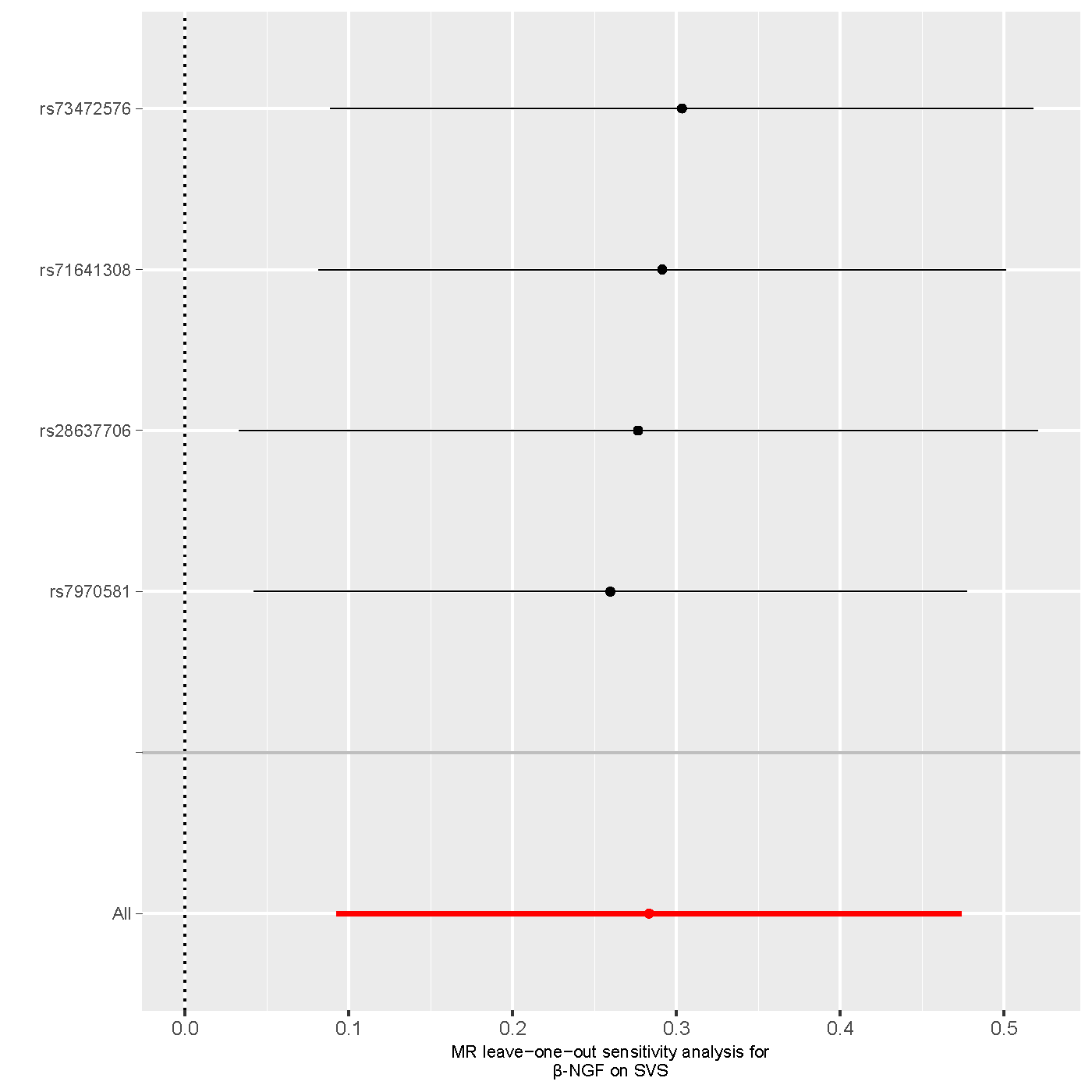

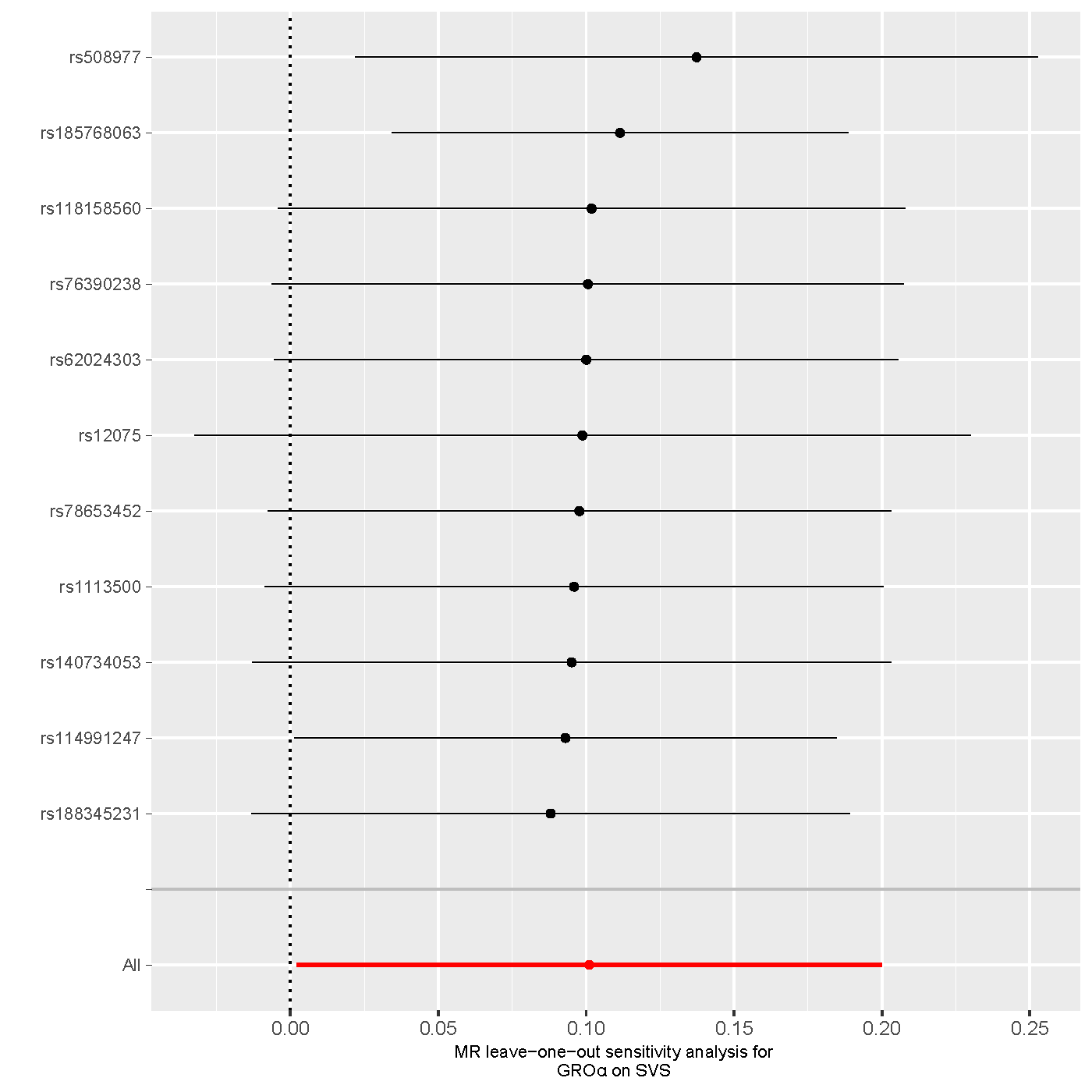


**C D**


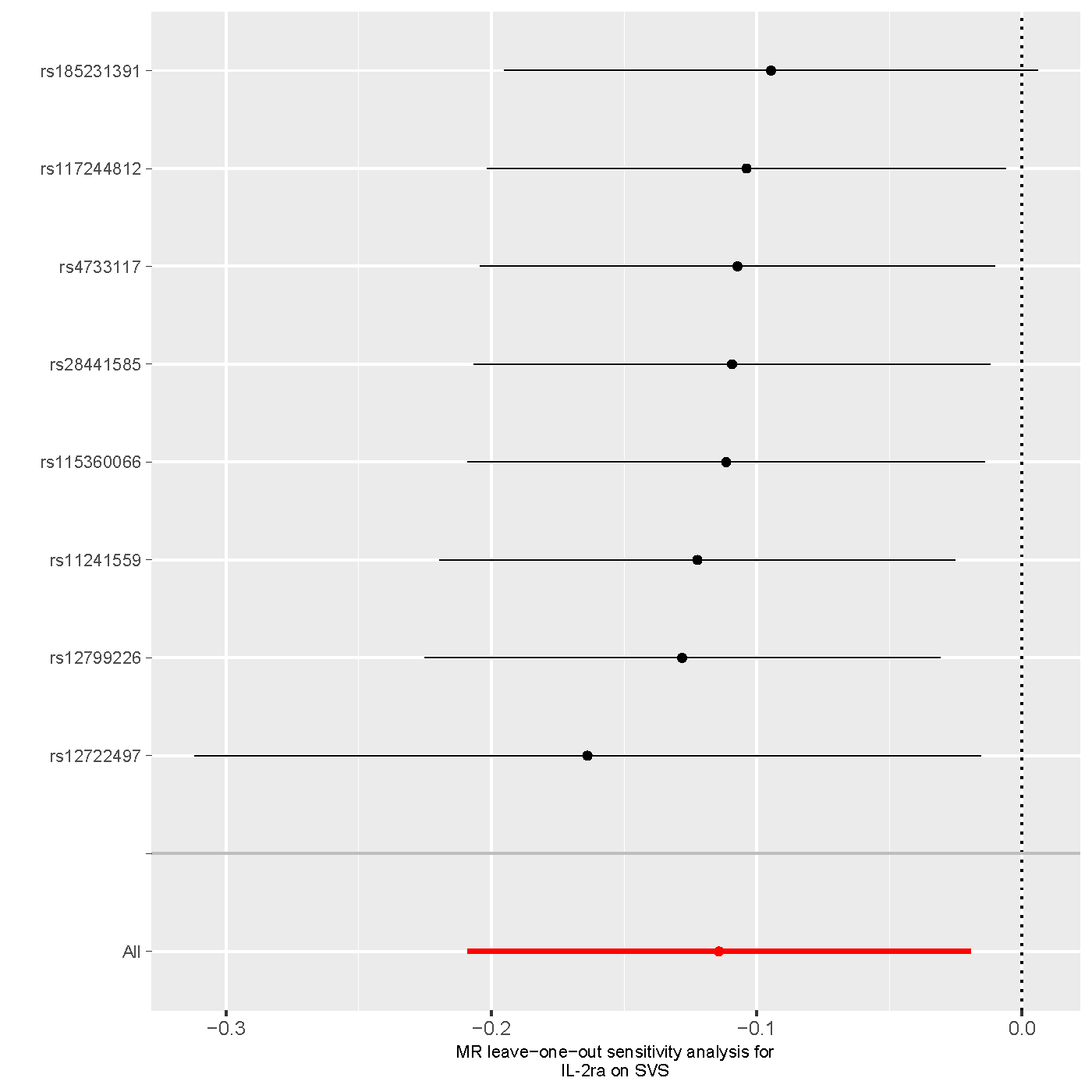

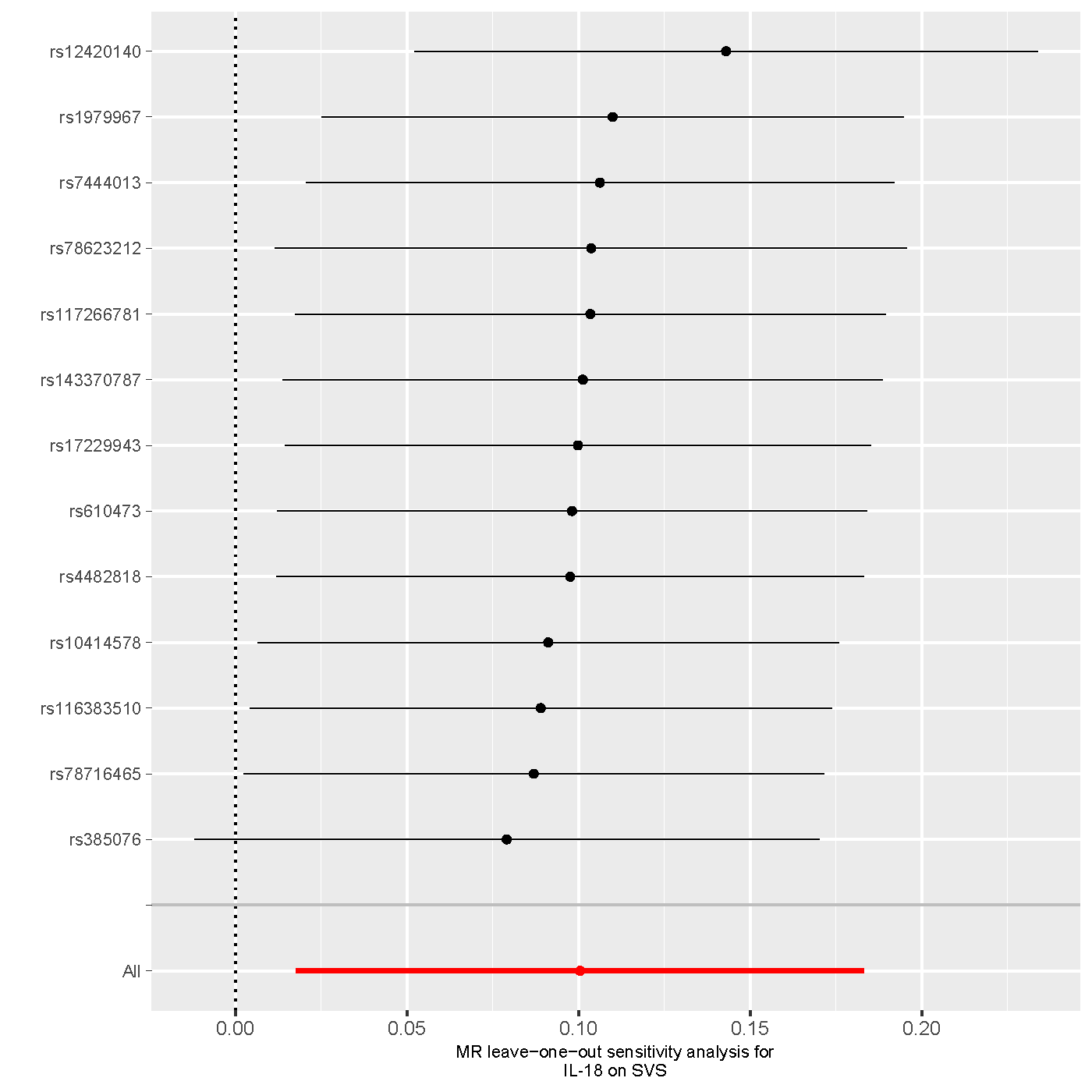


**E**


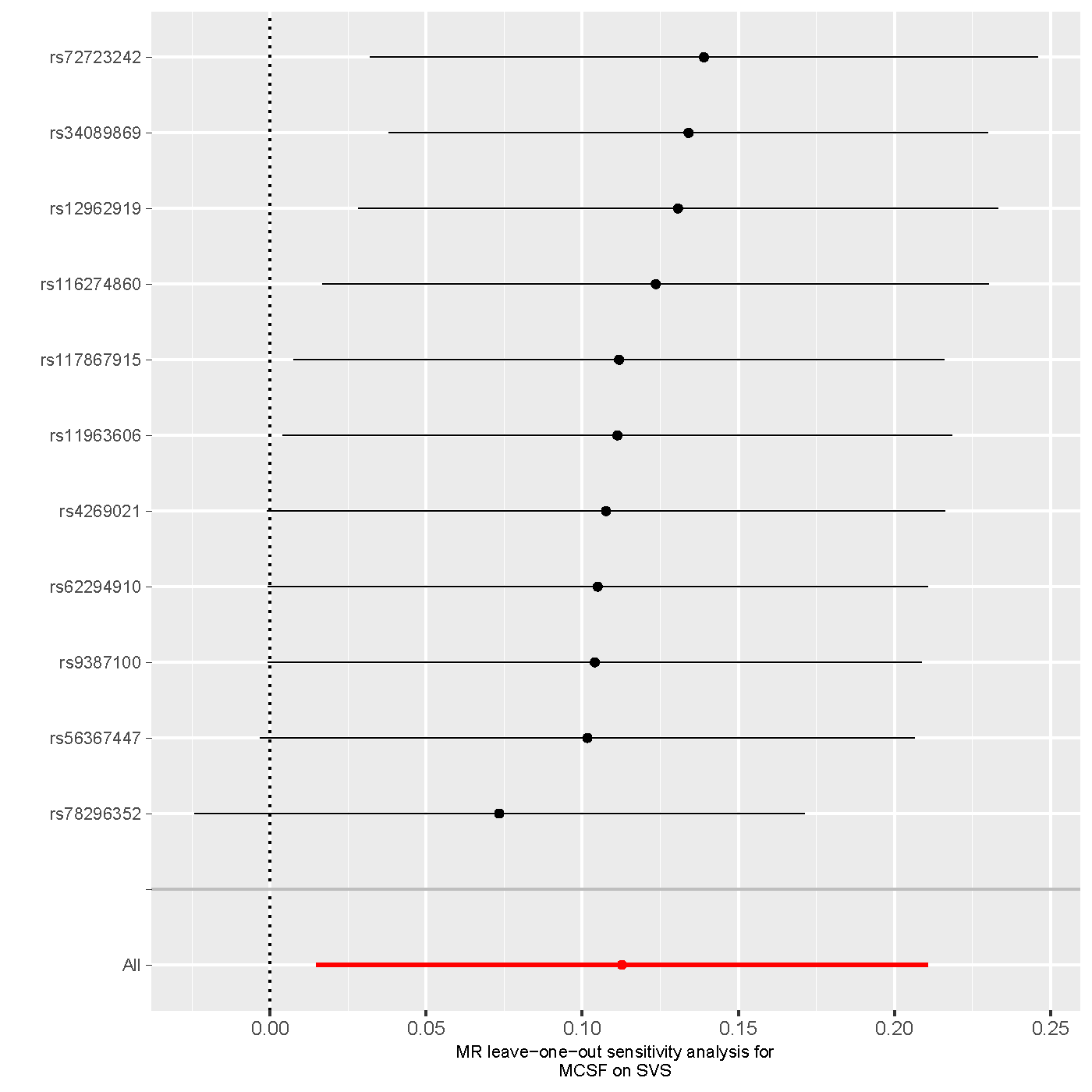


**Supplementary Figure 12.** (A) Leave-one-out plots for the exposure of β-NGF (B) Leave-one-out plots for the exposure of GROα (C) Leave-one-out plots for the exposure of IL-2RA(D) Leave-one-out plots for the exposure of IL-18(E) Leave-one-out plots for the exposure of MCSF

**A B**


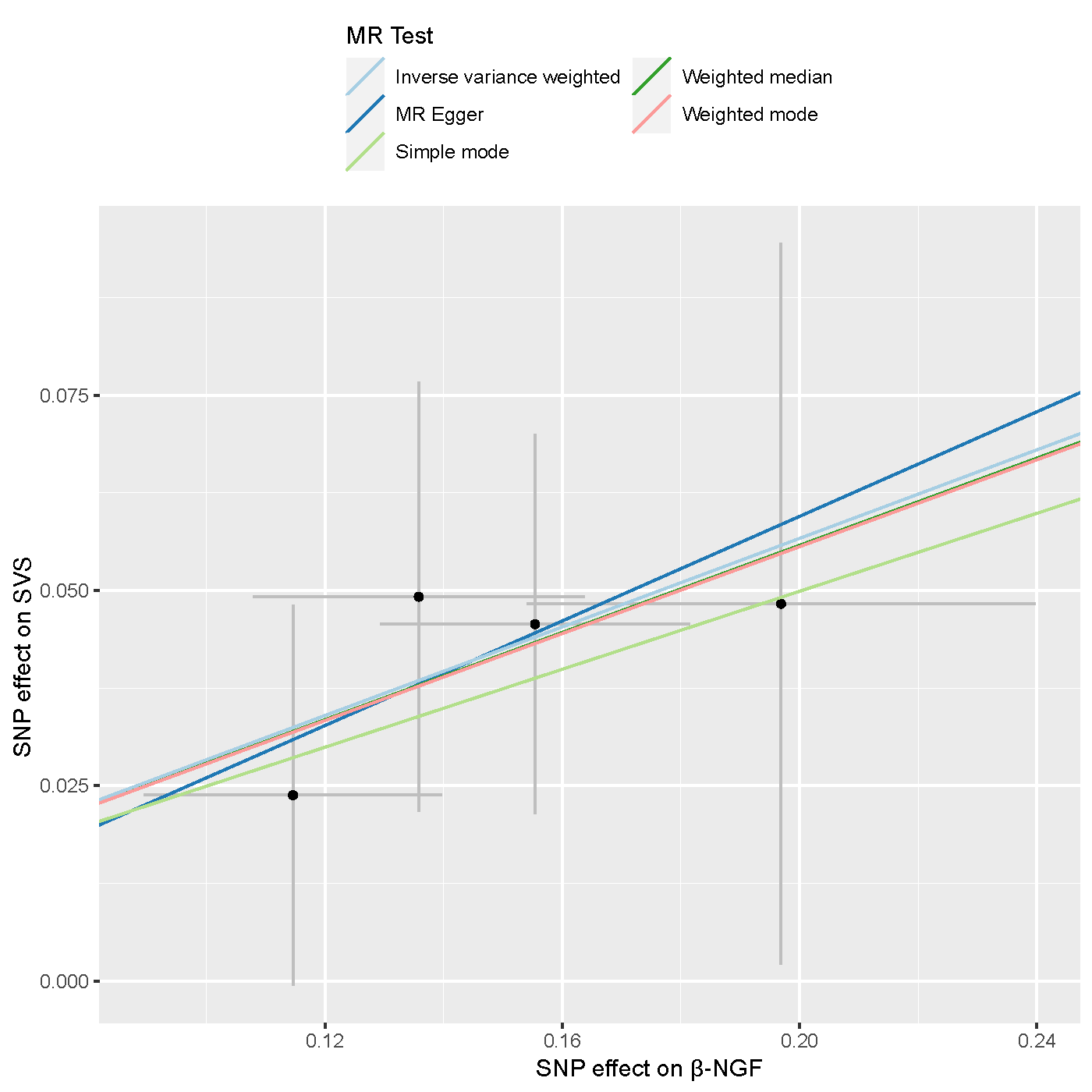

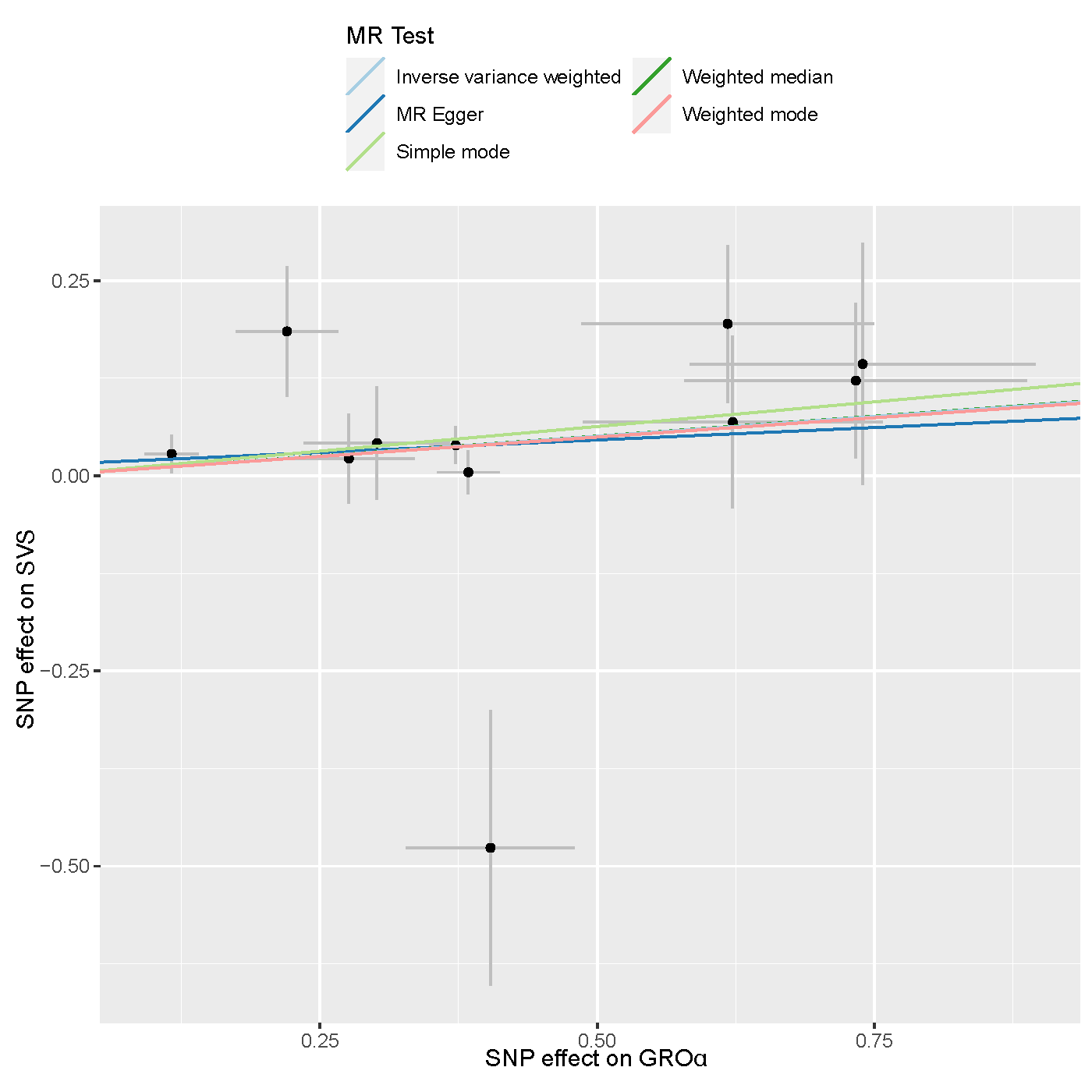


**C D**


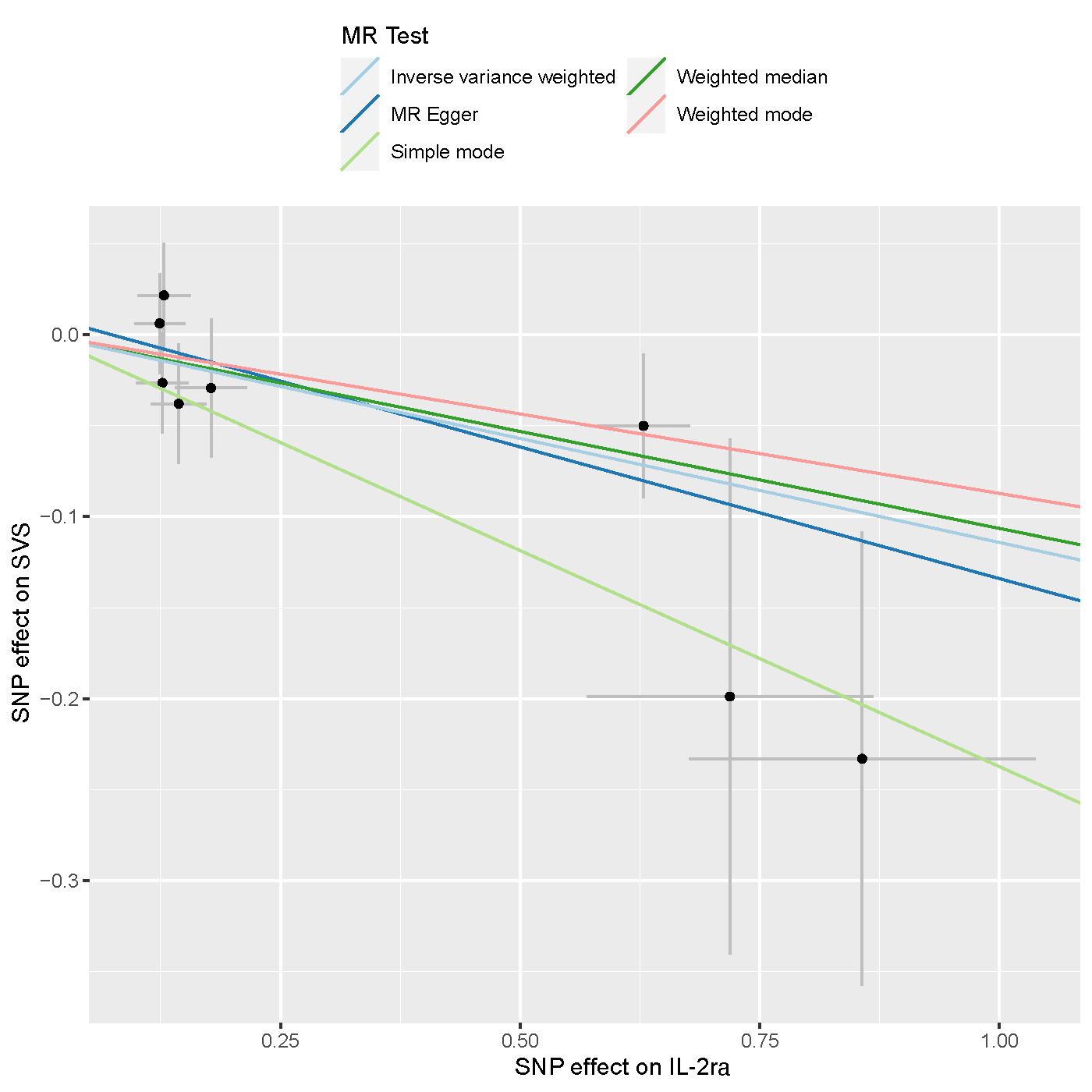

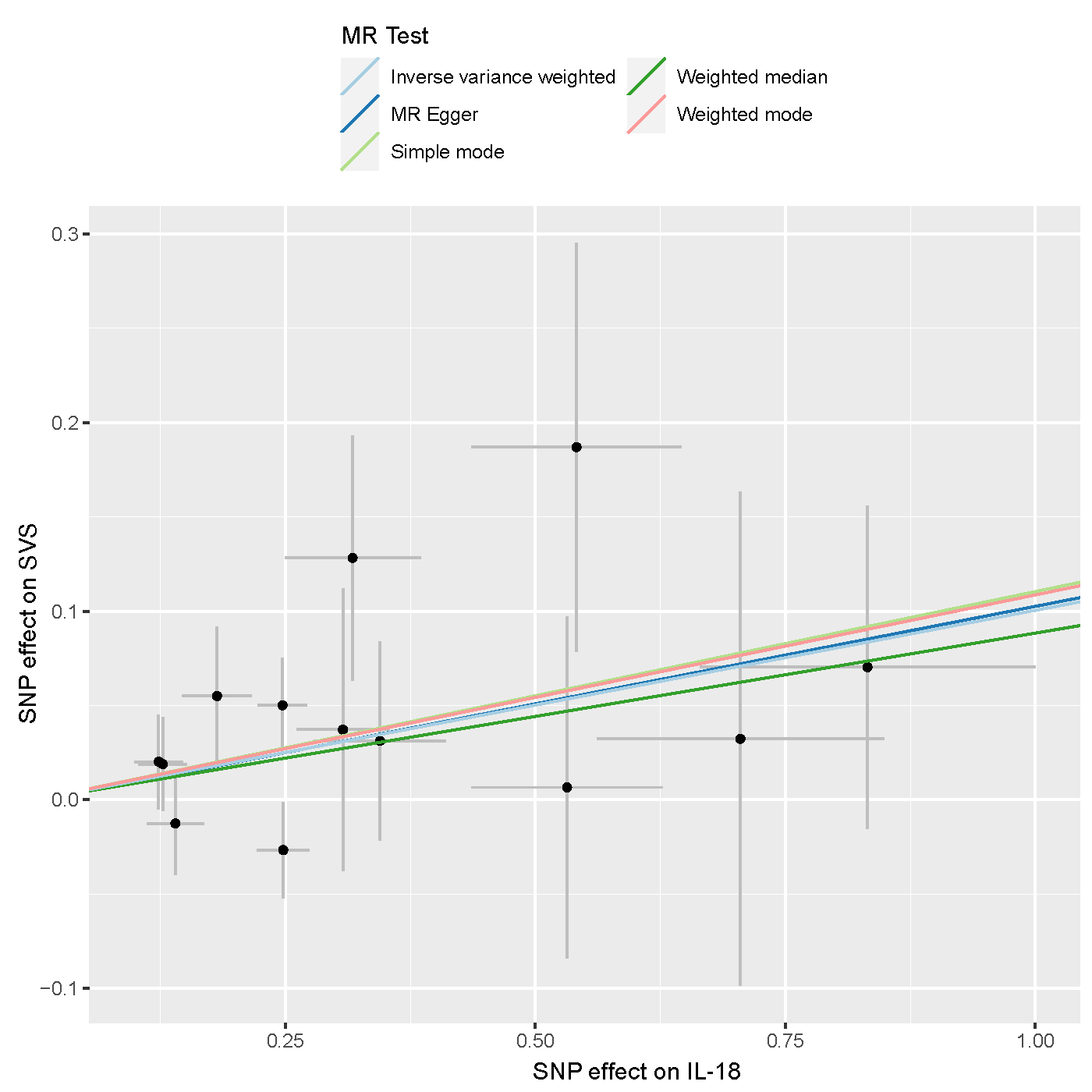
**E**


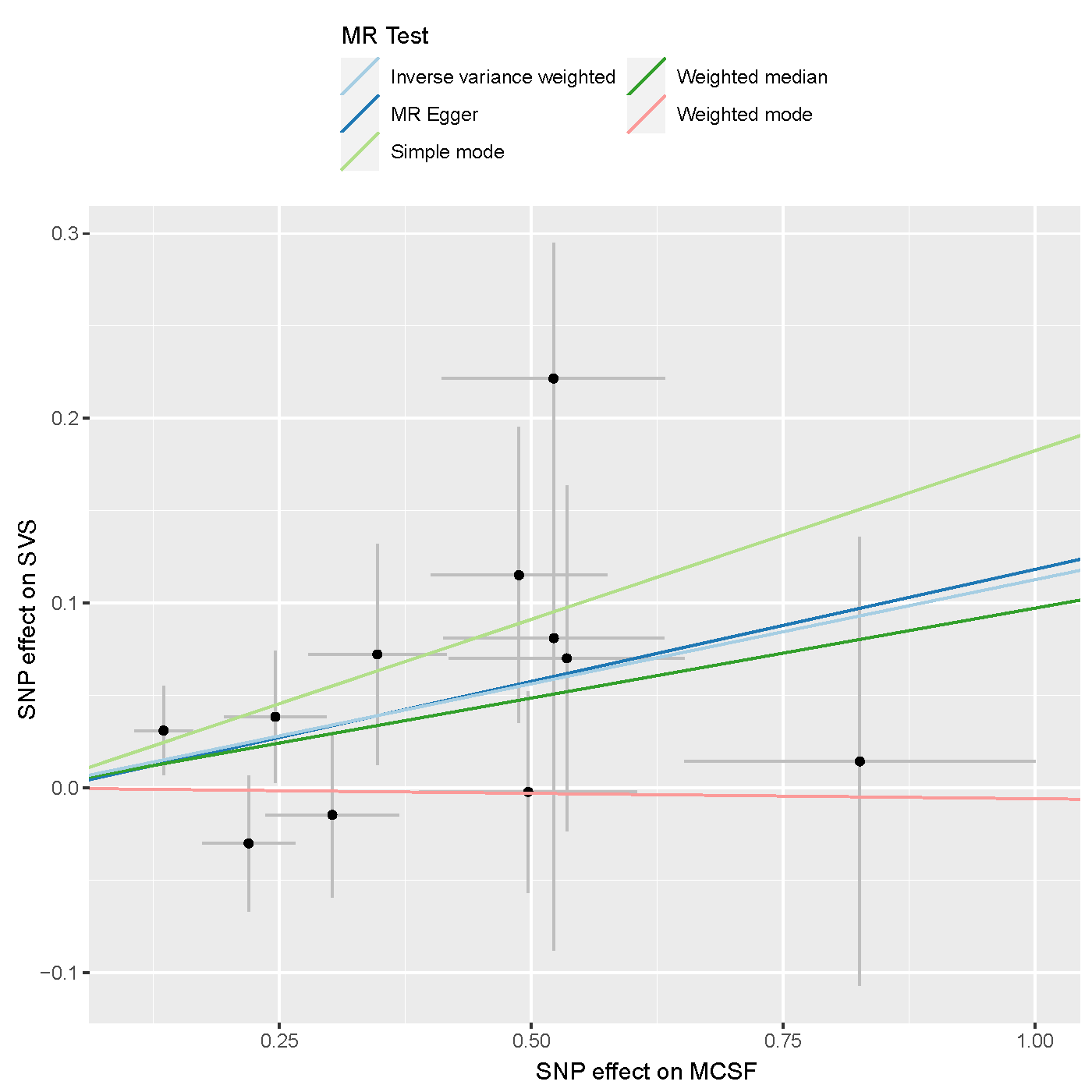


**Supplementary Figure 13.** (A) Scatter plots for the exposure of β-NGF (B) Scatter plots for the exposure of GROα (C) Scatter plots for the exposure of IL-2RA(D) Scatter plots for the exposure of IL-18(E) Scatter plots for the exposure of MCSF

**A B**


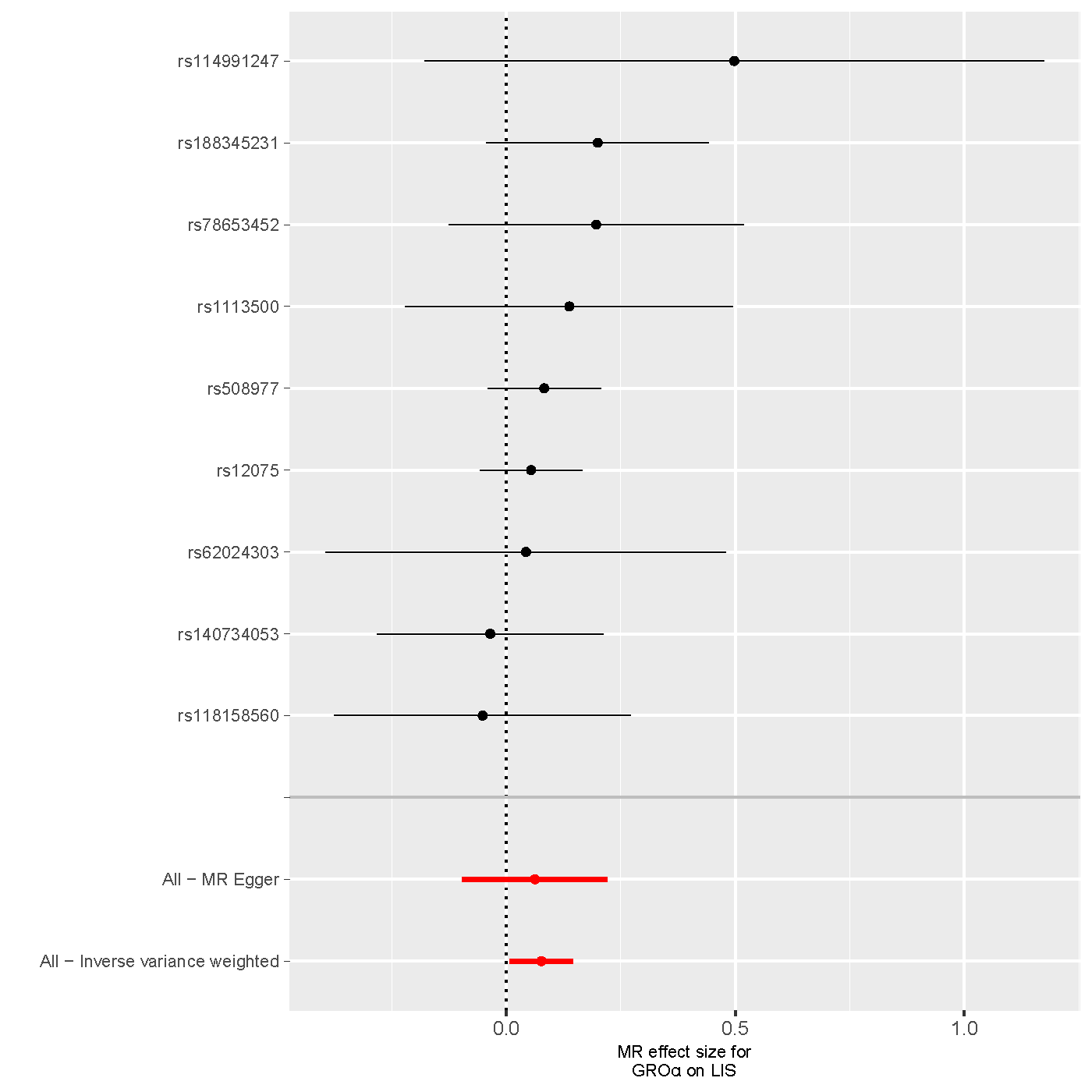

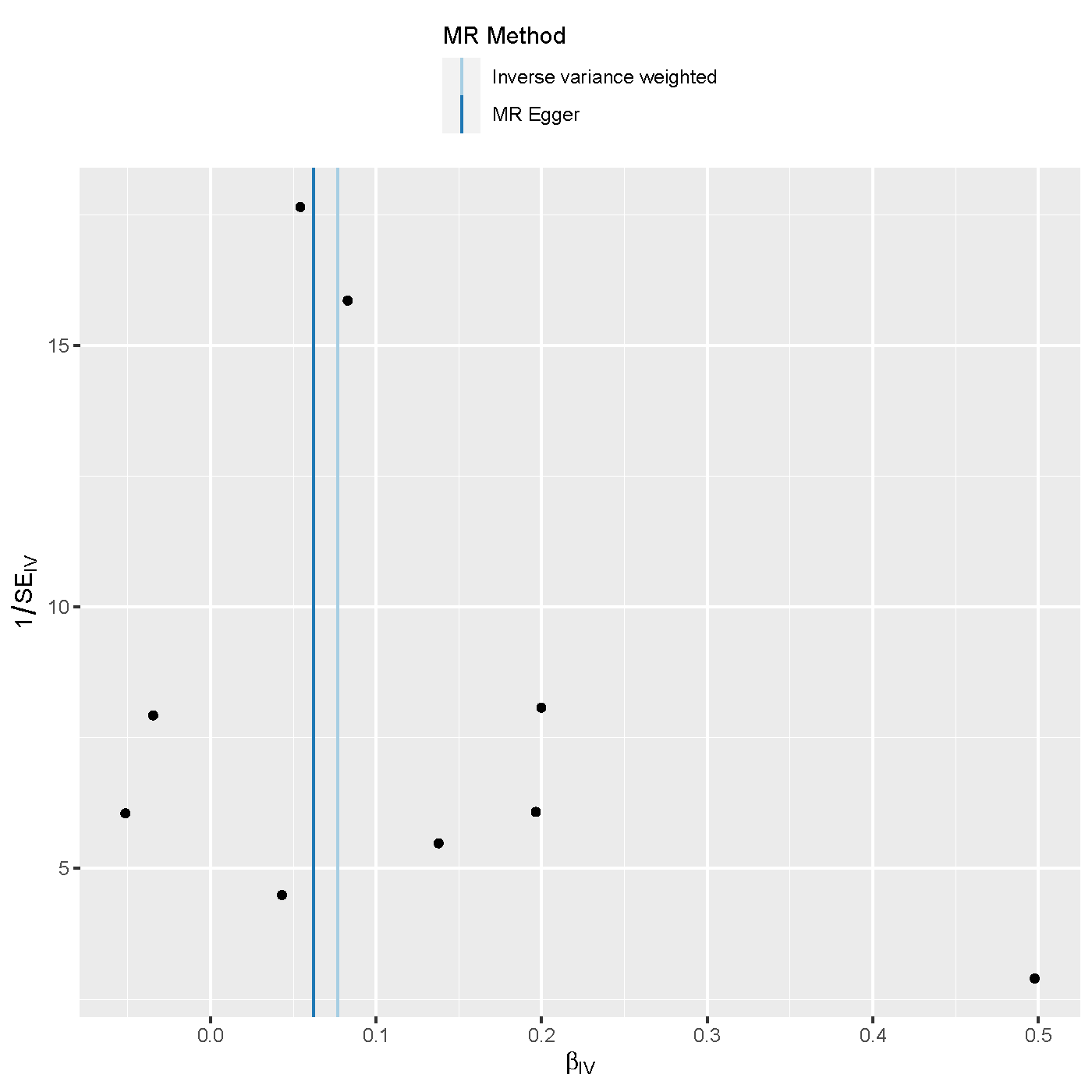
 **C D**


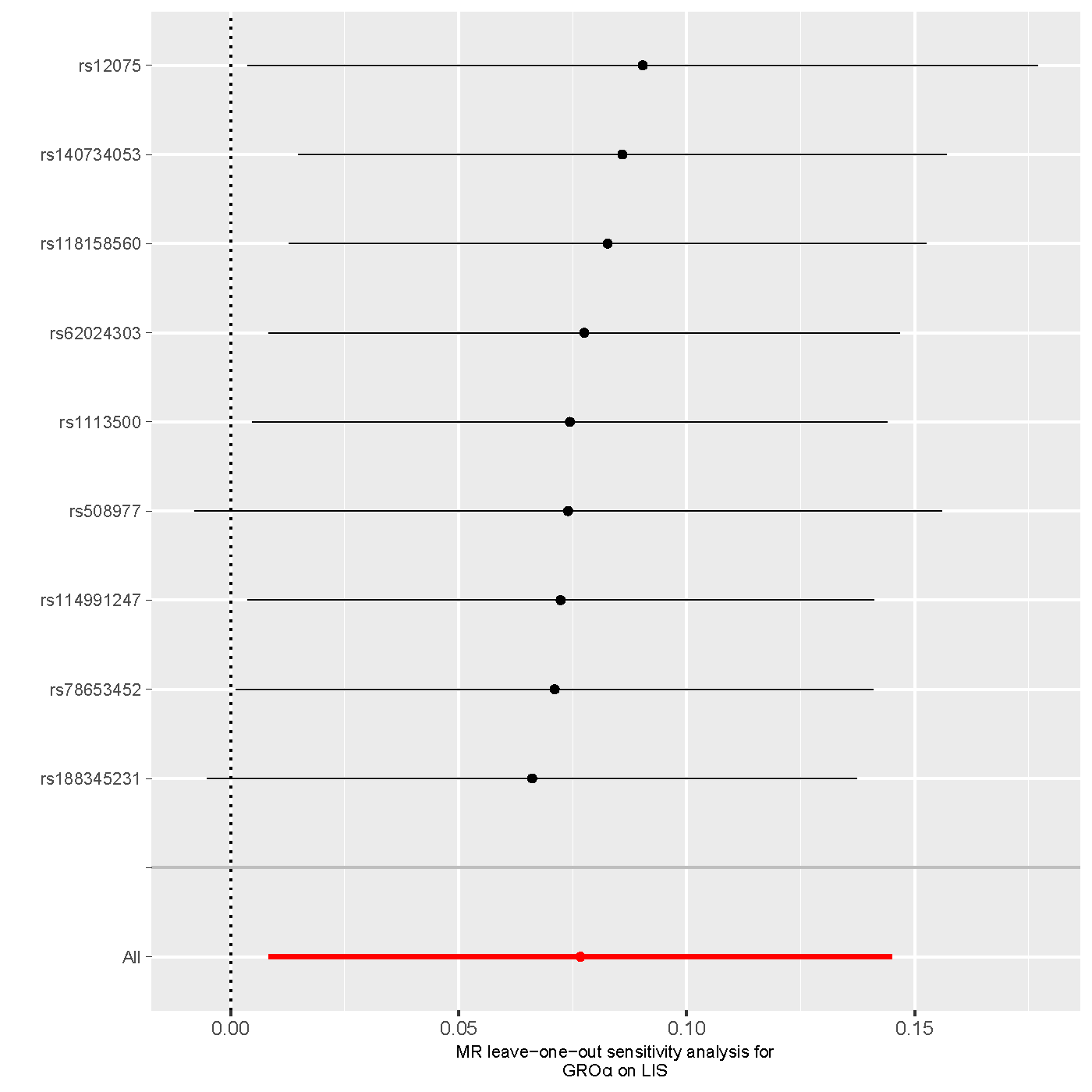

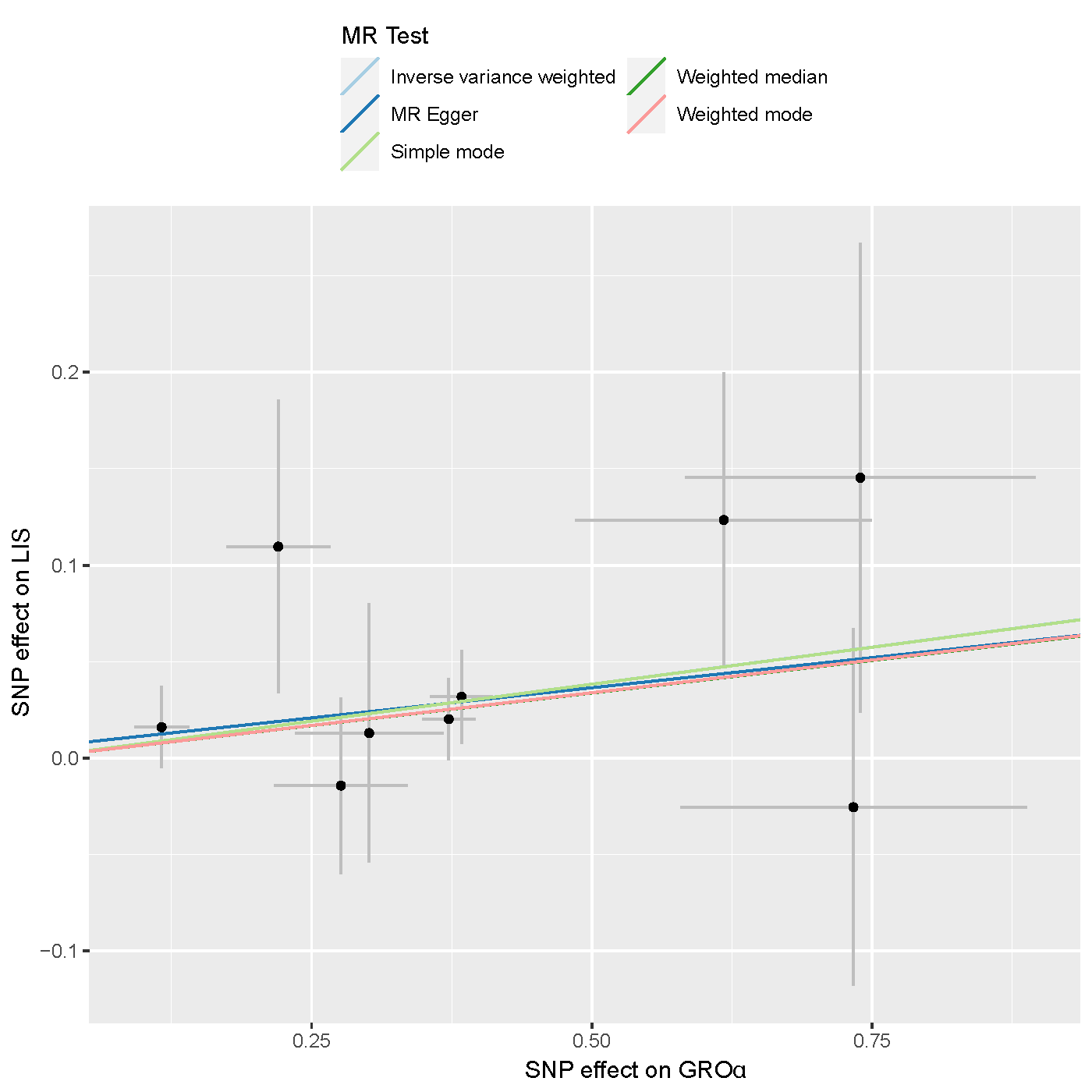


**Supplementary Figure 14.** (A) Forest plots for the exposure of GROα (B) Funnel plots for the exposure of GROα (C) Leave-one-out plots for the exposure of GROα (D)Scatter plots for the exposure of GROα

**A. B**


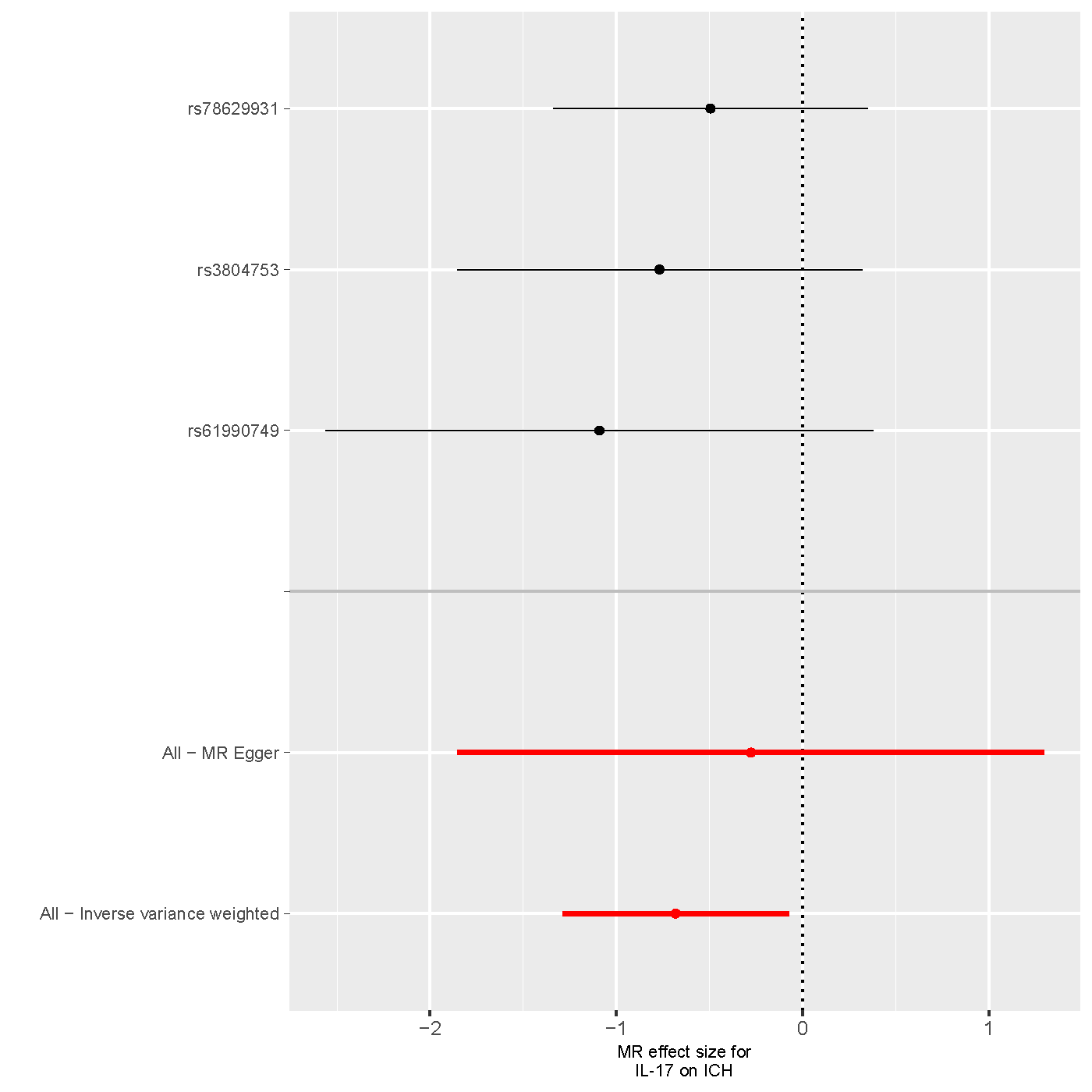

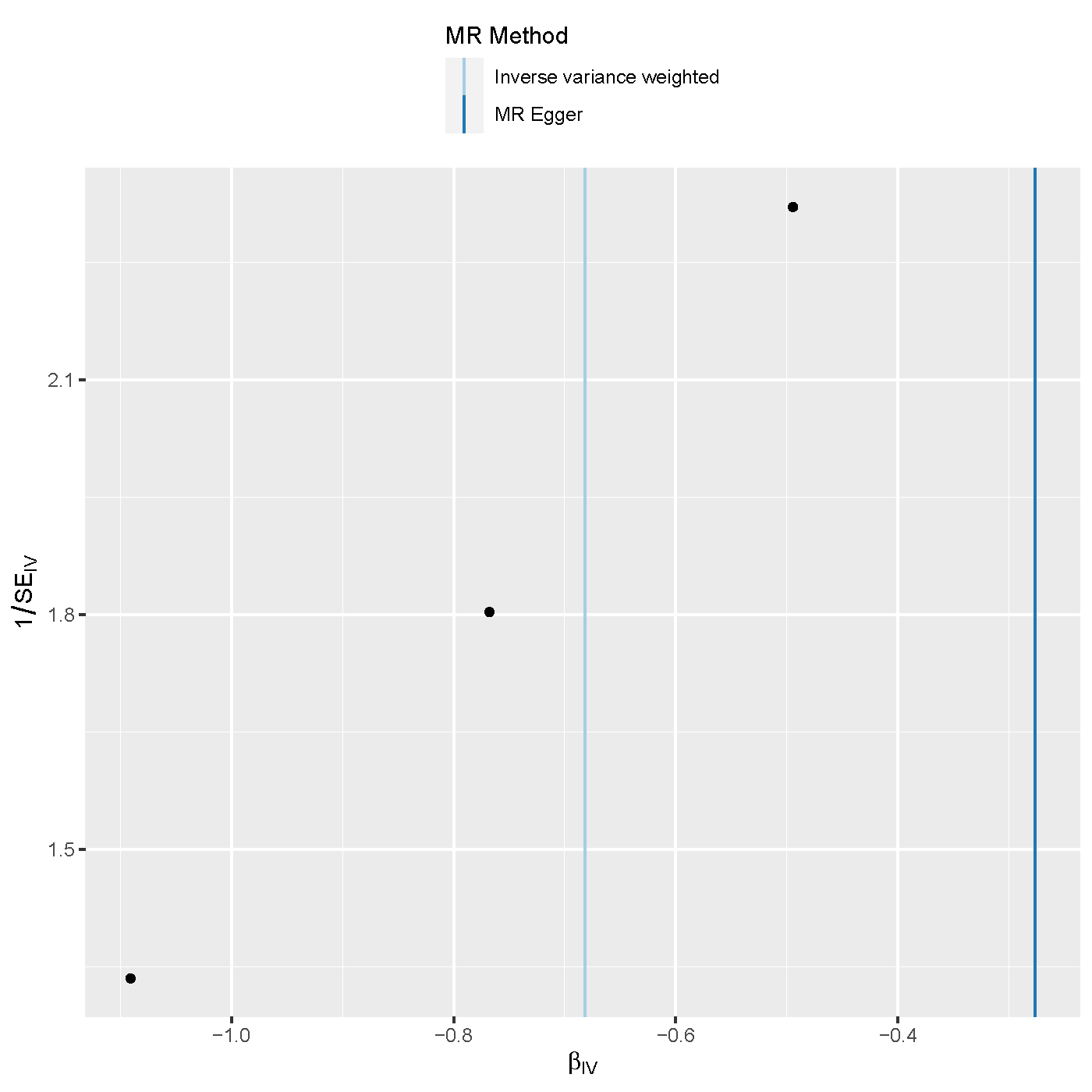
**C D**


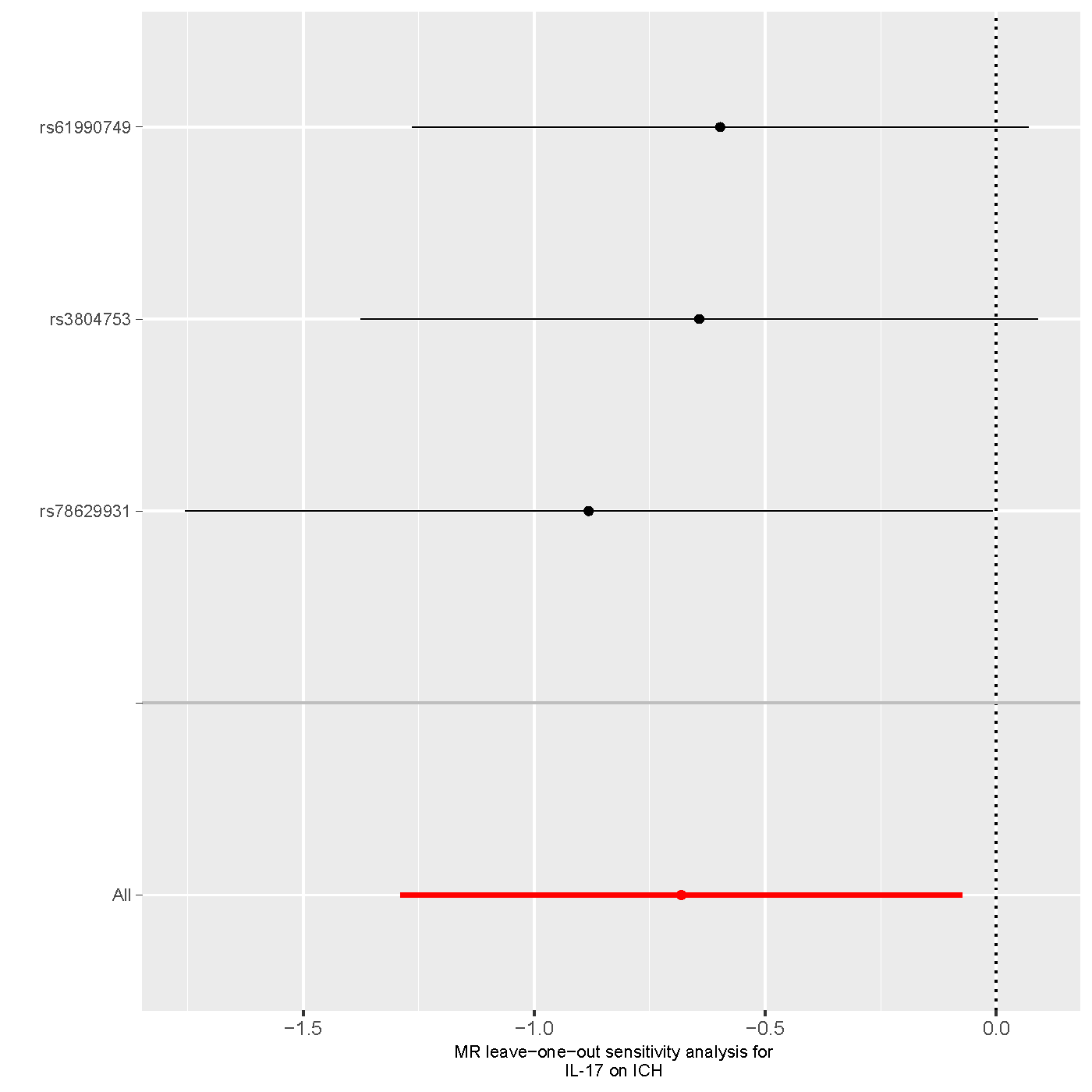

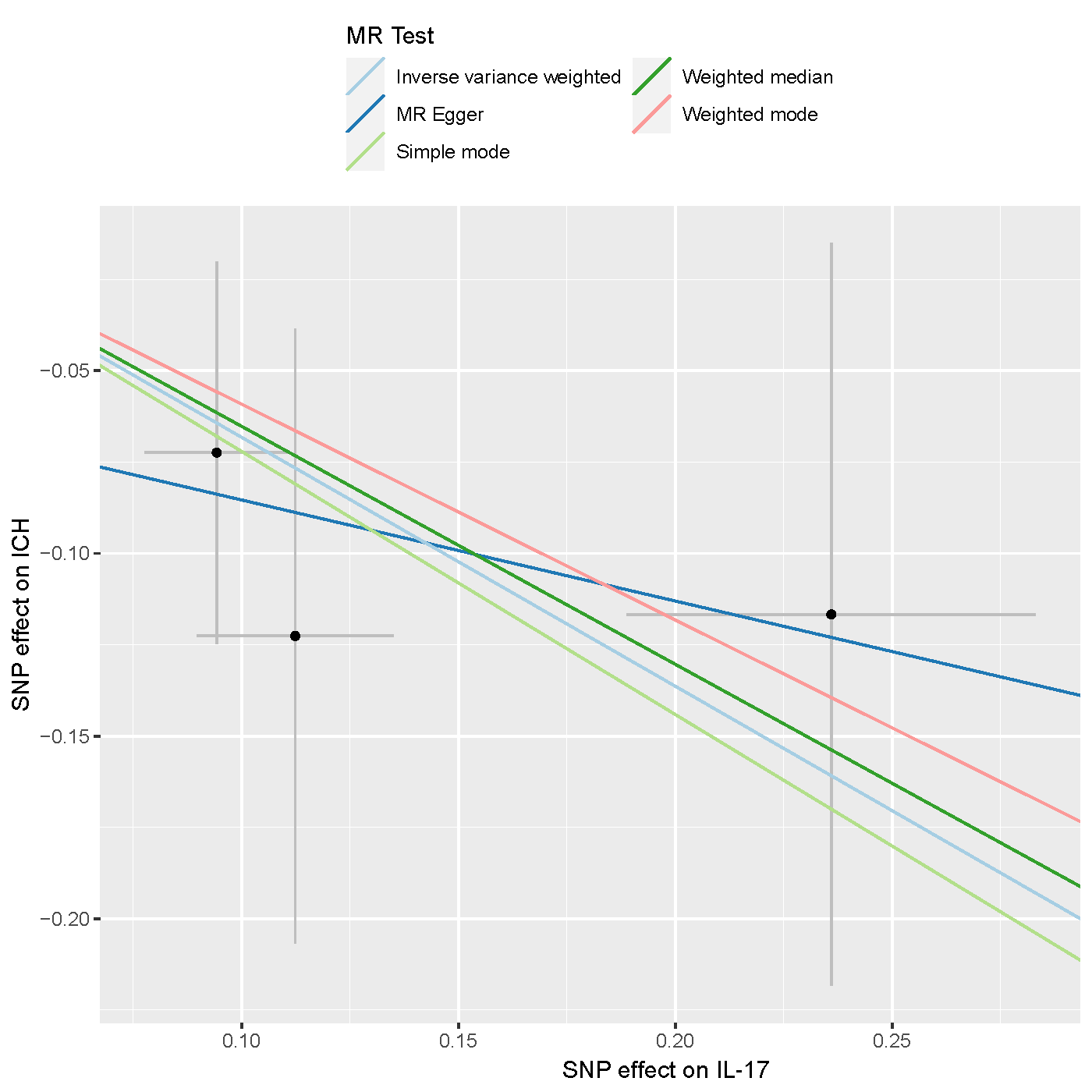


**Supplementary Figure 15.** (A) Funnel plots for the exposure of IL-17(B) Leave-one-out plots for the exposure of IL-17(C) Forest plots for the exposure of IL-17(D)Scatter plots for the exposure of IL-17

**A B**


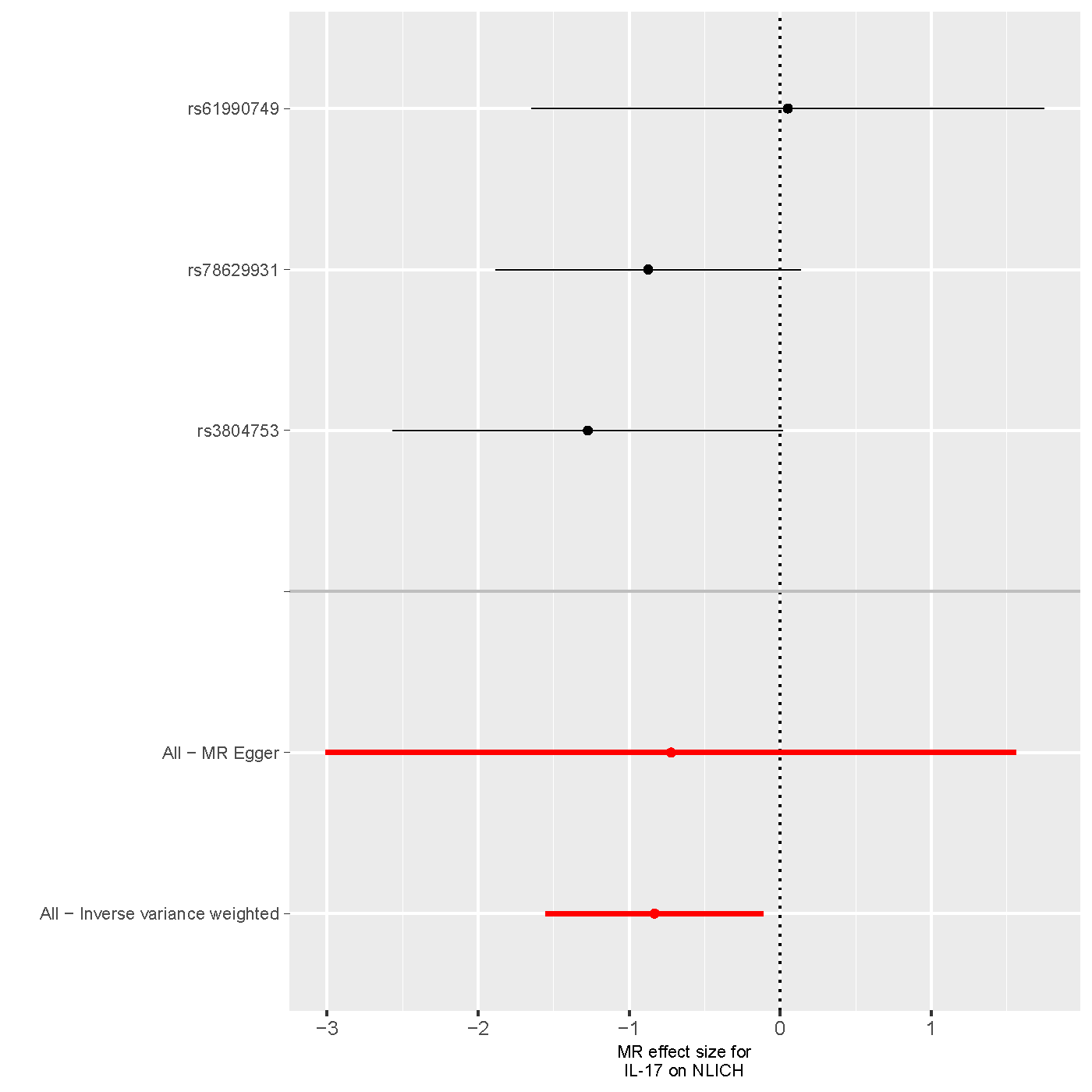

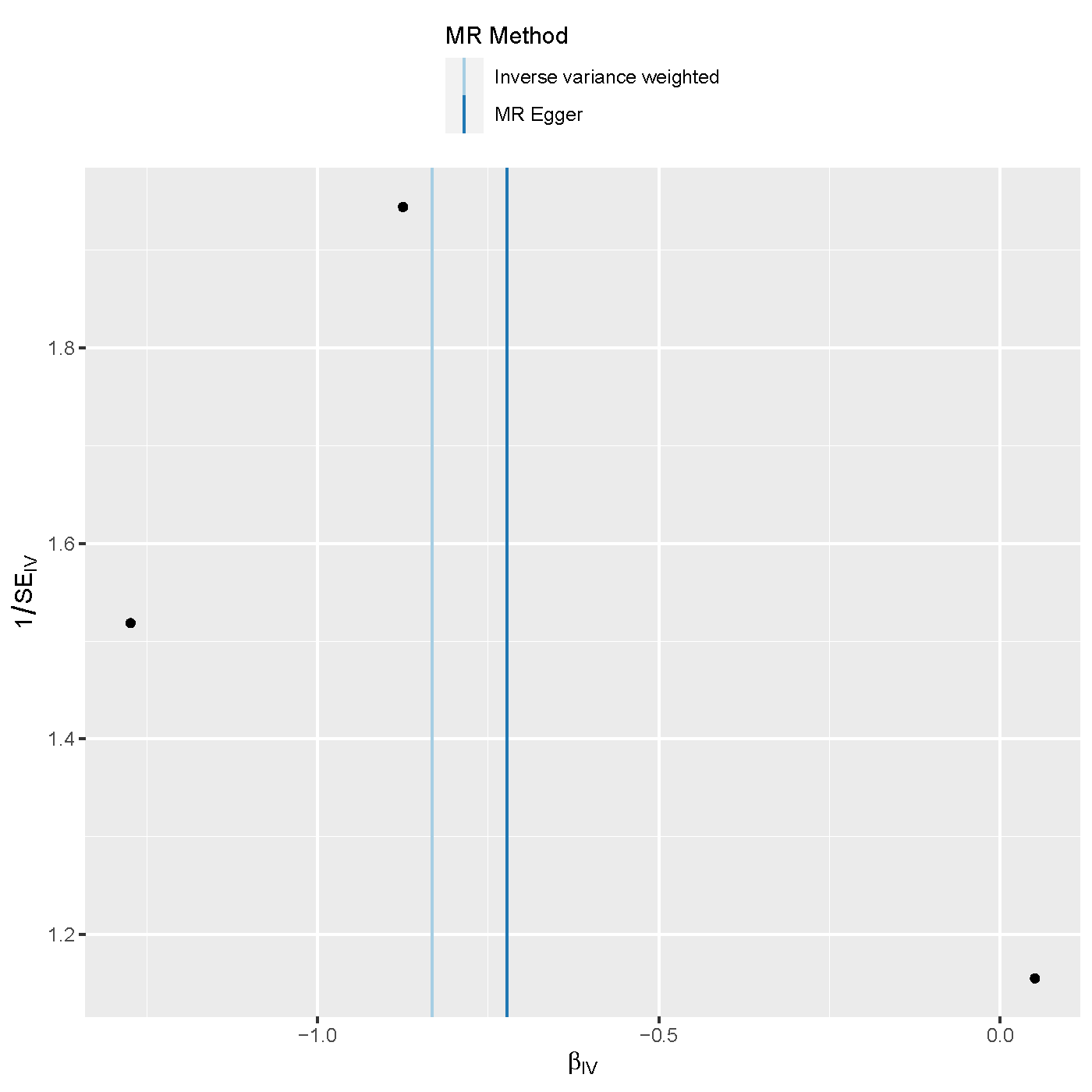


**C D**


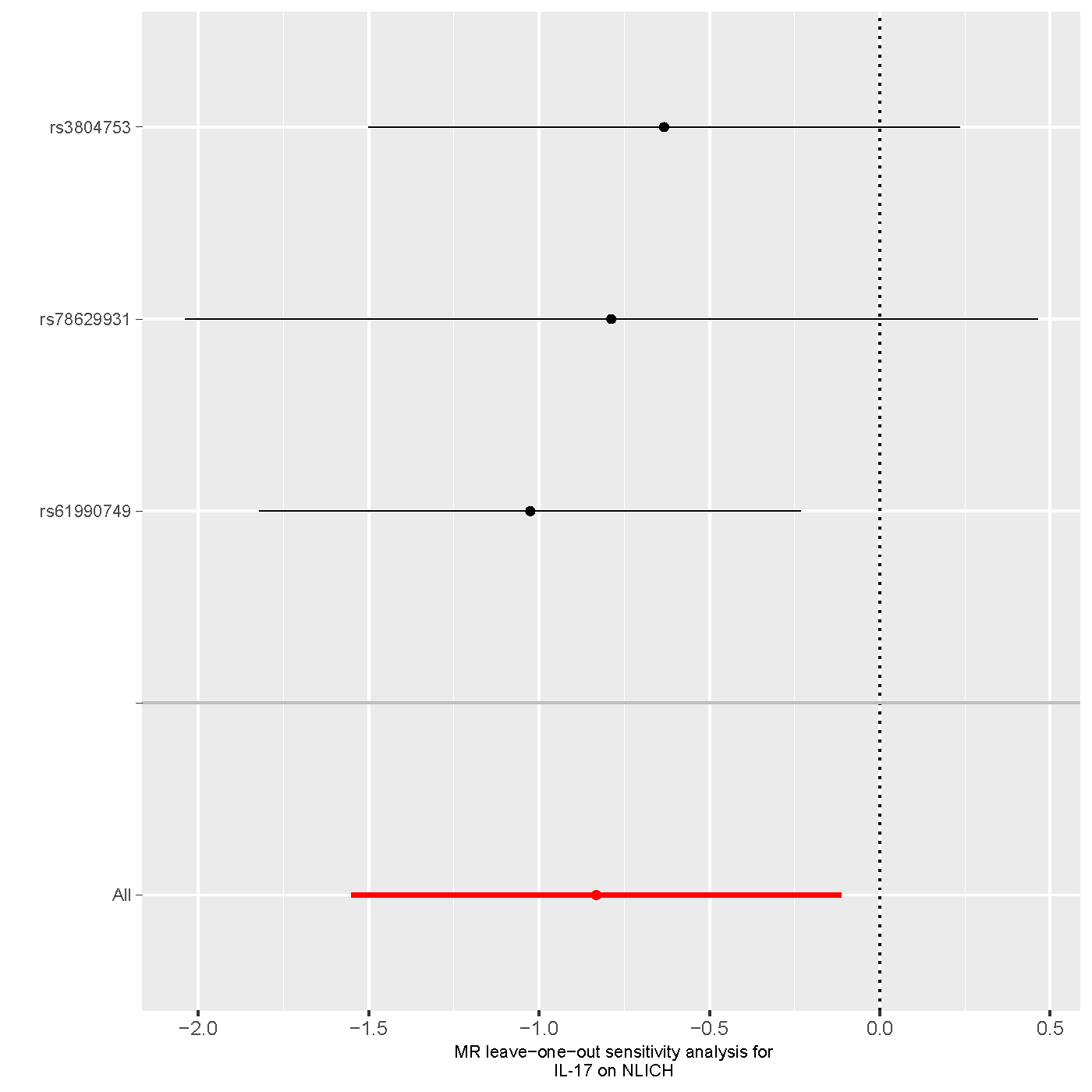

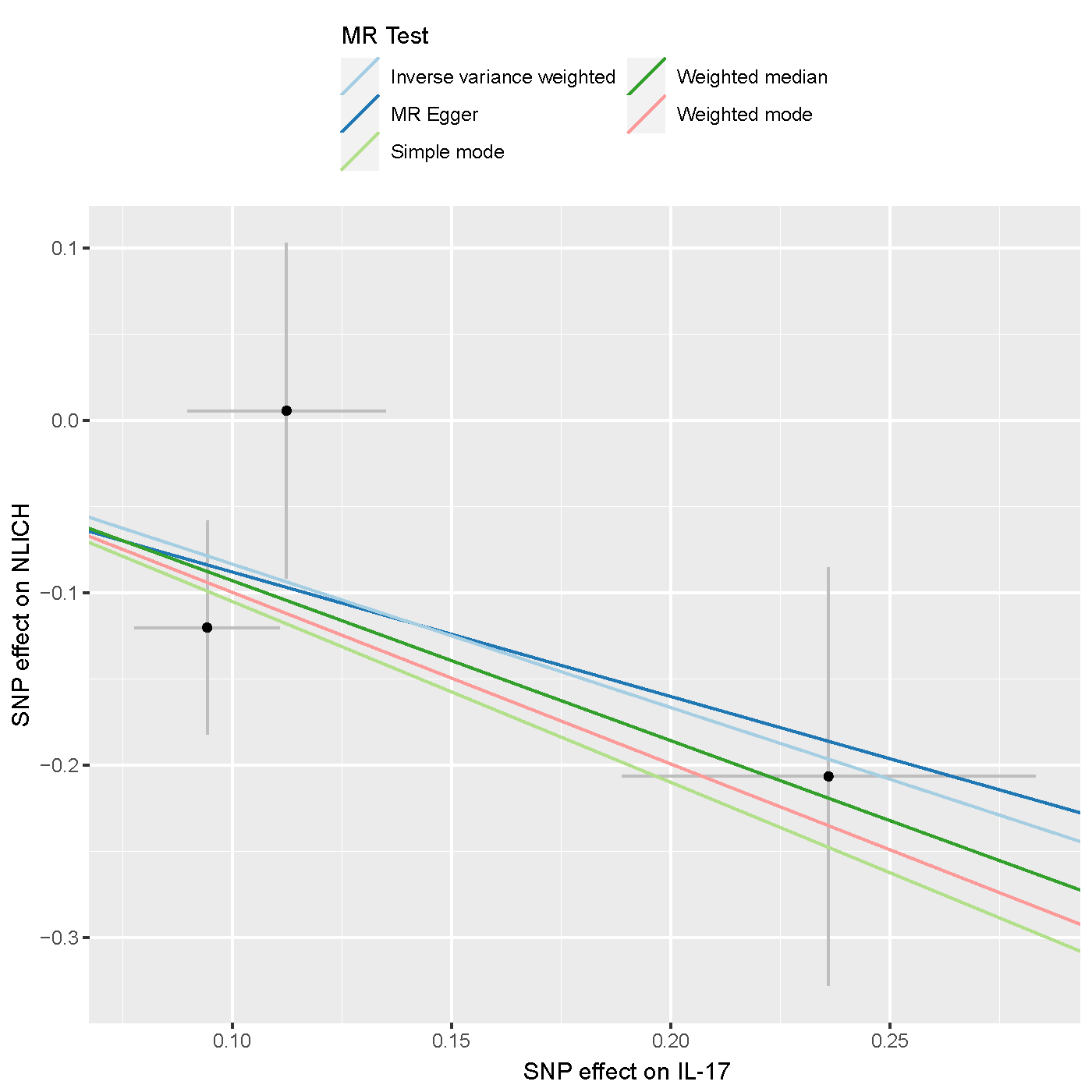


**Supplementary Figure 16.** (A) Forest plots for the exposure of IL-17 (B) Funnel plots for the exposure of IL-17 (C) Leave-one-out plots for the exposure of IL-17 (D)Scatter plots for the exposure of IL-17
